# Evaluation of type 2 diabetes genetic risk variants in Chinese adults: findings from 93,000 individuals from the China Kadoorie Biobank

**DOI:** 10.1007/s00125-016-3920-9

**Published:** 2016-04-06

**Authors:** Wei Gan, Robin G. Walters, Michael V. Holmes, Fiona Bragg, Iona Y. Millwood, Karina Banasik, Yiping Chen, Huaidong Du, Andri Iona, Anubha Mahajan, Ling Yang, Zheng Bian, Yu Guo, Robert J. Clarke, Liming Li, Mark I. McCarthy, Zhengming Chen, on behalf of the China Kadoorie Biobank Collaborative Group

**Affiliations:** Oxford Centre for Diabetes, Endocrinology, and Metabolism, University of Oxford, Churchill Hospital Campus, Old Road, Headington, Oxford, OX3 7LJ UK; Wellcome Trust Centre for Human Genetics, University of Oxford, Oxford, UK; Clinical Trial Service Unit and Epidemiological Studies Unit, University of Oxford, Old Road Campus, Oxford, OX3 7LF UK; The Novo Nordisk Foundation Center for Basic Metabolic Research, Section of Metabolic Genetics, University of Copenhagen, Copenhagen, Denmark; Chinese Academy of Medical Sciences, Dong Cheng District, Beijing, People’s Republic of China; School of Public Health, Peking University Health Sciences Center, Beijing, People’s Republic of China; National Institute of Health Research Oxford Biomedical Research Centre, Oxford, UK

**Keywords:** Biobank, Chinese, Genetic risk score, Population-based cohort studies, Type 2 diabetes, Winner’s curse

## Abstract

**Aims/hypothesis:**

Genome-wide association studies (GWAS) have discovered many risk variants for type 2 diabetes. However, estimates of the contributions of risk variants to type 2 diabetes predisposition are often based on highly selected case–control samples, and reliable estimates of population-level effect sizes are missing, especially in non-European populations.

**Methods:**

The individual and cumulative effects of 59 established type 2 diabetes risk loci were measured in a population-based China Kadoorie Biobank (CKB) study of 93,000 Chinese adults, including >7,100 diabetes cases.

**Results:**

Association signals were directionally consistent between CKB and the original discovery GWAS: of 56 variants passing quality control, 48 showed the same direction of effect (binomial test, *p* = 2.3 × 10^−8^). We observed a consistent overall trend towards lower risk variant effect sizes in CKB than in case–control samples of GWAS meta-analyses (mean 19–22% decrease in log odds, *p* ≤ 0.0048), likely to reflect correction of both ‘winner’s curse’ and spectrum bias effects. The association with risk of diabetes of a genetic risk score, based on lead variants at 25 loci considered to act through beta cell function, demonstrated significant interactions with several measures of adiposity (BMI, waist circumference [WC], WHR and percentage body fat [PBF]; all *p*_interaction_ < 1 × 10^−4^), with a greater effect being observed in leaner adults.

**Conclusions/interpretation:**

Our study provides further evidence of shared genetic architecture for type 2 diabetes between Europeans and East Asians. It also indicates that even very large GWAS meta-analyses may be vulnerable to substantial inflation of effect size estimates, compared with those observed in large-scale population-based cohort studies.

**Access to research materials:**

Details of how to access China Kadoorie Biobank data and details of the data release schedule are available from www.ckbiobank.org/site/Data+Access.

**Electronic supplementary material:**

The online version of this article (doi:10.1007/s00125-016-3920-9) contains peer-reviewed but unedited supplementary material, which is available to authorised users.

## Introduction

Type 2 diabetes affects ~400 million people globally [1]. The prevalence of type 2 diabetes has increased substantially in Asian populations, and in China it is estimated that 100 million adults (~11% of the adult population) are affected [2]. Lifestyle factors (e.g. physical inactivity), nutrition transitions and increased adiposity are the chief determinants of type 2 diabetes, but genetic factors also play an important role.

Genome-wide association studies (GWAS) and large-scale genotyping studies (e.g. MetaboChip and ExomeChip genotyping arrays) have identified more than 90 type 2 diabetes associated risk loci [3–28]. GWAS and replication studies conducted in a range of ancestry groups have revealed that most common-variant susceptibility loci are shared across ethnic groups [24, 29, 30]. While many type 2 diabetes susceptibility variants identified in Europeans have been successfully replicated in East Asians, failure to replicate (e.g. at *ADCY5*, *NOTCH2* and *PRC1*) likely arises from poor coverage by genotyping arrays, ethnic differences in allele frequency, variable linkage disequilibrium (LD) and limited statistical power.

As the number of type 2 diabetes associated variants has increased, so has the value in including genetic data in models to predict type 2 diabetes risk, weighting individual genetic variants according to their reported effect size [31]. However, effect estimates obtained from GWAS using case–control studies are often inflated due to spectrum bias and/or ‘winner’s curse’ [32, 33]. Spectrum bias describes the overestimation of test performance that can arise from studying ‘clear-cut’ cases or extremes of the underlying distribution (so-called ‘extreme phenotypes’) [32]. ‘Winner’s curse’ refers to the upward bias in the estimated effect of a newly identified variant, particularly when there is limited power to detect the true association [33]. To avoid these biases, large-scale population-based studies are required to obtain robust population-specific estimates of both individual and joint effects of GWAS-identified variants. The availability of such data remains limited, especially in non-European populations, and this motivated us to obtain population-based estimates of effect size in the China Kadoorie Biobank (CKB) study.

In addition, we constructed genetic risk scores (GRSs) to investigate the separate genetic effects on diabetes of SNPs that have been associated with beta cell dysfunction or insulin resistance (IR). The association between certain genetic variants and type 2 diabetes risk has been reported to vary according to obesity status [34], which could impact on the utility of predictive models. Therefore, we assessed whether associations of these GRSs with diabetes were modified in individuals with different degrees of adiposity.

## Methods

### Study population

The study sample consisted of 93,131 individuals with genotype data, randomly selected from the CKB study (www.ckbiobank.org), a prospective cohort of 512,891 Chinese adults. Details of the study design, protocol, procedures and characteristics of CKB have been described elsewhere [35]. Briefly, the baseline survey took place from June 2004 to July 2008 in ten geographically defined areas (5 urban, 5 rural) across China. In each study area, permanent residents were identified through official residential records, and invited to participate in the study. All participants are prospectively followed up for cause-specific mortality, morbidity and hospitalisation, using China CDC’s Disease Surveillance Points and linkages to the national health insurance claim databases [35]. Information about socio-demographic, lifestyle, medical history and current medication were collected by laptop-based questionnaires. Physical measurements were recorded including height, weight, waist and hip circumferences, and bio-impedance (Tanita BC-418MA, Tokyo, Japan). Except in one study area, where the protocol included fasting by all participants, initial screening for hyperglycaemia involved immediate on-site testing of non-fasting blood glucose using the SureStep Plus meter (LifeScan, Milpitas, CA, USA). Participants with non-fasting glucose levels ≥7.8 and <11.1 mmol/l were invited to return for a fasting blood glucose test the next day.

Diabetes was defined either as a self-report of physician diagnosis of diabetes or screen-detected diabetes, as previously reported [36]. For self-reported diabetes, those with an onset under age 30 and currently treated with insulin were considered as type 1 diabetes, and were excluded from the present analyses. Screen-detected diabetes was defined as no prior history of diabetes with a blood glucose level meeting any one of the following criteria, if applicable: (1) a random blood glucose level ≥7.0 mmol/l and a fasting time >8 h; (2) a random blood glucose level ≥11.1 mmol/l and a fasting time <8 h; (3) a fasting blood glucose level ≥7.0 mmol/l. For the current analysis, follow-up data were collected up to 31 December 2013 (Snapshot Database Release 9, April 2015). We combined all cases of prevalent (5,483) and incident (1,626) diabetes to give a total of 7,109 cases and 86,022 non-diabetes controls. All participants provided written informed consent for follow-up and long-term storage of biological samples.

### Genotyping

**Fig. 1:**
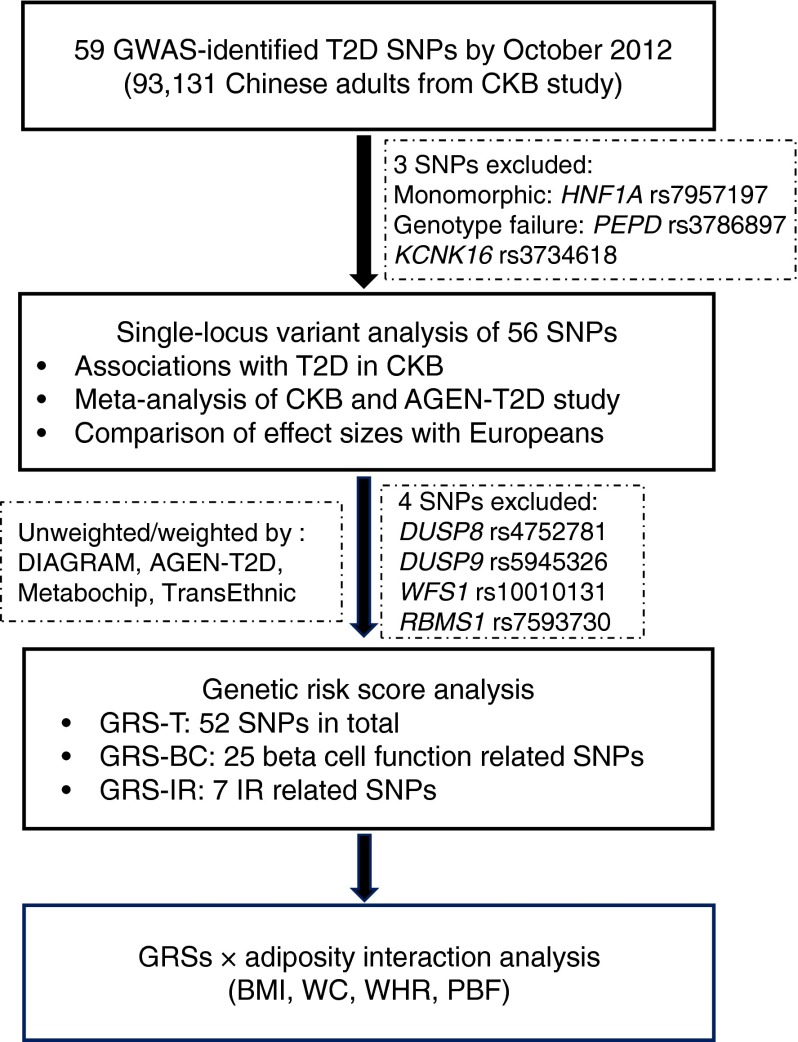
Flow chart of the analyses. T2D, type 2 diabetes

A panel of 384 single nucleotide polymorphisms (SNPs), selected on the basis of prior association with cardiovascular disease, risk factors and related phenotypes, were genotyped in 95,680 randomly selected individuals from CKB on the Illumina Golden Gate platform at the BGI laboratory in Shenzhen, China. A total of 93,131 individuals aged 30–79 years passed quality control criteria (call rate ≥98%, no sex mismatch, heterozygosity F statistic SD score <5). SNPs with low call rate (<95%) or Hardy–Weinberg disequilibrium (*p* < 0.05/384 = 1.3 × 10^−4^) were excluded. Mean genotyping concordance was 99.98% (range 98.66–100%) based on 2,063 duplicate samples included for quality control (QC) purposes. The SNP panel included 59 GWAS-identified type 2 diabetes risk variants reported by October 2012, of which five were originally reported in South Asians, 15 in East Asians and 36 in Europeans. These lead SNPs were selected based on the available association data from East Asian populations and/or fine-mapping data in Europeans at the time of array design and manufacturing. Since *HNF1A* rs12427353 is monomorphic in East Asians and genotyping of two variants (*PEPD* rs3786897, *KCNK16* rs3734618) failed QC, data were available for 56 variants (Fig. 1). The majority of SNPs were successfully genotyped in all selected samples except for four (*WFS1* rs10010131, *DGKB* rs2191349, *RASGRP1* rs7403531 and *GRK5* rs10886471), which were genotyped only in batches comprising subsets of the cohort (49%, 80%, 90% and 90% of participants, respectively). Estimates of relatedness based on 235 independent SNPs, using the R package SNPRelate [37], identified that 19% of participants had at least one first-degree relative among those genotyped. We present the results for the full dataset, but exclusion of 10,654 participants to eliminate first-degree relationships had no appreciable effect on individual results or our overall conclusions. Genomic inflation was estimated at *λ*_1000_ = 1.06–1.08, based on associations for 235 independent SNPs.

### GRSs

The risk variants at *DUSP8/INS*, *DUSP9* and *WFS1* were not included in GRS calculations because of parent-of-origin-specific effects, location on the X-chromosome and low genotyping rate, respectively. *RBMS1* rs7593730 was also excluded as it was associated with type 2 diabetes only in Europeans. The remaining 52 variants were selected for the overall GRS (GRS-T) (Fig. 1 and Electronic Supplementary Material [ESM] Tables 1, 2). Five types of weighted GRS (using weights derived from the natural logarithm of the per-allele OR) were calculated, using data from: (1) DIAGRAMv3 GWAS meta-analysis (DIAGRAMv3) [15]; (2) GWAS meta-analysis in East Asians (Asian Genetic Epidemiology Network-Type 2 Diabetes Consortium [AGEN-T2D]) [14]; (3) DIAGRAM Metabochip meta-analysis (Metabochip) [15]; (4) a trans-ethnic type 2 diabetes GWAS meta-analysis (TransEthnic) [24]; and (5) a combined meta-analysis of the CKB and trans-ethnic GWAS studies (TransEthnic + CKB) (ESM Fig. 1).

Type 2 diabetes risk variants were classified, based on previously published data concerning their pathophysiological mechanism, as being predominantly related to beta cell dysfunction, IR or neither (ESM Table 1). We updated the strategy proposed by Vassy et al [38] by including more lines of genetic and physiological evidence [15, 39–41]. Beta cell dysfunction related SNPs were identified by: (1) association with decreased HOMA of beta cell function (HOMA-B; *p* < 0.05, β for HOMA-B < 0 for risk allele) in non-diabetic individuals [15]; (2) association with one of the beta cell function indices during an OGTT (*p* < 0.05, β < 0 for risk allele) [40, 41]; (3) presence in a locus influencing beta cell function according to cluster analysis [40]; and/or (4) the existence of rare variants responsible for forms of monogenic diabetes characterised by insulin secretory failure (such as neonatal diabetes and MODY). IR-related SNPs were identified by: (1) association with increased HOMA-IR (*p* < 0.05, β > 0 for risk allele) in non-diabetic individuals [41] or decreased insulin sensitivity index (*p* < 0.05, β < 0 for risk allele) [15]; (2) association with fasting insulin (*p* < 0.05, β > 0) [39]; (3) presence in a locus influencing insulin sensitivity according to cluster analysis [40]; (4) association with increased triacylglycerol or other IR-related traits [39]; and (5) not acting primarily through obesity (*FTO* rs9939609, *MC4R* rs12970134) [15]. Thus, GRSs were constructed from 25 beta cell dysfunction related SNPs (GRS-BC) and seven IR-related SNPs (GRS-IR) (ESM Table 2). Missing genotypes were imputed by assigning the mean genotype for that participant’s regional centre. To make the weighted GRSs easier to interpret and more directly comparable to the unweighted score, values were rescaled as follows: GRS′ = GRS × total number of the risk alleles/(2 × sum of weights). Each point of the rescaled GRS thus corresponded to, on average, one additional risk allele.

### Statistical analysis

Departure from Hardy–Weinberg equilibrium was assessed using a 1-*df* χ^2^ test. For the primary outcome, logistic regression was used to estimate ORs and 95% CIs of individual variants and GRSs for combined prevalent/incident diabetes, adjusting for age, sex and regional centre. Comparison of effect sizes (log_e_ ORs) between CKB and previous studies was performed by inverse-variance weighted least squares regression through the origin. To combine our results with those from AGEN-T2D [14] or the TransEthnic meta-analysis [24], fixed effects meta-analysis was performed by inverse-variance weighting. We carefully checked the region of recruitment of the studies contributing to AGEN-T2D and found no evidence of overlap with CKB. Floating absolute risks were used to provide estimates of variance across GRS quartiles [42]. BMI cut-point categories were defined according to Asian criteria proposed by the WHO: normal weight (BMI < 23 kg/m^2^); overweight (23 ≤ BMI < 27.5 kg/m^2^); obese (BMI ≥ 27.5 kg/m^2^) [43]. Strata of waist circumference (WC), WHR and percentage body fat (PBF) were defined by sex-specific tertiles. Tests for interaction between adiposity and GRSs used logistic regression models including GRS, adiposity variable of interest and GRS × variable interaction term, with additional adjustment for age, sex and regional centre. Given that all SNPs were previously identified at GWAS significance for type 2 diabetes in Europeans and/or Asians, conventional Bonferroni correction would be overly conservative; we used the Holm–Bonferroni method or permutation procedures to control the family-wise error rate. For completeness, we also present findings using a 5% false discovery rate (Benjamini–Hochberg). In the meta-analyses, Cochran’s Q test was used to assess between-study heterogeneity and Bonferroni correction was used to account for multiple testing (*p* < 0.05/[55 × 3] = 3.0 × 10^−4^). The discriminative abilities of unweighted and weighted GRSs for risk of diabetes were assessed by receiver operating characteristic (ROC) curve analysis and compared using the DeLong test [44]. For 1000-fold cross-validation, weights were derived from a repeated analysis excluding randomly selected sets of 0.1% of the CKB sample, and using the resulting weights for the excluded individuals. The proportion of variance in phenotype explained for each SNP or GRS was calculated according to Shim et al [45] using previously reported means and SEs for SNP effect sizes [15]. We estimated power using ORs reported in the original GWAS and sample size and risk allele frequencies of our study with Quanto software (http://biostats.usc.edu/Quanto.html) (ESM Table 3). We investigated regional LD patterns among East Asians (CHB + JPT panel) and Europeans (CEU panel) from HapMap release 27 using the varLD algorithm [46], and presented results as Monte-Carlo *p* values from 10,000 iterations. All reported *p* values are nominal and 2-sided. Association analyses were performed using R software version 3.0.2 (www.r-project.org).

## Results

### Participant characteristics

Among the 93,131 CKB participants, there were 7,109 (7.6%) diabetes cases comprising 2,903 (3.1%) self-reported and 2,580 (2.8%) screen-detected at baseline, and 1,626 (1.7%) incident cases of diabetes that occurred during a mean (SD) of 7.1 (1.3) years follow-up (Table 1). A total of 86,022 participants without diabetes were considered controls. The overall mean BMI was 23.6 kg/m^2^. Women had slightly higher BMI than men, and also had higher prevalence and incidence of diabetes.

**Table 1:** Selected characteristics at baseline among 93,131 genotyped participants in CKB

Variable	Men	Women	All
Individuals, *n* (%)	37,677 (40.5)	55,454 (59.5)	93,131
Age, years	52.3 (10.8)	50.7 (10.5)	51.4 (10.7)
Random blood glucose^a^, mmol/l	6.0 (2.4)	6.2 (2.4)	6.1 (2.4)
WC, cm	82.0 (9.7)	79.1 (9.6)	80.3 (9.8)
Hip circumference, cm	90.6 (6.8)	91.1 (6.9)	90.9 (6.9)
BMI, kg/m^2^	23.4 (3.2)	23.8 (3.5)	23.6 (3.4)
WHR	0.90 (0.06)	0.87 (0.07)	0.88 (0.07)
PBF, %	21.9 (6.2)	32.1 (7.2)	28.1 (8.4)
Diabetes, *n* (%)	2,678 (7.1)	4,431 (8.0)	7,109 (7.6)
Clinically identified	1,101 (2.9)	1,802 (3.3)	2,903 (3.1)
Screen-detected	991 (2.6)	1,589 (2.9)	2,580 (2.8)
Incident	586 (1.6)	1,040 (1.9)	1,626 (1.8)

Data are means (SD) or *n* (%)

^a^Data only available for 92,166 participants (Men: 37,257; Women: 54,909)

### Association with individual variants in the CKB study

Table 2 shows the associations of 56 variants that passed QC with diabetes risk, together with the corresponding values from AGEN-T2D and a meta-analysis of CKB and AGEN-T2D. Risk allele frequencies observed in CKB were comparable with those in the 1000 Genomes Project Phase 3 CHB + CHS population. Sensitivity analysis showed that there was no evidence of heterogeneity between regional centres in the association of loci and risk of diabetes (ESM Tables 4-6). All SNPs were common in CKB (minor allele frequency, MAF > 0.05) except for variants at five loci: (*NOTCH2* rs10923931 [MAF = 0.032]; *THADA* rs7578597 [0.007]; *ADCY5* rs11708067 [0.003]; *TCF7L2* rs7901695 [0.031]; and *PRC1* rs8042680 [0.010]). Among the 56 variants, 48 had effects directionally consistent with those in the original reports (binomial test, *p* = 2.3 × 10^−8^) (ESM Tables 7, 8). Five SNPs reached GWAS significance (*p* < 5 × 10^−8^) and another 14 variants showed statistically significant association after multiple-testing correction (Holm–Bonferroni, *p* < 0.05); alternatively, association was replicated for 30 SNPs at 5% false discovery rate (Benjamini–Hochberg). All eight risk loci that were identified in East Asian and Chinese Han GWAS [14, 18] showed consistent effect directions. Among them, *MAEA* and *GLIS3* loci were significantly associated with type 2 diabetes after correction for multiple testing.

**Table 2:** Associations of previously identified type 2 diabetes susceptibility variants with risk of type 2 diabetes in CKB and meta-analysis in East Asian populations

CHR	SNP ID	Nearby genes	R/A	Risk allele frequency			CKB (up to 7,109 cases and 86,022 controls)			AGEN-T2D consortium (up to 25,079 cases and 29,611 controls)		Meta-analysis^a^ (CKB + AGEN-T2D) (up to 32,188 cases and 115,633 controls)			Reported population^b^
				CKB	CHB + CHS	CEU	OR (95% CI)	*p* value	*n*	OR (95% CI)	*p* value	OR (95% CI)	*p* value	*p*_hetero_^c^	
1	rs10923931	*NOTCH2*	T/G	0.032	0.041	0.081	1.16 (1.05, 1.27)	2.5 × 10^−3^	93,125	1.00 (0.86, 1.16)	9.90 × 10^−1^	1.11 (1.02, 1.20)	1.07 × 10^−2^	1.05 × 10^−1^	EU
1	rs340874	*PROX1*	C/T	0.391	0.394	0.525	1.05 (1.01, 1.09)	5.5 × 10^−3^	93,106	1.08 (1.03, 1.14)	2.84 × 10^−3^	1.06 (1.03, 1.09)	5.06 × 10^−5^*	3.44 × 10^−1^	EU
2	rs780094	*GCKR*	C/T	0.488	0.459	0.591	1.08 (1.04, 1.12)	1.3 × 10^−5^*	93,120	1.06 (1.01, 1.11)	2.10 × 10^−2^	1.07 (1.04, 1.10)	8.73 × 10^−7^*	4.96 × 10^−1^	EU
2	rs7578597	*THADA*	T/C	0.993	0.993	0.417	1.27 (1.01, 1.60)	4.4 × 10^−2^	93,131	0.93 (0.62, 1.40)	7.37 × 10^−1^	1.18 (0.96, 1.44)	1.12 × 10^−1^	1.97 × 10^−1^	EU
2	rs243021	*BCL11A*	A/G	0.670	0.644	0.480	1.07 (1.03, 1.11)	3.4 × 10^−4^*	93,125	1.05 (1.00, 1.10)	4.02 × 10^−2^	1.06 (1.03, 1.09)	5.56 × 10^−5^*	5.02 × 10^−1^	EU
2	rs7593730	*RBMS1*	C/T	0.836	0.808	0.818	0.98 (0.93, 1.02)	3.4 × 10^−1^	93,129	1.00 (0.94, 1.07)	9.20 × 10^−1^	0.99 (0.95, 1.02)	4.75 × 10^−1^	5.33 × 10^−1^	EU
2	rs3923113	*GRB14*	A/C	0.866	0.849	0.591	1.00 (0.95, 1.05)	9.7 × 10^−1^	93,088	1.03 (0.95, 1.12)	4.80 × 10^−1^	1.01 (0.97, 1.05)	6.73 × 10^−1^	5.44 × 10^−1^	SA
2	rs2943641	*IRS1*	C/T	0.925	0.928	0.662	1.04 (0.97, 1.11)	2.5 × 10^−1^	93,104	1.12 (1.03, 1.22)	1.11 × 10^−2^	1.07 (1.02, 1.13)	1.07 × 10^−2^	1.79 × 10^−1^	EU
3	rs1801282	*PPARG*	C/G	0.946	0.959	0.904	1.07 (0.99, 1.16)	1.0 × 10^−1^	93,126	1.15 (1.01, 1.30)	3.20 × 10^−2^	1.09 (1.02, 1.16)	1.22 × 10^−2^	3.54 × 10^−1^	EU
3	rs6780569	*UBE2E2*	G/A	0.798	0.779	0.909	1.11 (1.06, 1.16)	3.0 × 10^−6^*	93,127	1.17 (1.12, 1.22)	1.58 × 10^−11^	1.14 (1.10, 1.18)	8.32 × 10^−16^*	1.26 × 10^−1^	EA
3	rs831571	*PSMD6*	C/T	0.634	0.589	0.763	1.06 (1.02, 1.10)	1.5 × 10^−3^	93,094	1.09 (1.05, 1.13)	1.36 × 10^−6^	1.08 (1.05, 1.11)	1.38 × 10^−8^*	2.42 × 10^−1^	EA
3	rs4607103	*ADAMTS9*	C/T	0.638	0.608	0.783	1.00 (0.97, 1.04)	8.6 × 10^−1^	93,114	0.99 (0.95, 1.04)	6.74 × 10^−1^	1.00 (0.97, 1.03)	9.83 × 10^−1^	7.51 × 10^−1^	EU
3	rs11708067	*ADCY5*	A/G	0.997	0.993	0.783	1.92 (1.28, 2.88)	1.5 × 10^−3^	93,127	1.18 (0.80, 1.74)	4.04 × 10^−1^	1.49 (1.13, 1.97)	5.19 × 10^−3^	8.81 × 10^−2^	EU
3	rs1470579	*IGF2BP2*	C/A	0.257	0.250	0.308	1.11 (1.07, 1.16)	1.1 × 10^−7^*	93,108	1.15 (1.11, 1.19)	2.90 × 10^−13^	1.13 (1.10, 1.16)	4.21 × 10^−19^*	2.17 × 10^−1^	EU
3	rs16861329	*ST64GAL1*	C/G	0.809	0.764	0.869	1.04 (1.00, 1.09)	7.7 × 10^−2^	93,098	0.92 (0.86, 0.99)	1.80 × 10^−2^	1.01 (0.97, 1.04)	7.69 × 10^−1^	4.50 × 10^−3^	SA
4	rs6815464	*MAEA*	C/G	0.578	0.555	0.985	1.08 (1.04, 1.12)	3.4 × 10^−5^*	93,082	1.13 (1.10, 1.16)	1.57 × 10^−20^	1.11 (1.09, 1.13)	3.60 × 10^−22^*	3.64 × 10^−2^	EA
4	rs10010131	*WFS1*	G/A	0.938	0.925	0.646	1.04 (0.94, 1.15)	4.7 × 10^−1^	45,198	1.00 (0.91, 1.10)	9.92 × 10^−1^	1.02 (0.95, 1.09)	6.18 × 10^−1^	6.00 × 10^−1^	EU
5	rs4457053	*ZBED3*	G/A	0.052	0.055	0.298	1.10 (1.02, 1.18)	1.6 × 10^−2^	93,124	1.00 (0.85, 1.18)	9.77 × 10^−1^	1.08 (1.01, 1.16)	2.90 × 10^−2^	3.22 × 10^−1^	EU
6	rs7754840	*CDKAL1*	C/G	0.409	0.394	0.318	1.21 (1.17, 1.26)	3.6 × 10^−27^*	93,130	1.18 (1.14, 1.22)	2.94 × 10^−20^	1.20 (1.17, 1.23)	1.58 × 10^−45^*	2.65 × 10^−1^	EU
6	rs9470794	*ZFAND3*	C/T	0.316	0.339	0.116	1.02 (0.98, 1.05)	4.1 × 10^−1^	93,111	1.12 (1.08, 1.16)	2.06 × 10^−10^	1.07 (1.04, 1.10)	4.64 × 10^−7^*	2.39 × 10^−4^	EA
7	rs2191349	*DGKB*	T/G	0.654	0.654	0.535	1.05 (1.00, 1.09)	3.5 × 10^−2^	74,375	1.12 (1.08, 1.17)	3.88 × 10^−9^	1.09 (1.06, 1.12)	8.69 × 10^−9^*	1.51 × 10^−2^	EU
7	rs864745	*JAZF1*	T/C	0.766	0.786	0.505	1.04 (1.00, 1.09)	3.8 × 10^−2^	93,120	1.06 (1.00, 1.12)	3.50 × 10^−2^	1.05 (1.01, 1.09)	4.65 × 10^−3^	7.21 × 10^−1^	EU
7	rs4607517	*GCK*	A/G	0.211	0.195	0.207	1.01 (0.97, 1.06)	5.5 × 10^−1^	93,113	1.03 (0.97, 1.09)	3.97 × 10^−1^	1.02 (0.98, 1.05)	3.02 × 10^−1^	6.84 × 10^−1^	EU
7	rs6467136	*GCC1*-*PAX4*	G/A	0.784	0.776	0.520	1.04 (1.00, 1.09)	6.5 × 10^−2^	93,018	1.11 (1.07, 1.14)	4.96 × 10^−11^	1.08 (1.05, 1.11)	1.58 × 10^−9^*	2.92 × 10^−2^	EA
7	rs972283	*KLF14*	G/A	0.710	0.697	0.540	1.04 (1.00, 1.08)	4.2 × 10^−2^	93,126	0.99 (0.93, 1.06)	8.52 × 10^−1^	1.03 (0.99, 1.06)	1.00 × 10^−1^	2.22 × 10^−1^	EU
8	rs896854	*TP53INP1*	T/C	0.308	0.303	0.429	1.04 (1.00, 1.08)	4.4 × 10^−2^	93,127	1.07 (1.02, 1.12)	9.05 × 10^−3^	1.05 (1.02, 1.08)	9.15 × 10^−4^*	3.59 × 10^−1^	EU
8	rs13266634	*SLC30A8*	C/T	0.538	0.529	0.758	1.10 (1.06, 1.13)	3.4 × 10^−8^*	92,535	1.10 (1.07, 1.14)	4.04 × 10^−8^	1.10 (1.07, 1.13)	7.89 × 10^−15^*	7.78 × 10^−1^	EU
9	rs7041847	*GLIS3*	A/G	0.463	0.459	0.556	1.07 (1.03, 1.10)	2.9 × 10^−4^*	92,708	1.10 (1.07, 1.13)	1.99 × 10^−14^	1.09 (1.06, 1.11)	2.86 × 10^−14^*	1.76 × 10^−1^	EA
9	rs17584499	*PTPRD*	T/C	0.101	0.091	0.202	1.00 (0.95, 1.06)	9.3 × 10^−1^	93,019	1.09 (1.00, 1.19)	4.00 × 10^−2^	1.03 (0.98, 1.08)	2.48 × 10^−1^	1.13 × 10^−1^	EA
9	rs10811661	*CDKN2A/B*	T/C	0.543	0.575	0.803	1.22 (1.18, 1.26)	7.1 × 10^−28^*	93,062	1.12 (1.07, 1.16)	1.49 × 10^−7^	1.17 (1.14, 1.21)	1.23 × 10^−31^*	1.29 × 10^−3^	EU
9	rs13292136	*TLE4/CHCHD9*	C/T	0.909	0.923	0.934	1.08 (1.01, 1.14)	2.2 × 10^−2^	93,118	0.99 (0.92, 1.07)	8.84 × 10^−1^	1.04 (0.99, 1.09)	1.01 × 10^−1^	1.06 × 10^−1^	EU
10	rs10906115	*CDC123*	A/G	0.626	0.654	0.641	1.08 (1.05, 1.12)	1.1 × 10^−5^*	93,076	1.08 (1.05, 1.13)	1.65 × 10^−5^	1.08 (1.06, 1.11)	7.11 × 10^−10^*	9.97 × 10^−1^	EA
10	rs1802295	*VPS26A*	T/C	0.109	0.096	0.338	1.03 (0.97, 1.08)	3.8 × 10^−1^	93,048	1.01 (0.94, 1.09)	8.00 × 10^−1^	1.02 (0.98, 1.07)	3.73 × 10^−1^	7.89 × 10^−1^	SA
10	rs1111875	*HHEX/IDE*	C/T	0.279	0.279	0.581	1.11 (1.07, 1.15)	6.2 × 10^−8^*	93,093	1.08 (1.04, 1.13)	8.67 × 10^−5^	1.10 (1.07, 1.13)	3.47 × 10^−11^*	3.81 × 10^−1^	EU
10	rs7901695	*TCF7L2*	C/T	0.031	0.026	0.328	1.37 (1.25, 1.50)	6.5 × 10^−12^*	92,365	1.18 (1.03, 1.35)	1.60 × 10^−2, d^	1.31 (1.21, 1.41)	1.91 × 10^−12^*	6.93 × 10^−2^	EU
10	rs10886471	*GRK5*	C/T	0.794	0.774	0.455	1.00 (0.96, 1.05)	9.0 × 10^−1^	84,095	1.06 (0.99, 1.13)	1.00 × 10^−1^	1.02 (0.98, 1.06)	2.97 × 10^−1^	1.93 × 10^−1^	EA
11	rs4752781	*DUSP8/INS*	T/A	0.833	0.813	0.465	0.99 (0.95, 1.04)	7.3 × 10^−1^	92,932	1.04 (0.98, 1.10)	1.70 × 10^−1,e^	1.01 (0.97, 1.05)	5.96 × 10^−1^	2.27 × 10^−1^	EU
11	rs2237892	*KCNQ1*	C/T	0.676	0.656	0.924	1.25 (1.20, 1.30)	4.6 × 10^−30^*	92,991	1.19 (1.14, 1.24)	3.62 × 10^−18^	1.22 (1.19, 1.26)	8.65 × 10^−46^*	7.70 × 10^−2^	EA
11	rs5215	*KCNJ11*	C/T	0.386	0.387	0.384	1.07 (1.04, 1.11)	7.8 × 10^−5^*	93,120	1.10 (1.06, 1.14)	2.28 × 10^−7^	1.09 (1.06, 1.11)	1.22 × 10^−10^*	3.69 × 10^−^1	EU
11	rs1552224	*ARAP1*	A/C	0.916	0.909	0.884	1.09 (1.02, 1.16)	1.1 × 10^−2^	93,130	1.16 (1.05, 1.28)	2.50 × 10^−3^	1.11 (1.05, 1.17)	1.85 × 10^−4^*	2.92 × 10^−1^	EU
11	rs10830963	*MTNR1B*	G/C	0.428	0.413	0.258	1.02 (0.99, 1.06)	2.0 × 10^−1^	93,107	1.00 (0.93, 1.08)	9.50 × 10^−1^	1.02 (0.99, 1.05)	2.40 × 10^−1^	6.25 × 10^−1^	EU
12	rs1531343	*HMGA2*	C/G	0.098	0.118	0.106	1.05 (0.99, 1.11)	9.9 × 10^−2^	92,189	1.06 (0.99, 1.14)	1.05 × 10^−1^	1.05 (1.01, 1.10)	1.94 × 10^−2^	7.83 × 10^−1^	EU
12	rs7961581	*TSPAN8/LGR5*	C/T	0.215	0.204	0.263	1.04 (1.00, 1.08)	7.1 × 10^−2^	93,114	1.01 (0.95, 1.06)	8.49 × 10^−1^	1.03 (0.99, 1.06)	1.31 × 10^−1^	3.18 × 10^−1^	EU
13	rs1359790	*SPRY2*	G/A	0.716	0.685	0.732	1.06 (1.02, 1.10)	2.5 × 10^−3^	93,096	1.05 (1.01, 1.10)	1.02 × 10^−2^	1.06 (1.03, 1.09)	7.73 × 10^−5^*	7.66 × 10^−1^	EA
15	rs7403531	*RASGRP1*	T/C	0.350	0.315	0.278	1.03 (0.99, 1.07)	9.3 × 10^−2^	84,075	1.08 (1.02, 1.13)	3.80 × 10^−3^	1.05 (1.02, 1.08)	2.95 × 10^−3^	2.40 × 10^−1^	EA
15	rs7172432	*VPS13C*	A/G	0.618	0.627	0.591	1.07 (1.03, 1.11)	4.3 × 10^−4^*	93,095	1.11 (1.07, 1.15)	2.86 × 10^−8^	1.09 (1.06, 1.11)	1.47 × 10^−10^*	1.62 × 10^−1^	EA
15	rs7178572	*HMG20A*	G/A	0.350	0.382	0.687	1.07 (1.04, 1.11)	1.3 × 10^−4^*	93,127	1.09 (1.04, 1.14)	4.40 × 10^−4^	1.08 (1.05, 1.11)	1.43 × 10^−7^*	6.41 × 10^−1^	SA
15	rs11634397	*ZFAND6*	G/A	0.088	0.077	0.657	1.02 (0.96, 1.09)	4.5 × 10^−1^	93,115	1.00 (0.90, 1.11)	9.90 × 10^−1^	1.02 (0.97, 1.07)	5.14 × 10^−1^	6.95 × 10^−1^	EU
15	rs2028299	*AP3S2*	C/A	0.202	0.185	0.258	1.06 (1.02, 1.11)	6.0 × 10^−3^	93,115	1.08 (1.02, 1.14)	1.30 × 10^−2^	1.07 (1.03, 1.11)	1.45 × 10^−4^*	6.72 × 10^−1^	SA
15	rs8042680	*PRC1*	A/C	0.990	0.998	0.283	0.88 (0.74, 1.05)	1.5 × 10^−1^	93,128	1.64 (1.16, 2.32)	4.92 × 10^−3^	1.00 (0.85, 1.16)	9.61 × 10^−1^	1.60 × 10^−3^	EU
16	rs9939609	*FTO*	A/T	0.124	0.147	0.444	1.15 (1.09, 1.21)	5.4 × 10^−8^*	93,123	1.13 (1.07, 1.18)	5.26 × 10^−7^	1.14 (1.10, 1.18)	1.76 × 10^−13^ *	5.16 × 10^−1^	EU
17	rs4523957	*SRR*	T/G	0.707	0.702	0.641	0.98 (0.94, 1.02)	3.2 × 10^−1^	90,663	1.03 (0.97, 1.09)	2.70 × 10^−1^,^f^	0.99 (0.96, 1.03)	7.61 × 10^−1^	1.85 × 10^−1^	EA
17	rs4430796	*HNF1B*	G/A	0.279	0.260	0.475	1.09 (1.05, 1.14)	3.8 × 10^−6^*	93,089	1.12 (1.05, 1.19)	8.30 × 10^−4^	1.10 (1.07, 1.14)	8.48 × 10^−9^*	5.66 × 10^−1^	EU
18	rs12970134	*MC4R*	A/G	0.188	0.173	0.288	1.06 (1.02, 1.11)	6.8 × 10^−3^	93,052	1.07 (1.02, 1.12)	2.79 × 10^−3^	1.07 (1.03, 1.10)	5.76 × 10^−5^*	7.82 × 10^−1^	EU
20	rs6017317	*HNF4A*	G/T	0.426	0.394	0.177	1.05 (1.01, 1.08)	9.9 × 10^−3^	93,123	1.09 (1.07, 1.12)	1.12 × 10^−11^	1.08 (1.06, 1.10)	2.37 × 10^−15^*	3.89 × 10^−2^	EA
23	rs5945326	*DUSP9*	A/G	0.605	0.607	0.785	1.11 (1.07, 1.15)	4.1 × 10^−7^*	92,979	-	-	-	-	-	EU

OR for diabetes is for combined prevalent and incident cases per allele adjusting for baseline age, sex and regional centre

^a^Meta-analyses were performed using inverse-variance weights under a fixed model

^b^Population from which the loci was first reported in GWAS studies

^c^ *p* for heterogeneity of ORs between CKB and AGEN-T2D; proxy SNPs in HapMap-CHB + JPT:

^d^rs7903146(*r* ^2^ = 1)

^e^rs2334499 (*r* ^2^ = 1)

^f^rs391300 (*r* ^2^ = 0.92)

*p < 0.05 after adjustment for multiple comparisons using the Holm–Bonferroni procedure

CHR, chromosome; EA, East Asians; EU, Europeans; R/A, risk/alternative allele; SA, South Asians

### Meta-analysis of CKB and AGEN-T2D studies

Meta-analysis combining the results of the present study with those from AGEN-T2D [14], providing a total of 32,188 cases and 115,633 controls, further improved concordance of effect estimates: after excluding variants identified in AGEN-T2D or its contributing cohorts, 37 of 40 variants were directionally consistent with European populations (binomial test *p* = 9.73 × 10^−9^). Ten variants identified in GWAS studies of Europeans, plus nine variants reported in East Asian GWAS studies, were genome-wide significant (Table 2, ESM Tables 7, 8). With the exception of *ZFAND3*, we found no heterogeneity for the associations at these loci across CKB and AGEN-T2D (Table 2).

### Consistency of effect sizes between East Asians and Europeans

**Fig. 2:**
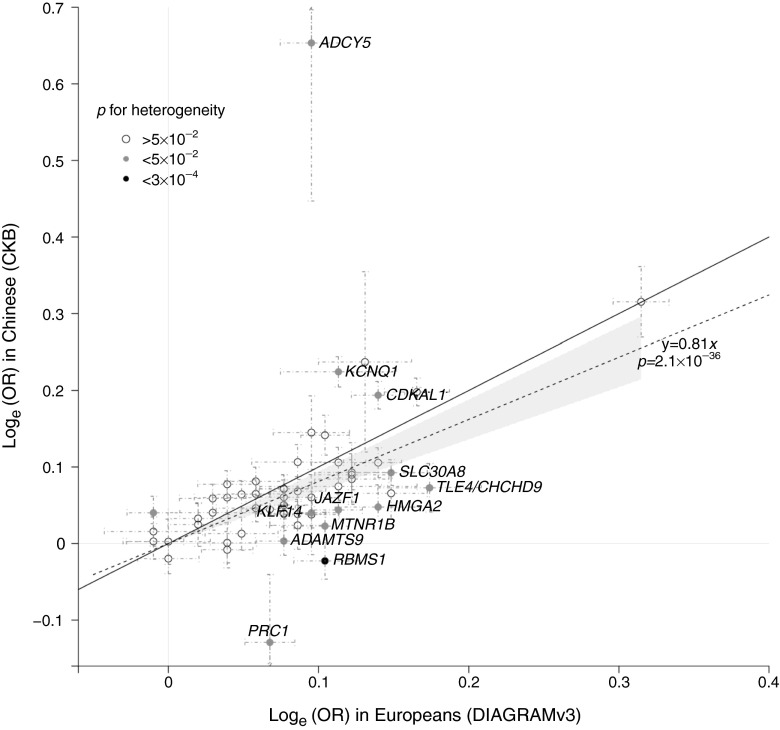
Comparison estimates of effect size (log_e_ (OR)) between Chinese and Europeans. The solid line represents the line of equality, and the result of regression is shown as a broken line with 95% CI

Allelic ORs estimated in CKB were highly correlated with those from Europeans (Fig. 2, *r* = 0.81, *p* = 2.1 × 10^−36^). However, there was a clear trend towards lower effect sizes in this population-based study of Chinese than in the predominantly case–control samples of European descent included in GWAS discovery studies, with a mean proportional reduction in log_e_ OR of 19% (95% CI 6, 32; *p* = 4.8 × 10^−3^). A very similar reduction in effect size was observed when comparing CKB and AGEN-T2D, which also comprised predominantly case–control cohorts (22%; *p* = 3.4 × 10^−3^, ESM Fig. 2a). Effect sizes were also strongly correlated when comparing the meta-analysis of CKB and AGEN-T2D with Europeans (*r* = 0.85, *p* = 7.6 × 10^−37^; proportional reduction of 15%, *p* = 0.026) (ESM Fig. 2b).

Heterogeneity in effect size was observed at *RBMS1* rs7593730 and *GCC1*-*PAX4* rs6467136 (*p* < 10^–4^) (ESM Table 9), whose associations with diabetes were significant only in Europeans and East Asians, respectively. This potentially reflects the different LD patterns between East Asians and Europeans at these loci (*p* < 0.0002 for both, ESM Table 10). A further large difference in estimated effect size between CKB (or AGEN-T2D + CKB) and Europeans, for *ADCY5* rs11708067 (OR [95% CI]: 1.92 [1.28, 2.88] vs 1.10 [1.06, 1.15]), likely reflects low power and uncertainty in effect size in CKB: neither the difference nor the diabetes association itself was significant after correction for multiple testing. This SNP shows large differences in MAF (0.003 and 0.217 in Chinese and Europeans, respectively). In general, however, risk allele frequencies were similar in CKB and Europeans (CEU) (ESM Fig. 3, *r* = 0.62, *p* = 2.9 × 10^−7^).

### GRSs and type 2 diabetes risk prediction

**Fig. 3:**
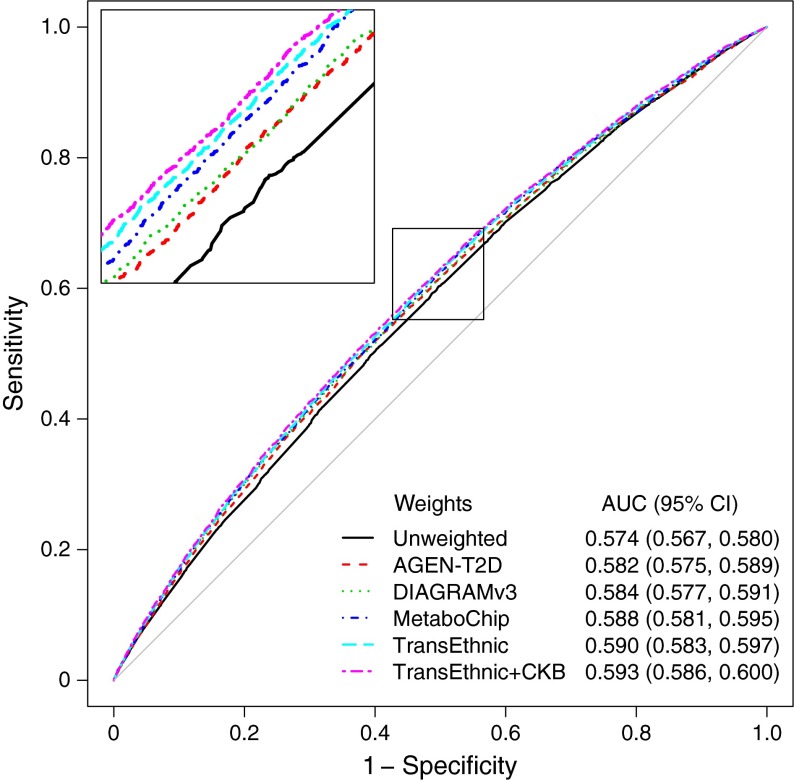
ROC curves for type 2 diabetes GRS-Ts predicting type 2 diabetes in CKB

ROC analysis to assess prediction of diabetes in CKB by GRS-T based on 52 type 2 diabetes risk variants genotyped in the majority of samples showed that, compared with the unweighted risk score (C statistic [95% CI]: 0.574 [0.567, 0.580]), there were significant improvements in discrimination when using risk scores based on weights from previous meta-analyses, TransEthnic in particular (0.590 [0.583, 0.597], *p* = 3.6 × 10^−20^, TransEthnic vs unweighted). There was a further small but significant improvement in diabetes prediction by GRS-T using weights from a meta-analysis including CKB (TransEthnic + CKB) (0.593 [0.586, 0.600]; *p* = 3.0 × 10^−12^, TransEthnic + CKB vs TransEthnic) (Fig. 3 and ESM Table 11). Although somewhat reduced, there remained an improvement following 1000-fold cross-validation to minimise ‘over-fitting’ (0.591 [0.584, 0.598], *p* = 1.8 × 10^−3^). Thus, in terms of diabetes prediction/discrimination this TransEthnic + CKB meta-analysis (ESM Tables 2, 11) provides the best-performing currently available estimates of effect size for these type 2 diabetes associated SNPs.

As expected, both unweighted and weighted GRS robustly associated with risk of diabetes (ESM Table 12). Individuals in the highest quartile of GRS-T had an OR of 2.34 (2.25, 2.45) compared with the lowest quartile. Note that, for this and subsequent analyses, we present the results of analyses employing TransEthnic-weighted GRSs, which represent the best-performing external weights—i.e. which avoid potential over-fitting and, therefore, do not require unnecessarily complex cross-validation analyses. Conclusions were not materially affected by using unweighted risk scores or other externally weighted scores (ESM Table 12).

To investigate the genetic contribution to diabetes related to beta cell dysfunction or IR, two separate GRSs (GRS-BC and GRS-IR) were calculated based on 25 variants predominantly associated with beta cell dysfunction and seven variants with IR, respectively. Assessments of the proportion of variances in HOMA-B and HOMA-IR explained by these GRSs confirmed that they successfully targeted the phenotype of interest (ESM Table 13). We identified associations of both GRS-BC and GRS-IR with diabetes (ESM Table 12). The ORs for diabetes were 2.17 (2.08, 2.26) and 1.19 (1.14, 1.25) when comparing the extreme quartiles of TransEthnic-weighted GRS-BC and GRS-IR, respectively (*p* for trend = 4.82 × 10^−111^ and 1.68 × 10^−7^) (ESM Fig. 4).

### Effect modification by adiposity

Stratified analyses were performed to investigate the possible modifying effects of adiposity on the associations of GRSs with diabetes. Both GRS-T and GRS-BC showed significant interactions with strata for each of BMI, WC, WHR and PBF (*p*_interaction_ < 1 × 10^−4^) (Table 3, ESM Table 15). In each case, per unit GRS score effects were greater in participants who were leaner. We also observed a strong inverse association of GRS-BC with BMI, WC and PBF, but not with WHR (ESM Table 15). In contrast, we found no evidence of interaction between GRS-IR and any of BMI, WC, WHR or PBF (*p*_interaction_ ≥ 0.11).

**Table 3:** Association of GRSs with type 2 diabetes risk in CKB, overall and by levels of adiposity

Characteristic	Stratum	Cases/controls	GRS-T			GRS-BC			GRS-IR		
			OR (95% CI)	*p* value	*p*_interaction_	OR (95% CI)	*p* value	*p*_interaction_	OR (95% CI)	*p* value	*p*_interaction_
BMI^a^											
	Normal weight	1,978/39,862	1.10 (1.09, 1.11)	2.28 × 10^−72^		1.13 (1.11, 1.15)	3.52 × 10^−63^		1.06 (1.03, 1.11)	8.86 × 10^−4^	
	Overweight	3,386/35,711	1.08 (1.07, 1.09)	5.45 × 10^−78^		1.10 (1.09, 1.11)	4.46 × 10^−61^		1.06 (1.03, 1.09)	7.39 × 10^−5^	
	Obese	1,745/10,438	1.06 (1.05, 1.07)	7.07 × 10^−22^	1.45 × 10^−5^	1.08 (1.06, 1.10)	3.15 × 10^−20^	8.86 × 10^−5^	1.04 (1.00, 1.08)	6.29 × 10^−2^	0.37
WC^b^											
	Low	1,067/30,046	1.11 (1.09, 1.12)	8.74 × 10^−51^		1.15 (1.13, 1.17)	5.29 × 10^−44^		1.09 (1.03, 1.14)	1.36 × 10^−3^	
	Medium	2,017/29,075	1.08 (1.07, 1.09)	4.26 × 10^−52^		1.10 (1.08, 1.12)	1.88 × 10^−39^		1.07 (1.03, 1.11)	3.44 × 10^−4^	
	High	4,025/26,890	1.07 (1.06, 1.07)	1.62 × 10^−64^	4.46 × 10^−6^	1.09 (1.08, 1.10)	7.35 × 10^−57^	7.03 × 10^−5^	1.04 (1.01, 1.07)	2.52 × 10^−3^	0.11
WHR^c^											
	Low	1,016/30,035	1.11 (1.10, 1.13)	1.29 × 10^−50^		1.14 (1.12, 1.17)	4.26 × 10^−41^		1.11 (1.06, 1.17)	4.63 × 10^−5^	
	Medium	2,370/32,074	1.08 (1.07, 1.09)	2.70 × 10^−59^		1.11 (1.09, 1.12)	2.00 × 10^−52^		1.04 (1.00, 1.07)	3.29 × 10^−2^	
	High	3,723/23,902	1.06 (1.06, 1.07)	4.16 × 10^−55^	8.66 × 10^−7^	1.08 (1.07, 1.09)	4.06 × 10^−45^	3.59 × 10^−6^	1.05 (1.02, 1.08)	8.93 × 10^−4^	0.15
PBF^d^											
	Low	1,392/29,852	1.10 (1.09, 1.12)	1.59 × 10^−57^		1.13 (1.11, 1.15)	3.74 × 10^−47^		1.06 (1.01, 1.11)	1.02 × 10^−2^	
	Medium	2,180/28,727	1.08 (1.07, 1.09)	6.04 × 10^−53^		1.11 (1.09, 1.12)	2.38 × 10^−45^		1.05 (1.01, 1.09)	6.55 × 10^−3^	
	High	3,533/27,369	1.07 (1.06, 1.08)	8.19 × 10^−58^	1.97 × 10^−5^	1.09 (1.07, 1.10)	1.68 × 10^−47^	8.74 × 10^−5^	1.07 (1.04, 1.10)	5.93 × 10^−6^	0.72
Overall											
		7,109/86,022	1.08 (1.07, 1.08)	4.63 × 10^−155^		1.10 (1.09, 1.11)	2.28 × 10^−126^		1.06 (1.04, 1.08)	7.05 × 10^−8^	

ORs are the effect sizes of each additional point of the TransEthnic weighted GRSs, which corresponds to one additional risk allele

^a^BMI strata were defined according to Asian criteria proposed by WHO (Normal weight, <23 kg/m^2^; Overweight, 23–27.5 kg/m^2^; Obese, ≥27.5 kg/m^2^)

^b^Sex-specific tertiles were used to define WC strata (Low: male <76.9 cm, female <74.4 cm; Medium: male ≥76.9–86.1 cm, female ≥74.4–82.8 cm; High: male ≥86.1 cm, female ≥82.8 cm)

^c^WHR strata (Low: male <0.88, female <0.84; Medium: male ≥0.88–0.94, female ≥0.84–0.91; High: male ≥0.94, female ≥0.91)

^d^PBF (Low: male <18.8%, female <28.8%; Medium: male ≥18.8–24.5%, female ≥28.8–34.9%; High: male ≥24.5%, female ≥34.9%)

## Discussion

We tested associations of 56 variants with risk of diabetes in a large-scale population-based study of Chinese adults. The effect sizes for the majority of diabetes loci were broadly similar between Chinese and European populations. However, there was an overall tendency towards lower effect sizes in our unselected population, likely the consequence of reduced bias (spectrum bias and ‘winner’s curse’). Similar reductions in effect size were observed when comparing with previous data from East Asians, so this is unlikely to be a reflection of differences in patterns of LD.

Improved estimates of SNP effect sizes enabled construction of more accurate weighted GRS for disease prediction. Although GRSs alone remain relatively poor predictors of diabetes risk compared with traditional risk models, the increasing numbers of associated SNPs nevertheless afford improvements for risk prediction [31]. Optimally, integration of genotyping data into type 2 diabetes risk prediction models requires reliable, unbiased, population-specific estimates of the effect of risk variants. Most current effect size estimates have been derived from gene discovery studies largely involving case–control samples and may suffer from ‘winner’s curse’ and disease spectrum bias, the latter not being overcome by ever-larger non-population-based cohort studies. Re-estimation in population-based cohort studies of the effects of GWAS-identified loci limits such biases [32, 33]. Thus, the results from this study can be extrapolated to the Chinese general population and used for inclusion of genetic data in type 2 diabetes risk prediction models.

Previous studies have reported that the majority of common variants are shared across different ethnic groups [24, 30, 47, 48]. We have provided further evidence for shared genetic architecture of type 2 diabetes between East Asian and European populations. Further meta-analysis of CKB with published data from AGEN-T2D improved our statistical power to replicate associations originally reported in other ancestries: the number of variants that achieved genome-wide significance was increased from four to ten, and eight additional SNPs showed significant association after multiple-testing correction (ESM Tables 7, 8).

There are several potential reasons for failure to replicate variants or inconsistencies of effect sizes. First, differences in allele frequency between the original discovery population and the replicating studies in other ethnic groups may affect power for replication. Second, as effect sizes of more-recently identified type 2 diabetes loci become smaller (facilitated by ever-larger sample sizes for discovery), a correspondingly larger sample size is needed for replication. Thus, 19 out of the 26 SNPs with the largest effect sizes (OR ≥ 1.08) but none of the remainder reached genome-wide significance in the CKB-AGEN-T2D meta-analysis. Third, differing patterns of LD may mean that genotyped SNPs are less effective proxies for the underlying causal variant in East Asians than in Europeans: at most loci, the SNPs investigated in the present study were identified in European studies. However, this is likely to apply to only a small subset of loci since common-variant associations map to common haplotypes that are broadly shared between Europeans and East Asians [24, 30, 48]. Thus, at loci where there was apparent non-concordance of effects (e.g. *RBMS1* rs7593730 and *GCC1-PAX4* rs6467136), it remains possible that the same causal variant is present in the two populations and has similar effects. Fourth, these may represent rare instances of ethnic differences in the occurrence of particular causal variants. This may be the case in Europeans for *GCC1-PAX4* rs6467136, for which locus there are no SNPs with a type 2 diabetes association at even *p* < 10^−3^ within 500 kb [15]. This may be elucidated by future fine-mapping and sequencing studies [30].

We further investigated the genetic influence on disease risk by stratifying on genetic variants related primarily to either beta cell function or insulin sensitivity. While variants identified in early GWAS are mainly implicated in beta cell function, more recent studies have identified several variants with a primary impact on IR [15, 40, 41]. Stratification of type 2 diabetes cases according to the separate contributions of genetic effects on beta cell function and IR has the potential to be informative for so-called ‘precision medicine’.

Consistent with previous reports [10, 34, 49], we identified interactions of GRS with measures of adiposity: GRS-BC had a larger effect size on diabetes among individuals with lower BMI, WC, WHR or PBF. Since we observed a strong inverse association of GRS-BC with BMI, WC and PBF (ESM Table 15), it remains possible that the observed interactions with these measures of adiposity are an artefact of the dual effects of GRS-BC on both diabetes risk and adiposity, for instance due to ‘collider bias’ whereby analyses stratified by a potential mediator can induce new relationships and introduce confounding. However, this is not relevant for the interaction with WHR, which displayed a similar magnitude of interaction despite not showing association with GRS-BC. Therefore, we conclude that these observed effects of adiposity of GRS-BC effect size are likely to be genuine. By contrast, we found no evidence for interactions between GRS-IR and adiposity measures, which may reflect limited power (owing to GRS-IR comprising one-third the number of SNPs compared with GRS-BC and being less strongly associated with diabetes), but our findings are in line with previous findings that IR scores are associated with incident type 2 diabetes independent of body size [50].

In conclusion, we report estimates, expected to be largely free of ‘winner’s curse’ and spectrum bias, of the effect sizes of diabetes risk variants in a general population cohort of Chinese adults. We thereby identify the extent to which previous GWAS based on predominantly case–control studies are affected by these biases. In addition to their utility for improvements in type 2 diabetes risk prediction, these more accurate effect size estimates promise to be a powerful resource for future Mendelian randomisation studies in Chinese cohorts.

## Electronic supplementary material

Below is the link to the electronic supplementary material.

China Kadoorie Biobank Collaborative Group (PDF 84 kb)

ESM Table 1 (PDF 79 kb)

ESM Table 2 (PDF 11 kb)

ESM Table 3 (PDF 10 kb)

ESM Table 4 (PDF 10 kb)

ESM Table 5 (PDF 35 kb)

ESM Table 6 (PDF 36 kb)

ESM Table 7 (PDF 9 kb)

ESM Table 8 (PDF 65 kb)

ESM Table 9 (PDF 131 kb)

ESM Table 10 (PDF 100 kb)

ESM Table 11 (PDF 31 kb)

ESM Table 12 (PDF 32 kb)

ESM Table 13 (PDF 5 kb)

ESM Table 14 (PDF 77 kb)

ESM Table 15 (PDF 36 kb)

ESM Fig. 1 (PDF 216 kb)

ESM Fig. 2 (PDF 191 kb)

ESM Fig. 3 (PDF 147 kb)

ESM Fig. 4 (PDF 120 kb)

## Abbreviations

AGEN-T2D: Asian Genetic Epidemiology Network-Type 2 Diabetes Consortium
CKB: China Kadoorie Biobank
GRS: Genetic risk score
GRS-BC: Beta cell function related genetic risk score
GRS-IR: Insulin resistance related genetic risk score
GRS-T: Overall genetic risk score
GWAS: Genome-wide association studies
HOMA-B: HOMA of beta cell function
IR: Insulin resistance
LD: Linkage disequilibrium
MAF: Minor allele frequency
PBF: Percentage body fat
QC: Quality control
ROC: Receiver operating characteristic
SNP: Single nucleotide polymorphism
TransEthnic: Trans-ethnic type 2 diabetes GWAS meta-analysis
TransEthnic + CKB: Combined meta-analysis of the CKB and trans-ethnic GWAS studies
WC: Waist circumference
