# Supplementary material for: Evaluation of type 2 diabetes genetic risk variants in Chinese adults: findings from 93,000 individuals from the China Kadoorie Biobank
Source: Diabetologia. 2016 Apr 6;59:1446–57. doi: 10.1007/s00125-016-3920-9 (PMC4901105; doi:10.1007/s00125-016-3920-9)
Supplement: Supplementary file 1 — (PDF 84 kb) [file 125_2016_3920_MOESM1_ESM.pdf]

International Steering Committee: Junshi Chen, Zhengming Chen (PI), Rory Collins, Liming Li (PI), Richard Peto.

International Co-ordinating Centre, Oxford: Daniel Avery, Derrick Bennett, Yumei Chang, Yiping Chen, Zhengming Chen, Robert Clarke, Huaidong Du, Xuejuan Fan, Simon Gilbert, Alex Hacker, Michael Holmes, Andri Iona, Christiana Kartsonaki; Rene Kerosi, Ling Kong, Om Kurmi, Garry Lancaster, Sarah Lewington, John McDonnell, Winnie Mei, Iona Millwood, Qunhua Nie, Jayakrishnan Radhakrishnan, Sajjad Rafiq, Paul Ryder, Sam Sansome, Dan Schmidt, Paul Sherliker, Rajani Sohoni, Iain Turnbull, Robin Walters, Jenny Wang, Lin Wang, Ling Yang, Xiaoming Yang.

National Co-ordinating Centre, Beijing: Zheng Bian, Ge Chen, Yu Guo, Bingyang Han, Can Hou, Jun Lv, Pei Pei, Shuzhen Qu, Yunlong Tan, Canqing Yu, Huiyan Zhou.

Ten Regional Co-ordinating Centres: Qingdao Qingdao CDC: Zengchang Pang, Ruqin Gao, Shaojie Wang, Yongmei Liu, Ranran Du, Yajing Zang, Liang Cheng, Xiaocao Tian, Hua Zhang. Licang CDC: Silu Lv, Junzheng Wang, Wei Hou. **Heilongjiang** Provincial CDC: Jiyuan Yin, Ge Jiang, Shumei Liu, Zhigang Pang, Xue Zhou. Nangang CDC: Liqiu Yang, Hui He, Bo Yu, Yanjie Li, Huaiyi Mu, Qinai Xu, Meiling Dou, Jiaojiao Ren. **Hainan** Provincial CDC: Jianwei Du, Shanqing Wang, Ximin Hu, Hongmei Wang, Jinyan Chen, Yan Fu, Zhenwang Fu, Xiaohuan Wang, Hua Dong. Meilan CDC: Min Weng, Xiangyang Zheng, Yijun Li, Huimei Li, Chenglong Li. **Jiangsu** Provincial CDC: Ming Wu, Jinyi Zhou, Ran Tao, Jie Yang. Suzhou CDC: Jie Shen, Yihe Hu, Yan Lu, Yan Gao, Liangcai Ma, Renxian Zhou, Aiyu Tang, Shuo Zhang, Jianrong Jin. **Guangxi** Provincial CDC: Zhenzhu Tang, Naying Chen, Ying Huang. Liuzhou CDC: Mingqiang Li, Jinhuai Meng, Rong Pan, Qilian Jiang, Jingxin Qing, Weiyuan Zhang, Yun Liu, Liuping Wei, Liyuan Zhou, Ningyu Chen, Jun Yang, Hairong Guan. **Sichuan** Provincial CDC: Xianping Wu, Ningmei Zhang, Xiaofang Chen, Xuefeng Tang. Pengzhou CDC: Guojin Luo, Jianguo Li, Xiaofang Chen, Jian Wang, Jiaqiu Liu, Qiang Sun. **Gansu** Provincial CDC: Pengfei Ge, Xiaolan Ren, Caixia Dong. Maiji CDC: Hui Zhang, Enke Mao, Xiaoping Wang, Tao Wang. **Henan** Provincial CDC: Guohua Liu, Baoyu Zhu, Gang Zhou, Shixian Feng, Liang Chang, Lei Fan. Huixian CDC: Yulian Gao, Tianyou He, Li Jiang, Huarong Sun, Pan He, Chen Hu, Qiannan Lv, Xukui Zhang. **Zhejiang** Provincial CDC: Min Yu, Ruying Hu, Le Fang, Hao Wang. Tongxiang CDC: Yijian Qian, Chunmei Wang, Kaixue Xie, Lingli Chen, Yaxing Pan, Dongxia Pan. **Hunan** Provincial CDC: Yuelong Huang, Biyun Chen, Donghui Jin, Huilin Liu, Zhongxi Fu, Qiaohua Xu. Liuyang CDC: Xin Xu, Youping Xiong, Weifang Jia, Xianzhi Li, Libo Zhang, Zhe Qiu.

BGI-Shenzhen: Nie Chao, Wang Jun, Qibin Li, Xiao Liu, Hongcheng Zhou.
