# Supplementary material for: Evaluation of type 2 diabetes genetic risk variants in Chinese adults: findings from 93,000 individuals from the China Kadoorie Biobank
Source: Diabetologia. 2016 Apr 6;59:1446–57. doi: 10.1007/s00125-016-3920-9 (PMC4901105; doi:10.1007/s00125-016-3920-9)
Supplement: Supplementary file 2 — (PDF 79 kb) [file 125_2016_3920_MOESM2_ESM.pdf]

ESM Table 1 Genetic and physiologic evidences for classification of the T2D risk variants

Chr	Position	SNP	Nearby genes	Sample Size	Risk Allele	Other allele	GWAS Study Reported OR	Classificantion	Classificantion Criteria	Risk Allele Frequency
1	214159256	rs340874	*PROX1*	93,106	C	T	1.07 [1.05–1.09]	Beta Cell Function	BC(i, iii)	0.562
2	43732823	rs7578597	*THADA*	93,131	T	C	1.15 [1.10-1.20]	Beta Cell Function	BC(i, iii)	0.876
3	123065778	rs11708067	*ADCY5*	93,127	A	G	1.12 [1.09–1.15]	Beta Cell Function	BC(i)	0.774
3	185529080	rs1470579	*IGF2BP2*	93,108	C	A	1.14 [1.09-1.19]	Beta Cell Function	BC(i)	0.296
3	186666461	rs16861329	*ST64GAL1*	93,098	C	T	1.09 [1.06-1.12]	Beta Cell Function	BC(ii)	0.883
4	1309901	rs6815464	*MAEA*	93,082	C	G	1.13 [1.10–1.16]	Beta Cell Function	BC(ii)	-
		rs6819243 (proxy of rs6815464, r2=0.966)			T	C				0.033
4	6292915	rs10010131	[j] *WFS1*	45,198	G	A	1.11 [1.05-1.16]	Beta Cell Function	BC(iv)	0.677
6	20661250	rs7754840	*CDKAL1*	93,130	C	G	1.12 [1.08-1.16]	Beta Cell Function	BC(ii, iii)	0.336
7	15064309	rs2191349	*DGKB*	74,375	T	G	1.06 [1.04–1.08]	Beta Cell Function	BC(i, iii)	0.467
7	44235668	rs4607517	*GCK*	93,113	A	G	1.07 [1.05–1.10]	Beta Cell Function	BC(i, ii)	0.195
7	127164958	rs6467136	*GCC1-PAX4*	93,018	G	A	1.11 [1.07–1.14]	Beta Cell Function	BC(iv)	0.5
8	118184783	rs13266634	*SLC30A8*	92,535	C	T	1.15 [1.12-1.19]	Beta Cell Function	BCi,BCii,BCiii	0.761
9	4287466	rs7041847	*GLIS3*	92,708	A	G	1.10 [1.07–1.13]	Beta Cell Function	BC(i)	0.553
9	22134094	rs10811661	*CDKN2A/B*	93,062	T	C	1.20 [1.12-1.28]	Beta Cell Function	BC(ii, iii)	0.801
10	94462882	rs1111875	*HHEX/IDE*	93,093	C	T	1.13 [1.09–1.17]	Beta Cell Function	BC(ii, iii)	0.584
10	114754088	rs7901695	*TCF7L2*	92,365	C	T	1.41 [1.26–1.58]	Beta Cell Function	BC(i, ii, iii)	0.281
11	1702055	rs4752781	[j] *DUSP8/INS*	92,932	T	A	1.35	Beta Cell Function	BC(ii)	0.465
11	2839751	rs2237892	*KCNQ1*	92,991	C	T	1.40 [1.34-1.47]	Beta Cell Function	BC(i)	0.925
11	17408630	rs5215	*KCNJ11*	93,120	C	T	1.14 [1.10-1.19]	Beta Cell Function	BC(ii)	0.398
11	72433098	rs1552224	*ARAP1*	93,130	A	C	1.14 [1.11-1.17]	Beta Cell Function	BCi,BCii,BCiii	0.867
11	92708710	rs10830963	*MTNR1B*	93,107	G	C	1.09 [1.06-1.12]	Beta Cell Function	BC(i, ii)	0.3
13	80717156	rs1359790	*SPRY2*	93,096	G	A	1.15 [1.10-1.20]	Beta Cell Function	BC(i)	0.727
15	62396389	rs7172432	*VPS13C*	93,095	A	G	1.11 [1.08-1.14]	Beta Cell Function	BC(i, ii)	0.58
15	90374257	rs2028299	*AP3S2*	93,115	C	A	1.10 [1.07-1.13]	Beta Cell Function	BC(ii)	0.259
15	91521337	rs8042680	*PRC1*	93,128	A	C	1.07 [1.05-1.09]	Beta Cell Function	BC(ii)	0.261
17	36098040	rs4430796	*HNF1B*	93,089	G	A	1.14 [1.08-1.20]	Beta Cell Function	BC(i, ii)	0.509
20	42946966	rs6017317	*HNF4A*	93,123	G	T	1.09 [1.07–1.12]	Beta Cell Function	BC(ii, iv)	0.183
2	27741237	rs780094	*GCKR*	93,120	C	T	1.06 [1.04–1.08]	Insulin Resistance	IR(i, ii, iii, iv)	0.606
2	161171454	rs7593730	[k] *RBMS1*	93,129	C	T	1.11 [1.08-1.16]	Insulin Resistance	IR(i)	0.832
2	165501849	rs3923113	*GRB14*	93,088	A	C	1.09 [1.06-1.13]	Insulin Resistance	IR(i, iv)	0.593
2	227093745	rs2943641	*IRS1*	93,104	C	T	1.19 [1.13-1.25]	Insulin Resistance	IR(i, iii, iv)	0.633
3	12393125	rs1801282	*PPARG*	93,126	C	G	1.14 [1.08-1.20]	Insulin Resistance	IR(i, iii, iv)	0.903
3	64048297	rs831571	*PSMD6*	93,094	C	T	1.09 [1.06–1.12]	Insulin Resistance	IR(i)	0.774
7	130466854	rs972283	*KLF14*	93,126	G	A	1.07 [1.05-1.10]	Insulin Resistance	IR(i, iii, iv)	0.55
12	66174894	rs1531343	*HMGA2*	92,189	C	G	1.10 [1.07-1.14]	Insulin Resistance	IR(i, ii)	0.115
		rs2261181 (proxy of rs1531343 r2= 0.93)			T	C				0.111
1	120517959	rs10923931	*NOTCH2*	93,125	T	G	1.13 [1.08-1.17]	Unclassified		0.093
2	60584819	rs243021	*BCL11A*	93,125	A	G	1.08 [1.06-1.10]	Unclassified		0.478
3	23198484	rs6780569	*UBE2E2*	93,127	G	A	1.21 [1.14-1.30]	Unclassified		0.907
3	64711904	rs4607103	*ADAMTS9*	93,114	C	T	1.09 [1.06-1.12]	Unclassified		0.81
5	76424949	rs4457053	*ZBED3*	93,124	G	A	1.08 [1.06-1.11]	Unclassified		0.259
6	38106844	rs9470794	*ZFAND3*	93,111	C	T	1.12 [1.08–1.16]	Unclassified		0.122
7	28180556	rs864745	*JAZF1*	93,120	T	C	1.10 [1.07-1.13]	Unclassified		0.487
8	95960511	rs896854	*TP53INP1*	93,127	T	C	1.06 [1.04-1.09]	Unclassified		0.436
9	8879118	rs17584499	*PTPRD*	93,019	T	C	1.57 [1.36-1.82]	Unclassified		0.226
9	81952128	rs13292136	*TLE4/CHCHD9*	93,118	C	T	1.11 [1.07-1.15]	Unclassified		0.933
10	119389891	rs10886471	*GRK5*	84,095	C	T	1.12 [1.08-1.16]	Unclassified		0.478
10	12314997	rs10906115	*CDC123*	93,076	A	G	1.13 [1.08-1.18]	Unclassified		0.642
10	70931474	rs1802295	*VPS26A*	93,048	A	G	1.08 [1.05-1.12]	Unclassified		0.353
12	71663102	rs7961581	*TSPAN8/LGR5*	93,114	C	T	1.09 [1.06-1.12]	Unclassified		0.252
15	38530704	rs7403531	*RASGRP1*	84,075	T	C	1.10 [1.06-1.13]	Unclassified		0.279
15	77747190	rs7178572	*HMG20A*	93,127	G	A	1.09 [1.06-1.12]	Unclassified		0.681
15	80432222	rs11634397	*ZFAND6*	93,115	G	A	1.06 [1.04-1.08]	Unclassified		0.642
16	53820527	rs9939609	*FTO*	93,123	A	T	1.15 [1.09-1.23]	Insulin Resistance		0.46
17	2208899	rs4523957	*SRR*	90,663	T	G	1.28 [1.18-1.39]	Unclassified		0.615
18	57884750	rs12970134	*MC4R*	93,052	A	G	1.08 [1.05–1.11]	Insulin Resistance		0.279
X	152899922	rs5945326	[l] *DUSP9*	92,979	A	G	1.27 [1.18-1.37]	Unclassified		0.793

SNP	HOMA-B[a] Effect Size	HOMA-B[a] p	CIR[b] Effect Size	CIR[b] p	AUC_{Ins}/AUC_{Gluc}[b] Effect Size	AUC_{Ins}/AUC_{Gluc}[b] p	Ins30[b] Effect Size	Ins30[b] p	Ins30adjBMI[b] Effect Size	Ins30adjBMI[b] p	Increm30[b] Effect Size	Increm30[b] p	Dib Effect Size	Dib p	AUC_{Insb}[b] Effect Size	AUC_{Insb}[b] p	CIR adj ISI[b] Effect Size	CIR adj ISI[b] p
rs340874	-0.014	$3.43x10^{-5}$ *	-0.021	$3.13x10^{-1}$	-0.02	$3.72x10^{-1}$	-0.0034	$8.78x10^{-1}$	-0.018	$4.23x10^{-1}$	-0.0014	$9.49x10^{-1}$	-0.013	$5.36x10^{-1}$	-0.017	$4.59x10^{-1}$	-0.031	$1.47x10^{-1}$
rs7578597	-0.023	$6.98x10^{-5}$ *	0.019	$6.05x10^{-1}$	-0.049	$2.41x10^{-1}$	-0.021	$6.02x10^{-1}$	-0.034	$3.98x10^{-1}$	-0.041	$3.06x10^{-1}$	0.018	$6.17x10^{-1}$	-0.053	$1.95x10^{-1}$	0.031	$4.18x10^{-1}$
rs11708067	-0.016	$1.77x10^{-4}$ *	-0.018	$5.00x10^{-1}$	0.023	$4.33x10^{-1}$	0.018	$5.22x10^{-1}$	0.0084	$7.68x10^{-1}$	0.041	$1.48x10^{-1}$	-0.046	$8.75x10^{-2}$	0.054	$6.42x10^{-2}$	-0.029	$3.19x10^{-1}$
rs1470579	-0.011	$1.40x10^{-3}$ *	-0.025	$2.39x10^{-1}$	-0.037	$1.25x10^{-1}$	-0.02	$3.99x10^{-1}$	-0.03	$1.95x10^{-1}$	-0.014	$5.48x10^{-1}$	-0.0073	$7.36x10^{-1}$	-0.036	$1.31x10^{-1}$	-0.028	$2.23x10^{-1}$
rs16861329	0.0016	$7.48x10^{-1}$	-0.028	$4.30x10^{-1}$	-0.041	$3.04x10^{-1}$	-0.058	$1.30x10^{-1}$	-0.048	$2.04x10^{-1}$	-0.09	$1.94x10^{-2}$ *	-0.031	$3.91x10^{-1}$	-0.029	$4.69x10^{-1}$	-0.044	$2.49x10^{-1}$
rs6815464	-	-	-	-	-	-	-	-	-	-	-	-	-	-	-	-	-	-
rs6819243 (proxy)	-0.025	$9.51x10^{-3}$ *	0.044	$4.29x10^{-1}$	-0.071	$2.40x10^{-1}$	0.054	$3.31x10^{-1}$	-0.092	$8.15x10^{-2}$	-0.081	$1.26x10^{-1}$	-0.067	$2.31x10^{-1}$	0.017	$7.74x10^{-1}$	0.056	$3.25x10^{-1}$
rs10010131	-0.0033	$3.18x10^{-1}$	0.021	$3.10x10^{-1}$	0.029	$1.91x10^{-1}$	0.042	$5.51x10^{-2}$	0.035	$1.12x10^{-1}$	0.028	$1.97x10^{-1}$	-0.0096	$6.45x10^{-1}$	0.032	$1.55x10^{-1}$	0.025	$2.59x10^{-1}$
rs7754840	-0.0066	$5.37x10^{-2}$	-0.11	$2.27x10^{-7}$ *	-0.055	$1.94x10^{-2}$ *	-0.062	$6.19x10^{-3}$ *	-0.082	$3.62x10^{-4}$ *	-0.056	$1.32x10^{-2}$ *	-0.099	$3.45x10^{-6}$ *	-0.028	$2.22x10^{-1}$	-0.11	$4.40x10^{-7}$ *
rs2191349	-0.021	$5.04x10^{-11}$ *	-0.018	$3.40x10^{-1}$	-0.0017	$9.38x10^{-1}$	0.0052	$8.02x10^{-1}$	-0.012	$5.72x10^{-1}$	-0.017	$4.26x10^{-1}$	-0.0078	$6.85x10^{-1}$	-0.0025	$9.06x10^{-1}$	-0.01	$6.09x10^{-1}$
rs4607517	-0.025	$4.84x10^{-8}$ *	-0.069	$2.48x10^{-2}$ *	-0.074	$3.07x10^{-2}$ *	-0.032	$3.39x10^{-1}$	-0.055	$9.43x10^{-2}$	-0.027	$4.04x10^{-1}$	-0.027	$3.77x10^{-1}$	-0.052	$1.23x10^{-1}$	-0.062	$5.95x10^{-2}$
rs6467136	0.0006	$8.52x10^{-1}$	-0.018	$3.68x10^{-1}$	-0.0024	$9.14x10^{-1}$	0.0077	$7.17x10^{-1}$	0.0081	$7.08x10^{-1}$	0.019	$3.61x10^{-1}$	-0.03	$1.31x10^{-1}$	0.0056	$7.98x10^{-1}$	-0.036	$8.56x10^{-2}$
rs13266634	-0.016	$2.36x10^{-5}$ *	-0.078	$6.78x10^{-4}$ *	-0.055	$2.74x10^{-2}$ *	-0.041	$8.75x10^{-2}$	-0.07	$3.67x10^{-3}$ *	-0.049	$4.26x10^{-2}$ *	-0.066	$4.37x10^{-3}$ *	-0.023	$3.41x10^{-1}$	-0.07	$4.08x10^{-3}$ *
rs7041847	-0.012	$1.76x10^{-4}$ *	-0.023	$2.49x10^{-1}$	-0.012	$5.75x10^{-1}$	-0.026	$2.24x10^{-1}$	-0.028	$1.94x10^{-1}$	-0.021	$3.19x10^{-1}$	-0.022	$2.74x10^{-1}$	-0.0083	$7.05x10^{-1}$	-0.026	$2.27x10^{-1}$
rs10811661	-0.0085	$5.11x10^{-2}$	-0.067	$1.32x10^{-2}$ *	-0.058	$5.11x10^{-2}$	-0.083	$4.13x10^{-3}$ *	-0.086	$2.99x10^{-3}$ *	-0.059	$4.07x10^{-2}$ *	-0.084	$2.49x10^{-3}$ *	-0.041	$1.70x10^{-1}$	-0.094	$1.20x10^{-3}$ *
rs1111875	-0.0042	$2.03x10^{-1}$	-0.096	$2.45x10^{-6}$ *	-0.053	$1.67x10^{-2}$ *	-0.065	$2.54x10^{-3}$ *	-0.083	$1.22x10^{-4}$ *	-0.073	$6.52x10^{-4}$ *	-0.087	$1.89x10^{-5}$ *	-0.037	$8.67x10^{-2}$	-0.1	$1.75x10^{-6}$ *
rs7901695	-0.018	$3.13x10^{-6}$ *	-0.057	$1.31x10^{-2}$ *	-0.07	$5.37x10^{-3}$ *	-0.05	$4.11x10^{-2}$ *	-0.063	$1.09x10^{-2}$ *	-0.044	$6.97x10^{-2}$	-0.036	$1.24x10^{-1}$	-0.047	$6.18x10^{-2}$	-0.055	$2.40x10^{-2}$ *
rs4752781	-0.0018	$6.05x10^{-1}$	-0.063	$2.44x10^{-3}$ *	-0.05	$2.69x10^{-2}$ *	-0.037	$9.14x10^{-2}$	-0.059	$7.31x10^{-3}$ *	-0.027	$2.17x10^{-1}$	-0.035	$9.13x10^{-2}$	-0.031	$1.62x10^{-1}$	-0.058	$9.05x10^{-3}$ *
rs2237892	-0.02	$5.78x10^{-3}$ *	-0.04	$3.69x10^{-1}$	-0.044	$3.70x10^{-1}$	-0.033	$4.85x10^{-1}$	-0.035	$4.60x10^{-1}$	-0.015	$7.60x10^{-1}$	-0.015	$7.51x10^{-1}$	-0.016	$7.42x10^{-1}$	-0.02	$6.82x10^{-1}$
rs5215	0.0009	$7.80x10^{-1}$	-0.046	$2.45x10^{-2}$ *	-0.061	$6.15x10^{-3}$ *	-0.038	$7.63x10^{-2}$	-0.056	$9.82x10^{-3}$ *	-0.047	$3.00x10^{-2}$ *	-0.03	$1.50x10^{-1}$	-0.045	$3.96x10^{-2}$ *	-0.04	$6.35x10^{-2}$
rs1552224	-0.017	$9.39x10^{-5}$ *	-0.044	$9.66x10^{-2}$	-0.047	$1.03x10^{-1}$	-0.051	$6.70x10^{-2}$	-0.057	$4.26x10^{-2}$ *	-0.027	$3.39x10^{-1}$	-0.018	$4.91x10^{-1}$	-0.025	$3.72x10^{-1}$	-0.017	$5.32x10^{-1}$
rs10830963	-0.039	$8.60x10^{-23}$ *	-0.18	$1.26x10^{-14}$ *	-0.052	$4.60x10^{-2}$ *	-0.076	$2.52x10^{-3}$ *	-0.12	$4.51x10^{-6}$ *	-0.073	$3.46x10^{-3}$ *	-0.19	- *	-0.0068	$7.90x10^{-1}$	-0.19	$1.73x10^{-14}$ *
rs1359790	-0.0088	$1.58x10^{-2}$ *	0.0026	$9.06x10^{-1}$	-0.033	$1.67x10^{-1}$	-0.023	$3.24x10^{-1}$	-0.023	$3.16x10^{-1}$	-0.031	$1.80x10^{-1}$	0.042	$5.30x10^{-2}$	-0.046	$5.38x10^{-2}$	0.023	$3.04x10^{-1}$
rs7172432	-0.011	$1.11x10^{-3}$ *	-0.085	$3.13x10^{-5}$ *	-0.0036	$8.73x10^{-1}$	-0.056	$9.58x10^{-3}$ *	-0.062	$4.50x10^{-3}$ *	-0.054	$1.35x10^{-2}$ *	-0.083	$5.38x10^{-5}$ *	-0.0014	$9.51x10^{-1}$	-0.089	$5.20x10^{-5}$ *
rs2028299	-0.0017	$6.41x10^{-1}$	-0.041	$7.47x10^{-2}$	-0.053	$3.36x10^{-2}$ *	-0.06	$1.40x10^{-2}$ *	-0.05	$4.21x10^{-2}$ *	-0.061	$1.15x10^{-2}$ *	-0.052	$2.47x10^{-2}$ *	-0.038	$1.21x10^{-1}$	-0.052	$3.32x10^{-2}$ *
rs8042680	-0.0019	$5.78x10^{-1}$	-0.044	$3.59x10^{-2}$ *	-0.023	$3.35x10^{-1}$	-0.014	$5.56x10^{-1}$	-0.023	$3.15x10^{-1}$	-0.019	$4.14x10^{-1}$	-0.039	$6.87x10^{-2}$	-0.0073	$7.58x10^{-1}$	-0.042	$6.01x10^{-2}$
rs4430796	-0.0089	$3.95x10^{-2}$ *	-0.065	$3.59x10^{-2}$ *	-0.035	$3.01x10^{-1}$	-0.042	$2.18x10^{-1}$	-0.054	$1.13x10^{-1}$	-0.023	$5.08x10^{-1}$	-0.053	$8.20x10^{-2}$	-0.013	$7.02x10^{-1}$	-0.062	$4.66x10^{-2}$ *
rs6017317	-0.006	$1.49x10^{-1}$	-0.036	$1.59x10^{-1}$	-0.03	$2.81x10^{-1}$	-0.044	$1.01x10^{-1}$	-0.042	$1.21x10^{-1}$	-0.041	$1.27x10^{-1}$	-0.041	$1.08x10^{-1}$	-0.017	$5.35x10^{-1}$	-0.053	$4.69x10^{-2}$ *
rs780094	0.0039	$2.52x10^{-1}$	0.00013	$9.95x10^{-1}$	0.014	$5.45x10^{-1}$	0.011	$6.14x10^{-1}$	0.015	$5.01x10^{-1}$	0.014	$5.38x10^{-1}$	-0.034	$1.03x10^{-1}$	0.021	$3.56x10^{-1}$	-0.012	$5.87x10^{-1}$
rs7593730	0.0011	$7.83x10^{-1}$	0.037	$1.40x10^{-1}$	0.061	$3.20x10^{-2}$ *	0.044	$1.05x10^{-1}$	0.061	$2.65x10^{-2}$ *	0.048	$7.43x10^{-2}$	-0.0038	$8.78x10^{-1}$	0.054	$5.26x10^{-2}$	0.018	$4.88x10^{-1}$
rs3923113	0.0058	$9.63x10^{-2}$	0.017	$4.41x10^{-1}$	0.026	$2.72x10^{-1}$	0.024	$2.93x10^{-1}$	0.026	$2.71x10^{-1}$	0.023	$3.28x10^{-1}$	-0.028	$1.93x10^{-1}$	0.031	$1.87x10^{-1}$	-0.014	$5.44x10^{-1}$
rs2943641	0.0065	$5.75x10^{-2}$	0.026	$2.05x10^{-1}$	0.026	$2.56x10^{-1}$	0.03	$1.66x10^{-1}$	0.035	$1.18x10^{-1}$	0.022	$3.17x10^{-1}$	-0.002	$9.22x10^{-1}$	0.019	$3.87x10^{-1}$	0.0071	$7.40x10^{-1}$
rs1801282	0.0062	$1.81x10^{-1}$	0.012	$6.85x10^{-1}$	0.054	$7.85x10^{-2}$	0.057	$5.72x10^{-2}$	0.05	$9.30x10^{-2}$	0.054	$7.04x10^{-2}$	-0.037	$1.94x10^{-1}$	0.064	$3.57x10^{-2}$ *	-0.011	$7.08x10^{-1}$
rs831571	-0.0012	$7.69x10^{-1}$	0.0082	$7.55x10^{-1}$	0.063	$2.95x10^{-2}$ *	0.039	$1.67x10^{-1}$	0.039	$1.75x10^{-1}$	0.035	$2.08x10^{-1}$	-0.031	$2.43x10^{-1}$	0.057	$4.52x10^{-2}$ *	0.013	$6.50x10^{-1}$
rs972283	0.0039	$2.37x10^{-1}$	-0.0048	$8.13x10^{-1}$	-0.0022	$9.23x10^{-1}$	-0.016	$4.66x10^{-1}$	-0.02	$3.53x10^{-1}$	-0.0014	$9.48x10^{-1}$	-0.036	$7.59x10^{-2}$	0.015	$4.87x10^{-1}$	-0.018	$3.95x10^{-1}$
rs1531343	0.0006	$9.08x10^{-1}$	0.0085	$8.09x10^{-1}$	0.034	$3.87x10^{-1}$	0.014	$7.17x10^{-1}$	0.023	$5.39x10^{-1}$	0.033	$3.82x10^{-1}$	-0.018	$6.16x10^{-1}$	0.045	$2.48x10^{-1}$	0.0015	$9.68x10^{-1}$
rs2261181 (proxy)	0.0029	$6.09x10^{-1}$	0.013	$7.16x10^{-1}$	0.039	$3.29x10^{-1}$	0.03	$4.31x10^{-1}$	0.026	$5.07x10^{-1}$	0.03	$4.31x10^{-1}$	-0.023	$5.14x10^{-1}$	0.049	$2.14x10^{-1}$	-0.0041	$9.11x10^{-1}$
rs10923931	-0.0045	$3.82x10^{-1}$	-0.035	$2.83x10^{-1}$	-0.031	$3.87x10^{-1}$	0.013	$7.02x10^{-1}$	0.00009	$9.98x10^{-1}$	0.0047	$8.94x10^{-1}$	-0.044	$1.82x10^{-1}$	-0.02	$5.82x10^{-1}$	-0.043	$2.10x10^{-1}$
rs243021	-0.0002	$9.60x10^{-1}$	0.03	$1.41x10^{-1}$	0.026	$2.43x10^{-1}$	0.0035	$8.69x10^{-1}$	0.014	$5.22x10^{-1}$	-0.0017	$9.38x10^{-1}$	0.032	$1.17x10^{-1}$	0.019	$3.77x10^{-1}$	0.043	$4.48x10^{-2}$ *
rs6780569	-0.0061	$2.42x10^{-1}$	-0.055	$7.84x10^{-2}$	-0.039	$2.53x10^{-1}$	-0.061	$6.23x10^{-2}$	-0.056	$9.15x10^{-2}$	-0.058	$7.86x10^{-2}$	-0.023	$4.70x10^{-1}$	-0.033	$3.16x10^{-1}$	-0.037	$2.55x10^{-1}$
rs4607103	0.0009	$8.27x10^{-1}$	0.048	$4.23x10^{-2}$ *	0.042	$1.18x10^{-1}$	0.021	$4.12x10^{-1}$	0.032	$2.13x10^{-1}$	0.033	$1.93x10^{-1}$	-0.0031	$8.97x10^{-1}$	0.037	$1.65x10^{-1}$	0.027	$2.83x10^{-1}$
rs4457053	-0.0071	$7.82x10^{-2}$	-0.031	$2.73x10^{-1}$	-0.045	$1.52x10^{-1}$	0.0022	$9.43x10^{-1}$	-0.013	$6.73x10^{-1}$	0.0024	$9.36x10^{-1}$	0.016	$5.64x10^{-1}$	-0.048	$1.21x10^{-1}$	-0.0038	$8.98x10^{-1}$
rs9470794	-0.007	$2.46x10^{-1}$	0.015	$6.70x10^{-1}$	-0.011	$7.90x10^{-1}$	-0.016	$6.92x10^{-1}$	-0.016	$7.03x10^{-1}$	-0.0093	$8.18x10^{-1}$	-0.0062	$8.65x10^{-1}$	-0.024	$5.66x10^{-1}$	-0.02	$6.00x10^{-1}$
rs864745	-0.0035	$2.83x10^{-1}$	-0.00087	$9.65x10^{-1}$	0.026	$2.36x10^{-1}$	0.011	$6.09x10^{-1}$	0.015	$4.96x10^{-1}$	0.03	$1.63x10^{-1}$	-0.03	$1.33x10^{-1}$	0.032	$1.42x10^{-1}$	-0.011	$6.07x10^{-1}$
rs896854	-0.0046	$1.60x10^{-1}$	0.0071	$7.19x10^{-1}$	-0.032	$1.50x10^{-1}$	-0.028	$1.98x10^{-1}$	-0.017	$4.17x10^{-1}$	-0.021	$3.31x10^{-1}$	0.035	$7.84x10^{-2}$	-0.042	$5.49x10^{-2}$	0.019	$3.73x10^{-1}$
rs17584499	0.0071	$1.58x10^{-1}$	0.06	$4.95x10^{-2}$ *	0.024	$4.66x10^{-1}$	0.021	$5.15x10^{-1}$	0.038	$2.27x10^{-1}$	0.035	$2.62x10^{-1}$	0.067	$2.91x10^{-2}$ *	-0.001	$9.75x10^{-1}$	0.061	$6.14x10^{-2}$
rs13292136	0.0003	$9.56x10^{-1}$	-0.038	$2.70x10^{-1}$	-0.046	$2.19x10^{-1}$	-0.032	$3.77x10^{-1}$	-0.031	$4.01x10^{-1}$	-0.023	$5.14x10^{-1}$	-0.037	$2.76x10^{-1}$	-0.033	$3.64x10^{-1}$	-0.044	$2.18x10^{-1}$
rs10886471	0.0032	$3.58x10^{-1}$	-0.013	$5.55x10^{-1}$	-0.016	$4.87x10^{-1}$	-0.0067	$7.69x10^{-1}$	-0.025	$2.72x10^{-1}$	-0.0035	$8.78x10^{-1}$	-0.0085	$6.96x10^{-1}$	-0.01	$6.54x10^{-1}$	0.0028	$9.01x10^{-1}$
rs10906115	-0.0016	$6.45x10^{-1}$	0.016	$4.48x10^{-1}$	-0.0053	$8.16x10^{-1}$	-0.0024	$9.14x10^{-1}$	0.0015	$9.46x10^{-1}$	-0.015	$4.86x10^{-1}$	0.011	$6.05x10^{-1}$	-0.0039	$8.61x10^{-1}$	0.0056	$7.98x10^{-1}$
rs1802295	-0.0004	$9.13x10^{-1}$	0.02	$3.57x10^{-1}$	-0.011	$6.38x10^{-1}$	0.0051	$8.25x10^{-1}$	-0.00089	$9.69x10^{-1}$	-0.014	$5.33x10^{-1}$	0.016	$4.76x10^{-1}$	-0.0048	$8.40x10^{-1}$	0.023	$3.09x10^{-1}$
rs7961581	-0.0014	$7.11x10^{-1}$	-0.026	$2.57x10^{-1}$	0.0036	$8.89x10^{-1}$	-0.013	$6.00x10^{-1}$	-0.013	$6.09x10^{-1}$	0.014	$5.68x10^{-1}$	-0.027	$2.42x10^{-1}$	0.018	$4.76x10^{-1}$	-0.033	$1.84x10^{-1}$
rs7403531	-0.004	$3.30x10^{-1}$	-0.022	$3.69x10^{-1}$	-0.036	$1.81x10^{-1}$	-0.0064	$8.06x10^{-1}$	-0.027	$2.97x10^{-1}$	-0.021	$4.10x10^{-1}$	0.018	$4.77x10^{-1}$	-0.032	$2.33x10^{-1}$	0.0039	$8.83x10^{-1}$
rs7178572	-0.0042	$2.32x10^{-1}$	0.0023	$9.14x10^{-1}$	-0.025	$3.04x10^{-1}$	0.0037	$8.72x10^{-1}$	0.0023	$9.19x10^{-1}$	0.0046	$8.40x10^{-1}$	-0.0085	$6.95x10^{-1}$	-0.0099	$6.76x10^{-1}$	0.0022	$9.24x10^{-1}$
rs11634397	-0.0034	$3.33x10^{-1}$	0.017	$4.48x10^{-1}$	-0.0054	$8.25x10^{-1}$	-0.012	$6.05x10^{-1}$	0.0046	$8.45x10^{-1}$	-0.012	$6.18x10^{-1}$	0.028	$2.27x10^{-1}$	-0.013	$6.00x10^{-1}$	0.019	$4.20x10^{-1}$
rs9939609	0.0075	$2.35x10^{-2}$ *	-0.016	$4.13x10^{-1}$	-0.028	$2.08x10^{-1}$	-0.042	$4.96x10^{-2}$ *	-0.044	$4.48x10^{-2}$ *	-0.054	$1.27x10^{-2}$ *	-0.0012	$9.51x10^{-1}$	-0.032	$1.43x10^{-1}$	-0.015	$4.90x10^{-1}$
rs4523957	0.0009	$7.86x10^{-1}$	-0.0073	$7.29x10^{-1}$	-0.012	$6.22x10^{-1}$	0.0017	$9.40x10^{-1}$	-0.013	$5.83x10^{-1}$	-0.012	$5.85x10^{-1}$	-0.012	$5.68x10^{-1}$	-0.015	$5.34x10^{-1}$	-0.007	$7.55x10^{-1}$
rs12970134	0.0073	$6.30x10^{-2}$	0.014	$5.39x10^{-1}$	0.033	$2.04x10^{-1}$	0.0048	$8.49x10^{-1}$	0.033	$2.01x10^{-1}$	0.01	$6.89x10^{-1}$	0.018	$4.30x10^{-1}$	0.02	$4.44x10^{-1}$	0.014	$5.65x10^{-1}$
rs5945326	-	-	-	-	-	-	-	-	-	-	-	-	-	-	-	-	-	-

SNP	IGI[c] Effect Size	IGI[c] p	AIR[c] Effect Size	AIR[c] p	HOMA-Ira Effect Size	HOMA-Ira p	FIadjBMI[a] Effect Size	FIadjBMI[a] p	FIadjBMIinter[a] Effect Size	FIadjBMIinter[a] p	ISI[b] Effect Size	ISI[b] p	Clusters of Type 2 Diabetes Risk Loci[d]	Lipid Traits[e,f,g,h]	SNPs proposed by Vassy et al [i]
rs340874	-0.028	$6.00x10^{-3}$ *	-0.043	$1.80x10^{-1}$	-0.0042	$3.26x10^{-1}$	-0.0047	$2.55x10^{-2}$	0.0068	$8.08x10^{-1}$	0.012	$5.80x10^{-1}$	Beta Cell Function		β-cell function
rs7578597		-	-	-	-0.0091	$1.89x10^{-1}$	-	-	0.018	$4.91x10^{-3}$ *	0.011	$8.09x10^{-1}$	Beta Cell Function		β-cell function
rs11708067	-0.0089	$4.90x10^{-1}$	-0.013	$7.50x10^{-1}$	0.001	$8.40x10^{-1}$	-0.011	$1.43x10^{-5}$	-0.012	$1.93x10^{-1}$	-0.05	$8.37x10^{-2}$	Beta Cell Function		β-cell function
rs1470579	-0.038	$3.00x10^{-2}$ *	-0.15	$1.40x10^{-3}$ *	-0.0085	$4.72x10^{-2}$ *	-	-	-0.0051	$8.90x10^{-1}$	0.039	$9.04x10^{-2}$	Unclassified		β-cell function
rs16861329	-	-	-	-	0.0025	$6.98x10^{-1}$	-	-	0.00019	$6.56x10^{-1}$	0.022	$5.82x10^{-1}$	-		NA
rs6815464	-	-	-	-	-	-	-	-	-	-	-	-	-		β-cell function
rs6819243 (proxy)	-	-	-	-	-0.0092	$4.44x10^{-1}$	-0.004	$4.92x10^{-1}$	-0.06	$1.31x10^{-1}$	0.034	$5.44x10^{-1}$	-		NA
rs10010131	-	-	-	-	0.0085	$3.47x10^{-2}$ *	-	-	0.04	$6.57x10^{-2}$	-0.048	$2.92x10^{-2}$ *	Unclassified		NA
rs7754840	-	-	-	-	-0.0051	$2.21x10^{-1}$	-	-	-0.0018	$7.31x10^{-2}$	0.022	$3.88x10^{-1}$	Beta Cell Function		β-cell function
rs2191349	-0.028	$5.80x10^{-3}$ *	-0.013	$6.70x10^{-1}$	-0.0015	$6.95x10^{-1}$	-0.001	$6.30x10^{-1}$	0.043	$3.49x10^{-2}$ *	0.024	$2.36x10^{-1}$	Beta Cell Function		β-cell function
rs4607517	-0.036	$3.10x10^{-2}$ *	-0.097	$5.10x10^{-2}$	0.015	$6.32x10^{-3}$ *	-	-	-0.028	$8.03x10^{-2}$	0.038	$2.59x10^{-1}$	Hyperglycemic		β-cell function
rs6467136	-	-	-	-	0.0005	$8.93x10^{-1}$	-	-	-0.02	$4.19x10^{-1}$	-0.028	$2.25x10^{-1}$	-		NA
rs13266634	-	-	-	-	-0.0002	$9.69x10^{-1}$	-0.0044	$5.52x10^{-2}$	0.0091	$3.68x10^{-1}$	0.016	$5.05x10^{-1}$	Beta Cell Function		β-cell function
rs7041847	-	-	-	-	-0.0051	$1.98x10^{-1}$	-	-	-0.001	$4.10x10^{-2}$ *	0.007	$7.71x10^{-1}$	-		β-cell function
rs10811661	-	-	-	-	0.0054	$3.01x10^{-1}$	-0.0065	$1.94x10^{-2}$	0.0049	$7.30x10^{-1}$	0.019	$5.21x10^{-1}$	Beta Cell Function		β-cell function
rs1111875	-	-	-	-	0.0028	$4.87x10^{-1}$	-0.0035	$9.52x10^{-2}$	0.00096	$9.97x10^{-1}$	-0.0031	$8.83x10^{-1}$	Beta Cell Function		β-cell function
rs7901695	-	-	-	-	-0.0077	$8.80x10^{-2}$	-0.011	$4.69x10^{-6}$	0.0077	$1.37x10^{-4}$ *	0.03	$2.76x10^{-1}$	Beta Cell Function		β-cell function
rs4752781	-	-	-	-	-0.0014	$7.36x10^{-1}$	-	-	0.012	$2.79x10^{-1}$	0.04	$6.56x10^{-2}$	Unclassified		NA
rs2237892	-	-	-	-	-0.019	$3.64x10^{-2}$ *	-	-	-0.0044	$3.74x10^{-1}$	0.017	$7.31x10^{-1}$	Unclassified		β-cell function
rs5215	-0.024	$1.50x10^{-1}$	-0.099	$1.80x10^{-2}$ *	-0.0018	$6.55x10^{-1}$	-0.0011	$6.03x10^{-1}$	-0.0046	$7.39x10^{-1}$	0.028	$1.94x10^{-1}$	Unclassified		β-cell function
rs1552224	-0.054	$1.54x10^{-5}$ *	-0.0064	$9.10x10^{-1}$	-0.0092	$8.46x10^{-2}$	-0.0048	$7.51x10^{-2}$	-0.049	$1.30x10^{-2}$ *	0.023	$4.14x10^{-1}$	Proinsulin		β-cell function
rs10830963	-0.083	$1.23x10^{-14}$ *	-0.16	$4.81x10^{-6}$ *	0.0083	$9.07x10^{-2}$	-0.0013	$6.09x10^{-1}$	0.0067	$9.45x10^{-1}$	-0.016	$5.29x10^{-1}$	Hyperglycemic		β-cell function
rs1359790	-	-	-	-	-0.011	$9.70x10^{-3}$ *	-0.0084	$1.62x10^{-4}$	-0.0035	$1.97x10^{-3}$ *	0.066	$3.60x10^{-3}$ *	-		NA
rs7172432	-	-	-	-	-0.0008	$8.48x10^{-1}$	-	-	-0.02	$1.56x10^{-1}$	-0.01	$6.66x10^{-1}$	-		β-cell function
rs2028299	-	-	-	-	-0.0056	$2.09x10^{-1}$	-	-	0.0048	$9.54x10^{-1}$	-0.000012	1	-		NA
rs8042680	-0.016	$1.50x10^{-1}$	0.00053	$9.90x10^{-1}$	0.0033	$4.39x10^{-1}$	-	-	0.021	$4.69x10^{-1}$	-0.026	$2.97x10^{-1}$	Unclassified		NA
rs4430796	-0.044	$6.10x10^{-2}$	-0.021	$6.10x10^{-1}$	-0.011	$5.49x10^{-2}$	-	-	-0.024	$1.04x10^{-2}$ *	0.00072	$9.83x10^{-1}$	Unclassified		NA
rs6017317	-	-	-	-	-0.0069	$1.70x10^{-1}$	-	-	-0.0011	$6.45x10^{-1}$	0.015	$6.13x10^{-1}$	-		NA
rs780094	0.009	$3.80x10^{-1}$	0.034	-	0.02	$7.63x10^{-7}$ *	0.019	$7.37x10^{-20}$	0.00075	$3.32x10^{-10}$ *	-0.058	$1.80x10^{-2}$ *	Insulin Resistance	Tryglycerides[5]	Insulin resistance
rs7593730	-	-	-	-	0.0077	$1.16x10^{-1}$	-	-	0.01	$8.39x10^{-1}$	-0.058	$4.53x10^{-2}$ *	-		NA
rs3923113	-	-	-	-	0.011	$1.28x10^{-2}$ *	-	-	-0.035	$1.43x10^{-10}$ *	-0.063	$5.53x10^{-3}$ *	-	Tryglycerides[6]	Insulin resistance
rs2943641	-	-	-	-	0.0087	$3.59x10^{-2}$ *	-	-	-0.029	$2.71x10^{-12}$ *	-0.041	$5.65x10^{-2}$	Insulin Resistance	Tryglycerides[6]	Insulin resistance
rs1801282	-	-	-	-	0.016	$5.57x10^{-3}$ *	-	-	-0.017	$2.44x10^{-7}$ *	-0.084	$4.25x10^{-3}$ *	Insulin Resistance	Tryglycerides[7]	Insulin resistance
rs831571	-	-	-	-	0.0069	$1.87x10^{-1}$	-	-	0.0089	$7.94x10^{-1}$	-0.067	$2.98x10^{-2}$ *	-		NA
rs972283	-0.002	$8.50x10^{-1}$	0.019	$6.50x10^{-1}$	0.0095	$1.94x10^{-2}$ *	-	-	-0.023	$8.05x10^{-6}$ *	-0.033	$1.66x10^{-1}$	Insulin Resistance	HDL-C, Tryglyce	Insulin resistance
rs1531343	0.03	$1.10x10^{-1}$	-0.12	$1.10x10^{-1}$	0.0094	$1.55x10^{-1}$	-	-	-0.045	$6.62x10^{-2}$	-0.047	$2.06x10^{-1}$	-		Insulin resistance
rs2261181 (proxy)	-	-	-	-	0.013	$4.88x10^{-2}$ *	0.011	$3.42x10^{-3}$	-0.04	$6.54x10^{-2}$	-0.053	$1.51x10^{-1}$	Unclassified		Insulin resistance
rs10923931	-0.012	$4.50x10^{-1}$	-0.083	$2.10x10^{-1}$	0.0011	$8.69x10^{-1}$	-	-	0.0034	$8.01x10^{-1}$	-0.0015	$9.65x10^{-1}$	Unclassified		NA
rs243021	0.018	$9.30x10^{-2}$	0.0037	$9.30x10^{-1}$	0.001	$7.95x10^{-1}$	-	-	0.034	$1.22x10^{-1}$	-0.00017	$9.94x10^{-1}$	Unclassified		NA
rs6780569	-	-	-	-	-0.013	$4.52x10^{-2}$ *	-	-	-0.065	$1.34x10^{-2}$ *	0.046	$1.83x10^{-1}$	-		β-cell function
rs4607103	-	-	-	-	0.0055	$2.35x10^{-1}$	-	-	-0.036	$4.98x10^{-3}$ *	-0.047	$5.96x10^{-2}$	Unclassified		NA
rs4457053	-0.014	$2.10x10^{-1}$	-0.013	$7.70x10^{-1}$	0.0049	$3.10x10^{-1}$	0.0027	$2.80x10^{-1}$	0.022	$4.24x10^{-1}$	0.048	$1.15x10^{-1}$	Unclassified		NA
rs9470794	-	-	-	-	-0.0029	$6.82x10^{-1}$	-	-	-0.037	$4.35x10^{-1}$	-0.011	$8.04x10^{-1}$	-		NA
rs864745	-	-	-	-	-0.0011	$7.71x10^{-1}$	-	-	-0.011	$8.04x10^{-1}$	-0.023	$3.25x10^{-1}$	Unclassified		NA
rs896854	0.0035	$7.30x10^{-1}$	0.03	$4.60x10^{-1}$	0.0003	$9.36x10^{-1}$	-0.0016	$4.23x10^{-1}$	0.0093	$5.74x10^{-1}$	0.056	$1.56x10^{-2}$ *	Unclassified		NA
rs17584499	-	-	-	-	0.0063	$3.05x10^{-1}$	-	-	-0.02	$2.35x10^{-1}$	0.014	$6.53x10^{-1}$	-		NA
rs13292136	-0.025	$1.30x10^{-1}$	0.04	$5.60x10^{-1}$	0.0022	$7.59x10^{-1}$	-	-	0.013	$5.14x10^{-1}$	-0.026	$4.61x10^{-1}$	Unclassified		NA
rs10886471	-	-	-	-	0.003	$4.86x10^{-1}$	-	-	-0.019	$4.42x10^{-1}$	0.0069	$7.64x10^{-1}$	-		NA
rs10906115	-	-	-	-	0.0015	$7.24x10^{-1}$	-0.0038	$8.31x10^{-2}$	-0.013	$3.98x10^{-1}$	0.0045	$8.37x10^{-1}$	-		β-cell function
rs1802295	-	-	-	-	0.0005	$9.12x10^{-1}$	0.00071	$7.46x10^{-1}$	-0.0026	$9.77x10^{-1}$	-0.0069	$7.64x10^{-1}$	-		NA
rs7961581	-	-	-	-	0.0009	$8.45x10^{-1}$	-	-	0.004	$9.16x10^{-1}$	-0.019	$4.91x10^{-1}$	Unclassified		NA
rs7403531	-	-	-	-	0.0005	$9.15x10^{-1}$	-	-	0.0073	$4.65x10^{-1}$	0.029	$3.12x10^{-1}$	-		NA
rs7178572	-	-	-	-	-0.0014	$7.41x10^{-1}$	-0.00095	$6.63x10^{-1}$	-0.013	$7.50x10^{-1}$	-0.006	$8.11x10^{-1}$	-		NA
rs11634397	-0.0098	$3.40x10^{-1}$	0.075	$9.60x10^{-2}$	-0.0038	$3.78x10^{-1}$	-0.00092	$6.75x10^{-1}$	-0.0018	$4.94x10^{-1}$	0.023	$3.28x10^{-1}$	Unclassified		NA
rs9939609	-	-	-	-	0.015	$3.18x10^{-4}$ *	-	-	0.018	$4.33x10^{-1}$	0.023	$3.31x10^{-1}$	-		Insulin resistance
rs4523957	-	-	-	-	0.0035	$4.03x10^{-1}$	-	-	0.014	$5.02x10^{-2}$	0.0054	$8.09x10^{-1}$	-		NA
rs12970134	-	-	-	-	0.0084	$7.59x10^{-2}$	-	-	0.021	$4.01x10^{-1}$	0.0042	$8.66x10^{-1}$	-		Insulin resistance
rs5945326	-0.037	3.20E-04 *	-0.019	6.30E-01	-	-	-	-	-	-	-	-	Other		NA

Classificantion criterias are as described in the main text (BC (i~iv) andd IR (i~iv)

Reference(PMID): **a**:20081858;**b**:24699409;**c**:4030103;**d**:24296717;**e**:18193043;**f**:22581228;**g**:22885924;**h**:21572415;**i**:24520119

Genetic risk scores was constructed based on different sets of SNPs after excluding those: j genotyped in the minority of the study population; **k** significant effect size heterogeneity;**l** parent-of-origin-specific effect or the location on the X-chromosome;

Abbreviations

HOMA-B: Homeostatic model assessment indices of beta-cell function; **HOMA-IR**: Homeostatic model assessment indices of insulin resistance; **FIadjBMIinter**:Fasting Insulin adjusted for interaction effect with BMI;

FIadjBMI:Fasting Insulin adjusted BMI; **AUCIns/AUCGluc**: Overall insulin response to glucose; **CIR**: Corrected Insulin Response; **Ins3**0: Insulin at 30 min; **Increm30**: Incremental insulin at 30 min; **DI**: Disposition Index;

AUCIns: Area Under the Curve of Insulin levels during OGTT; **Ins30adjBMI**: insulin response to glucose during the first 30 min adjusted for BMI; **IGI:** Insulinogenic Index; **ISI**: Insulin Sensitivity Index;

AIR: Acute Insulin Response
