# Supplementary material for: Evaluation of type 2 diabetes genetic risk variants in Chinese adults: findings from 93,000 individuals from the China Kadoorie Biobank
Source: Diabetologia. 2016 Apr 6;59:1446–57. doi: 10.1007/s00125-016-3920-9 (PMC4901105; doi:10.1007/s00125-016-3920-9)
Supplement: Supplementary file 3 — (PDF 11 kb) [file 125_2016_3920_MOESM3_ESM.pdf]

ESM Table 2 Weights applied to construct genetic risk scores

SNP	Nearby Genes	R/A	**DIAGRAMv3** (Europeans: up to 12,171 cases and 56,862 controls)	**AGEN-T2D** (East Asians: up to 25,079 cases and 29,611 controls)	**MetaboChip** (Europeans: up to 34,840 cases and 114,981 controls)	**TransEthnic** (Europeans: 12,171 cases and 56,862 controls; East Asians: 6,952 cases and 11,865 controls; South Asians: 5,561 cases and 14,458 controls; Mexicans and Mexican Americans: 1,804 cases and 779 controls)	**TransEthnic+CKB** (Europeans: 12,171 cases and 56,862 controls; East Asians: **14,061 cases and 97,887 controls**; South Asians: 5,561 cases and 14,458 controls; Mexicans and Mexican Americans: 1,804 cases and 779 controls)	**Classificantion**
rs340874	*PROX1*	C/T	0.077	0.077	0.064	0.068	0.059	Beta Cell Function
rs7578597	*THADA*	T/C	0.131	-0.073	0.127	0.068	0.073	Beta Cell Function
rs11708067	*ADCY5*	A/G	0.095	0.166	0.107	0.095	0.103	Beta Cell Function
rs1470579	*IGF2BP2*	C/A	0.113	0.140	0.120	0.122	0.117	Beta Cell Function
rs16861329	*ST64GAL1*	C/T	0.030	-0.083	**0.03**[c]	0.086	0.067	Beta Cell Function
rs6815464	*MAEA*	C/G	0.14[a]	0.122	0.071 [b]	0.095	0.080	Beta Cell Function
rs7754840	*CDKAL1*	C/G	0.140	0.166	0.126	0.140	0.159	Beta Cell Function
rs2191349	*DGKB*	T/G	0.068	0.115	0.050	0.086	0.073	Beta Cell Function
rs4607517	*GCK*	A/G	0.049	0.030	0.073	0.030	0.026	Beta Cell Function
rs6467136	*GCC1-PAX4*	G/A	-0.010	0.104	**-0.01** [c]	0.020	0.021	Beta Cell Function
rs13266634	*SLC30A8*	C/T	0.148	0.100	0.126	0.131	0.116	Beta Cell Function
rs7041847	*GLIS3*	A/G	0.049	0.095	0.041	0.058	0.057	Beta Cell Function
rs10811661	*CDKN2A/B*	T/C	0.166	0.109	0.169	0.191	0.194	Beta Cell Function
rs1111875	*HHEX/IDE*	C/T	0.140	0.081	0.105	0.113	0.111	Beta Cell Function
rs7901695	*TCF7L2*	C/T	0.315	0.166	0.296	0.270	0.274	Beta Cell Function
rs2237892	*KCNQ1*	C/T	0.113	0.175	0.147	0.182	0.208	Beta Cell Function
rs5215	*KCNJ11*	C/T	0.077	0.095	0.072	0.086	0.079	Beta Cell Function
rs1552224	*ARAP1*	A/C	0.122	0.148	0.102	0.095	0.095	Beta Cell Function
rs10830963	*MTNR1B*	G/C	0.104	0.000	0.096	0.086	0.054	Beta Cell Function
rs1359790	*SPRY2*	G/A	0.095	0.052	0.074	0.068	0.065	Beta Cell Function
rs7172432	*VPS13C*	A/G	0.058	0.101	**0.058** [c]	0.068	0.066	Beta Cell Function
rs2028299	*AP3S2*	C/A	0.039	0.077	**0.039**	0.068	0.065	Beta Cell Function
rs8042680	*PRC1*	A/C	0.068	0.495	0.065	0.068	0.062	Beta Cell Function
rs4430796	*HNF1B*	G/A	0.122	0.113	0.093	0.095	0.095	Beta Cell Function
rs6017317	*HNF4A*	G/T	0.058	0.086	**0.058** [c]	0.068	0.059	Beta Cell Function
rs780094	*GCKR*	C/T	0.039	0.058	0.060	0.058	0.066	Insulin Resistance
rs3923113	*GRB14*	A/C	0.039	0.030	0.070	0.077	0.051	Insulin Resistance
rs2943641	*IRS1*	C/T	0.077	0.113	0.089	0.086	0.081	Insulin Resistance
rs1801282	*PPARG*	C/G	0.148	0.140	0.123	0.131	0.115	Insulin Resistance
rs972283	*KLF14*	G/A	0.095	-0.010	0.041	0.049	0.045	Insulin Resistance
rs1531343	*HMGA2*	C/G	0.140	0.058	0.118	0.095	0.081	Insulin Resistance
rs831571	*PSMD6*	C/T	0.030	0.090	**0.03** [c]	0.049	0.052	Insulin Resistance
rs10923931	*NOTCH2*	T/G	0.095	0.000	0.079	0.049	0.062	Unclassified
rs243021	*BCL11A*	A/G	0.086	0.049	0.065	0.068	0.068	Unclassified
rs6780569	*UBE2E2*	G/A	0.086	0.156	**0.086** [c]	0.095	0.102	Unclassified
rs4607103	*ADAMTS9*	C/T	0.077	-0.010	0.074	0.039	0.025	Unclassified
rs4457053	*ZBED3*	G/A	0.122	0.000	0.089	0.095	0.094	Unclassified
rs9470794	*ZFAND3*	C/T	-0.010	0.113	**-0.01** [c]	0.058	0.036	Unclassified
rs864745	*JAZF1*	T/C	0.113	0.058	0.095	0.095	0.080	Unclassified
rs896854	*TP53INP1*	T/C	0.086	0.068	0.050	0.077	0.063	Unclassified
rs17584499	*PTPRD*	T/C	0.000	0.086	**0.000** [c]	0.010	0.007	Unclassified
rs13292136	*TLE4/CHCHD9*	C/T	0.174	-0.010	**0.174**	0.104	0.098	Unclassified
rs10906115	*CDC123*	A/G	0.058	0.082	0.033	0.068	0.073	Unclassified
rs1802295	*VPS26A*	T/G	0.020	0.010	0.003	0.049	0.043	Unclassified
rs10886471	*GRK5*	C/T	-0.010	0.058	**-0.01** [c]	0.010	0.008	Unclassified
rs7961581	*TSPAN8/LGR5*	C/T	0.077	0.010	**0.077**	0.058	0.050	Unclassified
rs7403531	*RASGRP1*	T/C	0.020	0.077	**0.02** [c]	0.020	0.024	Unclassified
rs7178572	*HMG20A*	G/A	0.077	0.086	0.072	0.086	0.080	Unclassified
rs11634397	*ZFAND6*	G/A	0.086	0.000	0.047	0.068	0.060	Unclassified
rs9939609	*FTO*	A/T	0.104	0.119	0.113	0.095	0.105	Unclassified
rs4523957	*SRR*	T/G	0.000	0.030	**0.00** [c]	0.020	0.005	Unclassified
rs12970134	*MC4R*	A/G	0.077	0.070	0.075	0.077	0.072	Unclassified

Proxies [a] rs11247991 (r^2=1 with rs6815464 in CHB),[b] rs6819243 (r^2=0.966 withrs6815464 in CHB)
Weights were calculated as natural-log(Odds Ratio)
Each variant’s corresponding derived from:
DIAGRAM: DIAGRAMv3 GWAS meta-analysis
MetaboChip:DIAGRAM Metabochip meta-analysis. [c] Effects of variants not included in metabochip were obtained from DIAGRAMv3 and highlighted in bold
AGEN-T2D: largest T2D-GWAS meta-analysis in East Asian, Asian Genetic Epidemiology Network-Type 2 Diabetes Consortium
TransEthnic: Trans-ethnic Meta analysis of four ethinic groups (East Asians[AGEN-T2D], Europeans [DIAGRAM], South Asians [SAT2D], and Mexicans [MAT2D])
TransEthnic+CKB: Meta-analysis of CKB and Trans-ethnic GWAS (East Asians[AGEN-T2D and CKB], Europeans [DIAGRAM], South Asians [SAT2D], and Mexicans [MAT2D])
