# Supplementary material for: Evaluation of type 2 diabetes genetic risk variants in Chinese adults: findings from 93,000 individuals from the China Kadoorie Biobank
Source: Diabetologia. 2016 Apr 6;59:1446–57. doi: 10.1007/s00125-016-3920-9 (PMC4901105; doi:10.1007/s00125-016-3920-9)
Supplement: Supplementary file 4 — (PDF 10 kb) [file 125_2016_3920_MOESM4_ESM.pdf]

ESM Table 3 Statistical Power

SNP	Nearby Genes	RAF	GWAS Study Reported OR	Sample Size (n)		Power
				Cases	Controls	
rs10923931	*NOTCH2*	0.03152	1.13 [1.08-1.17]	7,109	86,016	0.714
rs340874	*PROX1*	0.3908	1.07 [1.05-1.09]	7,105	86,001	0.967
rs780094	*GCKR*	0.4876	1.06 [1.04-1.08]	7,108	86,012	0.916
rs7578597	*THADA*	0.9926	1.15 [1.10-1.20]	7,109	86,022	0.2627
rs243021	*BCL11A*	0.6703	1.08 [1.06-1.10]	7,107	86,018	0.9846
rs7593730	*RBMS1*	0.8363	1.11 [1.08-1.16]	7,109	86,020	0.9914
rs3923113	*GRB14*	0.8658	1.09 [1.06-1.13]	7,107	85,981	0.9116
rs2943641	*IRS1*	0.9249	1.19 [1.13-1.25]	7,108	85,996	0.999
rs1801282	*PPARG*	0.9462	1.14 [1.08-1.20]	7,109	86,017	0.9072
rs6780569	*UBE2E2*	0.7983	1.21 [1.14-1.30]	7,108	86,019	0.999
rs831571	*PSMD6*	0.6336	1.09 [1.06-1.12]	7,105	85,989	0.9972
rs4607103	*ADAMTS9*	0.6382	1.09 [1.06-1.12]	7,107	86,007	0.9971
rs11708067	*ADCY5*	0.9966	1.12 [1.09-1.15]	7,109	86,018	0.1136
rs1470579	*IGF2BP2*	0.2574	1.14 [1.09-1.19]	7,108	86,000	0.9999
rs16861329	*ST64GAL1*	0.8093	1.09 [1.06-1.12]	7,108	85,990	0.969
rs6815464	*MAEA*	0.5775	1.13 [1.10-1.16]	7,101	85,981	0.9999
rs10010131	*WFS1*	0.9382	1.11 [1.05-1.16]	3,639	41,559	0.5186
rs4457053	*ZBED3*	0.05237	1.08 [1.06-1.11]	7,108	86,016	0.5173
rs7754840	*CDKAL1*	0.4093	1.12 [1.08-1.16]	7,109	86,021	0.9999
rs9470794	*ZFAND3*	0.3161	1.12 [1.08-1.16]	7,109	86,002	0.9999
rs2191349	*DGKB*	0.6543	1.06 [1.04-1.08]	5,621	68,754	0.8034
rs864745	*JAZF1*	0.7661	1.10 [1.07-1.13]	7,107	86,013	0.9954
rs4607517	*GCK*	0.2107	1.07 [1.05-1.10]	7,107	86,006	0.892
rs6467136	*GCC1-PAX4*	0.7836	1.11 [1.07-1.14]	7,102	85,916	0.998
rs972283	*KLF14*	0.7104	1.07 [1.05-1.10]	7,109	86,017	0.9372
rs896854	*TP53INP1*	0.3084	1.06 [1.04-1.09]	7,109	86,018	0.8738
rs13266634	*SLC30A8*	0.5377	1.15 [1.12-1.19]	7,054	85,481	0.9999
rs7041847	*GLIS3*	0.4629	1.10 [1.07-1.13]	7,074	85,634	0.9998
rs17584499	*PTPRD*	0.1011	1.57 [1.36-1.82]	7,100	85,919	0.9999
rs10811661	*CDKN2A/B*	0.5428	1.20 [1.12-1.28]	7,106	85,956	0.9999
rs13292136	*TLE4/CHCHD9*	0.9089	1.11 [1.07-1.15]	7,108	86,010	0.9199
rs10906115	*CDC123*	0.6257	1.13 [1.08-1.18]	7,103	85,973	0.9999
rs1802295	*VPS26A*	0.1093	1.08 [1.05-1.12]	7,106	85,942	0.799
rs1111875	*HHEX/IDE*	0.2791	1.13 [1.09-1.17]	7,105	85,988	0.9999
rs7901695	*TCF7L2*	0.031	1.41 [1.26-1.58]	7,046	85,319	0.9999
rs10886471	*GRK5*	0.7942	1.12 [1.08-1.16]	6,436	77,659	0.9984
rs4752781	*DUSP8/INS*	0.8328	1.35	7,094	85,838	-
rs2237892	*KCNQ1*	0.6764	1.40 [1.34-1.47]	7,095	85,896	0.9999
rs5215	*KCNJ11*	0.3861	1.14 [1.10-1.19]	7,109	86,011	0.9999
rs1552224	*ARAP1*	0.9161	1.14 [1.11-1.17]	7,108	86,022	0.9814
rs10830963	*MTNR1B*	0.4276	1.09 [1.06-1.12]	7,107	86,000	0.9984
rs1531343	*HMGA2*	0.09761	1.10 [1.07-1.14]	7,028	85,161	0.9087
rs7961581	*TSPAN8/LGR5*	0.2147	1.09 [1.06-1.12]	7,107	86,007	0.9843
rs1359790	*SPRY2*	0.7159	1.15 [1.10-1.20]	7,108	85,988	0.9999
rs7403531	*RASGRP1*	0.3503	1.10 [1.06-1.13]	6,434	77,641	0.9988
rs7172432	*VPS13C*	0.6182	1.11 [1.08-1.14]	7,108	85,987	0.9999
rs7178572	*HMG20A*	0.3496	1.09 [1.06-1.12]	7,109	86,018	0.9973
rs11634397	*ZFAND6*	0.08822	1.06 [1.04-1.08]	7,106	86,009	0.484
rs2028299	*AP3S2*	0.2017	1.10 [1.07-1.13]	7,108	86,007	0.9937
rs8042680	*PRC1*	0.9898	1.07 [1.05-1.09]	7,109	86,019	0.1194
rs9939609	*FTO*	0.1235	1.15 [1.09-1.23]	7,107	86,016	0.9997
rs4523957	*SRR*	0.7074	1.28 [1.18-1.39]	6,906	83,757	0.9999
rs4430796	*HNF1B*	0.2786	1.14 [1.08-1.20]	7,105	85,984	0.9999
rs12970134	*MC4R*	0.1881	1.08 [1.05-1.11]	7,100	85,952	0.9372
rs6017317	*FITM2-HNF4*	0.4263	1.09 [1.07-1.12]	7,108	86,015	0.9984
rs5945326	*DUSP9*	0.6049	1.27 [1.18-1.37]	7,091	85,888	0.9999

The statistical power shown in the table were calculated for association of each SNP with diabetes using the odds ratio reported in the original studies and sample size and risk allele frequencies in CKB study under an additive model. Power calculations were performed using odds ratios reported in the original GWAS and sample size and risk allele frequencies of our study with Quanto software (http://biostats.usc.edu/Quanto.html).
