# Supplementary material for: Evaluation of type 2 diabetes genetic risk variants in Chinese adults: findings from 93,000 individuals from the China Kadoorie Biobank
Source: Diabetologia. 2016 Apr 6;59:1446–57. doi: 10.1007/s00125-016-3920-9 (PMC4901105; doi:10.1007/s00125-016-3920-9)
Supplement: Supplementary file 5 — (PDF 10 kb) [file 125_2016_3920_MOESM5_ESM.pdf]

ESM Table 4 Heterogeneity of the genetic effect estimates between 10 different regional centre

CHR	SNP	Nearby Genes	I^2	[a] $p_{heterogeneity}$	Direction
1	rs10923931	*NOTCH2*	2.10	0.4192	+++-++++++
1	rs340874	*PROX1*	0.00	0.7609	++++-+++++
2	rs780094	*GCKR*	23.00	0.2319	+++-++++++
2	rs7578597	*THADA*	33.00	0.1443	++--+++++-
2	rs243021	*BCL11A*	43.00	0.0711	++-+-+++++
2	rs7593730	*RBMS1*	0.00	0.5492	-++---+---
2	rs3923113	*GRB14*	0.00	0.4811	-++++--+--
2	rs2943641	*IRS1*	42.80	0.0728	++-+----++
3	rs1801282	*PPARG*	0.00	0.4714	+++++--+-+
3	rs6780569	*UBE2E2*	0.00	0.9791	++++++++++
3	rs831571	*PSMD6*	22.50	0.2359	++-++++++-
3	rs4607103	*ADAMTS9*	47.30	0.0477	-++++----+
**3**	rs11708067	*ADCY5*	0.00	0.5730	++++-+++++
3	rs1470579	*IGF2BP2*	15.90	0.2971	+++++++-++
3	rs16861329	*ST64GAL1*	0.00	0.6314	++++-++-++
4	rs6815464	*MAEA*	1.60	0.4238	++++++-+++
4	rs10010131	*WFS1*	21.90	0.2418	++++-+-++-
5	rs4457053	*ZBED3*	53.10	0.0238	-+--++++++
6	rs7754840	*CDKAL1*	31.70	0.1546	++++++++++
6	rs9470794	*ZFAND3*	0.00	0.9912	++++----++
7	rs2191349	*DGKB*	0.00	0.6700	-+++---+++
7	rs864745	*JAZF1*	34.40	0.1323	-++-++-++-
7	rs4607517	*GCK*	57.30	0.0123	++-+++-+--
7	rs6467136	*GCC1-PAX4*	0.00	0.7055	-+++++++-+
7	rs972283	*KLF14*	0.00	0.6760	++-++++-++
8	rs896854	*TP53INP1*	0.00	0.9596	+++-++++++
8	rs13266634	*SLC30A8*	0.00	0.6537	++++++++++
9	rs7041847	*GLIS3*	0.00	0.4806	-+++++++++
9	rs17584499	*PTPRD*	0.00	0.8164	----+-++++
9	rs10811661	*CDKN2A/B*	59.80	0.0077	++++++++++
9	rs13292136	*TLE4/CHCHD*	19.60	0.2626	++++++-+++
10	rs10906115	*CDC123*	12.40	0.3289	++++++++++
10	rs1802295	*VPS26A*	0.00	0.4613	+-++++-+--
10	rs1111875	*HHEX/IDE*	0.00	0.5640	++++++++++
10	rs7901695	*TCF7L2*	0.00	0.5540	++++++++++
10	rs10886471	*GRK5*	13.10	0.3228	-+-++++--+
11	rs4752781	*DUSP8/INS*	22.70	0.2342	-+++-+---+
11	rs2237892	*KCNQ1*	50.20	0.0344	++++++++++
11	rs5215	*KCNJ11*	0.00	0.6773	+++++++-++
11	rs1552224	*ARAP1*	0.00	0.5431	++++--++++
11	rs10830963	*MTNR1B*	34.30	0.1339	+-+-++++--
12	rs1531343	*HMGA2*	0.00	0.7304	+-++-+++++
12	rs7961581	*TSPAN8/LGR5*	0.00	0.9621	+-++++++++
13	rs1359790	*SPRY2*	19.20	0.2659	-++-++++++
15	rs7403531	*RASGRP1*	4.00	0.4030	++-+++-+-+
15	rs7172432	*VPS13C*	24.50	0.2182	-++-++++++
15	rs7178572	*HMG20A*	0.00	0.8611	++++++++++
15	rs11634397	*ZFAND6*	0.00	0.7386	+-+-+-+++-
15	rs2028299	*AP3S2*	0.00	0.9898	+++++++++-
15	rs8042680	*PRC1*	6.60	0.3812	-++----+--
16	rs9939609	*FTO*	0.00	0.4839	++++++++++
17	rs4523957	*SRR*	56.40	0.0143	---++-+-+-
17	rs4430796	*HNF1B*	18.90	0.2687	++++++++++
18	rs12970134	*MC4R*	0.00	0.4380	+++++++--+
20	rs6017317	*HNF4A*	0.00	0.8093	+++++++++-
23	rs5945326	*DUSP9*	0.00	0.4421	++++++++++

[a]Evidence of heterogeneity ($P < 0.05 / 59 = 8.5E-04$)
