# Supplementary material for: Evaluation of type 2 diabetes genetic risk variants in Chinese adults: findings from 93,000 individuals from the China Kadoorie Biobank
Source: Diabetologia. 2016 Apr 6;59:1446–57. doi: 10.1007/s00125-016-3920-9 (PMC4901105; doi:10.1007/s00125-016-3920-9)
Supplement: Supplementary file 6 — (PDF 35 kb) [file 125_2016_3920_MOESM6_ESM.pdf]

ESM Table 5 Associations with diabetes after adjusting for BMI

SNP	Nearby Genes	R/A	Adjusted for age, sex, region and BMI	
			OR[95%CI]	*p*
rs10923931	*NOTCH2*	T/G	1.17[1.07-1.29]	$1.1x10^{-3}$
rs340874	*PROX1*	C/T	1.05[1.02-1.09]	$3.9x10^{-3}$
rs780094	*GCKR*	C/T	1.08[1.04-1.11]	$4.4x10^{-5}$
rs7578597	*THADA*	T/C	1.27[1.00-1.60]	$4.7x10^{-2}$
rs243021	*BCL11A*	A/G	1.07[1.03-1.12]	$2.1x10^{-4}$
rs7593730	*RBMS1*	C/T	0.98[0.93-1.02]	$3.3x10^{-1}$
rs3923113	*GRB14*	A/C	1.01[0.96-1.06]	$7.8x10^{-1}$
rs2943641	*IRS1*	C/T	1.05[0.98-1.12]	$1.7x10^{-1}$
rs1801282	*PPARG*	C/G	1.07[0.99-1.16]	$7.6x10^{-2}$
rs6780569	*UBE2E2*	G/A	1.12[1.07-1.17]	$4.8x10^{-7}$
rs831571	*PSMD6*	C/T	1.06[1.02-1.10]	$2.0x10^{-3}$
rs4607103	*ADAMTS9*	C/T	1.01[0.97-1.05]	$6.7x10^{-1}$
rs11708067	*ADCY5*	A/G	1.98[1.31-2.97]	$1.1x10^{-3}$
rs1470579	*IGF2BP2*	C/A	1.12[1.08-1.17]	$1.6x10^{-8}$
rs16861329	*ST64GAL1*	C/G	1.05[1.00-1.09]	$5.5x10^{-2}$
rs6815464	*MAEA*	C/G	1.08[1.04-1.12]	$1.3x10^{-5}$
rs10010131	*WFS1*	G/A	1.05[0.95-1.17]	$3.3x10^{-1}$
rs4457053	*ZBED3*	G/A	1.11[1.03-1.20]	$9.3x10^{-3}$
rs7754840	*CDKAL1*	C/G	1.24[1.19-1.28]	$2.8x10^{-31}$
rs9470794	*ZFAND3*	C/T	1.02[0.98-1.06]	$3.3x10^{-1}$
rs2191349	*DGKB*	T/G	1.05[1.00-1.09]	$3.2x10^{-2}$
rs864745	*JAZF1*	T/C	1.05[1.01-1.09]	$2.5x10^{-2}$
rs4607517	*GCK*	A/G	1.01[0.97-1.06]	$5.2x10^{-1}$
rs6467136	*GCC1-PAX4*	G/A	1.04[0.99-1.08]	$1.1x10^{-1}$
rs972283	*KLF14*	G/A	1.05[1.01-1.09]	$1.6x10^{-2}$
rs896854	*TP53INP1*	T/C	1.04[1.00-1.08]	$3.4x10^{-2}$
rs13266634	*SLC30A8*	C/T	1.10[1.07-1.14]	$4.9x10^{-9}$
rs7041847	*GLIS3*	A/G	1.07[1.03-1.10]	$3.3x10^{-4}$
rs17584499	*PTPRD*	T/C	1.01[0.95-1.07]	$8.6x10^{-1}$
rs10811661	*CDKN2A/B*	T/C	1.24[1.19-1.28]	$2.4x10^{-31}$
rs13292136	*TLE4/CHCHD9*	C/T	1.08[1.01-1.15]	$2.1x10^{-2}$
rs10906115	*CDC123*	A/G	1.09[1.05-1.13]	$2.5x10^{-6}$
rs1802295	*VPS26A*	T/G	1.02[0.97-1.08]	$4.0x10^{-1}$
rs1111875	*HHEX/IDE*	C/T	1.12[1.08-1.17]	$4.8x10^{-9}$
rs7901695	*TCF7L2*	C/T	1.40[1.27-1.53]	$7.1x10^{-13}$
rs10886471	*GRK5*	C/T	1.00[0.96-1.05]	$8.4x10^{-1}$
rs4752781	*DUSP8/INS*	T/A	0.99[0.95-1.04]	$7.0x10^{-1}$
rs2237892	*KCNQ1*	C/T	1.27[1.22-1.32]	$2.8x10^{-32}$
rs5215	*KCNJ11*	C/T	1.08[1.05-1.12]	$9.3x10^{-6}$
rs1552224	*ARAP1*	A/C	1.10[1.03-1.17]	$5.1x10^{-3}$
rs10830963	*MTNR1B*	G/C	1.02[0.99-1.06]	$2.1x10^{-1}$
rs1531343	*HMGA2*	C/G	1.05[0.99-1.11]	$1.2x10^{-1}$
rs7961581	*TSPAN8/LGR5*	C/T	1.04[1.00-1.09]	$4.9x10^{-2}$
rs1359790	*SPRY2*	G/A	1.06[1.02-1.10]	$5.5x10^{-3}$
rs7403531	*RASGRP1*	T/C	1.03[1.00-1.08]	$8.2x10^{-2}$
rs7172432	*VPS13C*	A/G	1.07[1.03-1.11]	$2.4x10^{-4}$
rs7178572	*HMG20A*	G/A	1.07[1.04-1.11]	$1.4x10^{-4}$
rs11634397	*ZFAND6*	G/A	1.02[0.96-1.09]	$5.2x10^{-1}$
rs2028299	*AP3S2*	C/A	1.07[1.02-1.11]	$3.6x10^{-3}$
rs8042680	*PRC1*	A/C	0.87[0.73-1.04]	$1.3x10^{-1}$
rs9939609	*FTO*	A/T	1.11[1.05-1.16]	$1.4x10^{-4}$
rs4523957	*SRR*	T/G	0.98[0.95-1.02]	$4.5x10^{-1}$
rs4430796	*HNF1B*	G/A	1.10[1.06-1.14]	$1.7x10^{-6}$
rs12970134	*MC4R*	A/G	1.03[0.99-1.08]	$1.3x10^{-1}$
rs6017317	*HNF4A*	G/T	1.05[1.02-1.09]	$5.0x10^{-3}$
rs5945326	*DUSP9*	A/G	1.11[1.07-1.16]	$2.3x10^{-7}$
