# Supplementary material for: Evaluation of type 2 diabetes genetic risk variants in Chinese adults: findings from 93,000 individuals from the China Kadoorie Biobank
Source: Diabetologia. 2016 Apr 6;59:1446–57. doi: 10.1007/s00125-016-3920-9 (PMC4901105; doi:10.1007/s00125-016-3920-9)
Supplement: Supplementary file 7 — (PDF 36 kb) [file 125_2016_3920_MOESM7_ESM.pdf]

**ESM Table 6 Associations of single-locus variants with diabetes after excluding those with screen-detected dia**

| SNP | Nearby Genes | R/A | Adjusted for age, sex, region | | Adjusted for age, sex, region and BMI | |
|---|---|---|---|---|---|---|
| | | | **OR[95%CI]** | ***p*** | **OR[95%CI]** | ***p*** |
| rs10923931 | *NOTCH2* | T/G | 1.17[1.05-1.31] | 3.7x10$^{-3}$ | 1.19[1.07-1.33] | 1.5x10$^{-3}$ |
| rs340874 | *PROX1* | C/T | 1.06[1.02-1.10] | 5.4x10$^{-3}$ | 1.06[1.02-1.11] | 4.4x10$^{-3}$ |
| rs780094 | *GCKR* | C/T | 1.08[1.04-1.13] | 9.0x10$^{-5}$ | 1.08[1.04-1.12] | 2.4x10$^{-4}$ |
| rs7578597 | *THADA* | T/C | 1.29[0.99-1.69] | 6.2x10$^{-2}$ | 1.29[0.99-1.70] | 6.4x10$^{-2}$ |
| rs243021 | *BCL11A* | A/G | 1.08[1.03-1.12] | 1.0x10$^{-3}$ | 1.08[1.03-1.12] | 8.4x10$^{-4}$ |
| rs7593730 | *RBMS1* | C/T | 0.97[0.92-1.02] | 2.6x10$^{-1}$ | 0.97[0.92-1.02] | 2.5x10$^{-1}$ |
| rs3923113 | *GRB14* | A/C | 0.98[0.92-1.04] | 4.6x10$^{-1}$ | 0.98[0.93-1.04] | 5.9x10$^{-1}$ |
| rs2943641 | *IRS1* | C/T | 1.04[0.96-1.12] | 3.3x10$^{-1}$ | 1.05[0.97-1.14] | 2.3x10$^{-1}$ |
| rs1801282 | *PPARG* | C/G | 1.08[0.98-1.18] | 1.2x10$^{-1}$ | 1.08[0.99-1.19] | 9.9x10$^{-2}$ |
| rs6780569 | *UBE2E2* | G/A | 1.13[1.07-1.19] | 3.4x10$^{-6}$ | 1.14[1.08-1.20] | 5.9x10$^{-7}$ |
| rs831571 | *PSMD6* | C/T | 1.07[1.03-1.12] | 1.4x10$^{-3}$ | 1.07[1.03-1.12] | 1.8x10$^{-3}$ |
| rs4607103 | *ADAMTS9* | C/T | 0.99[0.95-1.03] | 7.0x10$^{-1}$ | 1.00[0.96-1.04] | 8.5x10$^{-1}$ |
| rs11708067 | *ADCY5* | A/G | 2.39[1.40-4.08] | 1.4x10$^{-3}$ | 2.46[1.44-4.21] | 9.9x10$^{-4}$ |
| rs1470579 | *IGF2BP2* | C/A | 1.10[1.05-1.15] | 3.2x10$^{-5}$ | 1.11[1.06-1.16] | 7.8x10$^{-6}$ |
| rs16861329 | *ST64GAL1* | C/G | 1.06[1.00-1.11] | 3.8x10$^{-2}$ | 1.06[1.01-1.12] | 2.7x10$^{-2}$ |
| rs6815464 | *MAEA* | C/G | 1.07[1.02-1.11] | 1.9x10$^{-3}$ | 1.07[1.03-1.12] | 9.5x10$^{-4}$ |
| rs10010131 | *WFS1* | G/A | 1.10[0.97-1.24] | 1.3x10$^{-1}$ | 1.12[0.99-1.26] | 7.7x10$^{-2}$ |
| rs4457053 | *ZBED3* | G/A | 1.09[1.00-1.19] | 5.5x10$^{-2}$ | 1.10[1.01-1.20] | 3.3x10$^{-2}$ |
| rs7754840 | *CDKAL1* | C/G | 1.20[1.16-1.25] | 3.0x10$^{-19}$ | 1.23[1.18-1.28] | 1.9x10$^{-22}$ |
| rs9470794 | *ZFAND3* | C/T | 1.00[0.96-1.05] | 9.3x10$^{-1}$ | 1.00[0.96-1.05] | 8.5x10$^{-1}$ |
| rs2191349 | *DGKB* | T/G | 1.04[0.99-1.09] | 9.2x10$^{-2}$ | 1.04[0.99-1.09] | 8.5x10$^{-2}$ |
| rs864745 | *JAZF1* | T/C | 1.02[0.97-1.07] | 5.1x10$^{-1}$ | 1.02[0.97-1.07] | 3.7x10$^{-1}$ |
| rs4607517 | *GCK* | A/G | 1.02[0.97-1.07] | 4.5x10$^{-1}$ | 1.02[0.97-1.07] | 4.1x10$^{-1}$ |
| rs6467136 | *GCC1-PAX4* | G/A | 1.03[0.98-1.08] | 2.7x10$^{-1}$ | 1.02[0.97-1.07] | 4.1x10$^{-1}$ |
| rs972283 | *KLF14* | G/A | 1.05[1.01-1.10] | 2.0x10$^{-2}$ | 1.06[1.02-1.11] | 7.9x10$^{-3}$ |
| rs896854 | *TP53INP1* | T/C | 1.06[1.01-1.10] | 1.3x10$^{-2}$ | 1.06[1.02-1.11] | 8.3x10$^{-3}$ |
| rs13266634 | *SLC30A8* | C/T | 1.11[1.07-1.15] | 1.4x10$^{-7}$ | 1.11[1.07-1.16] | 2.8x10$^{-8}$ |
| rs7041847 | *GLIS3* | A/G | 1.06[1.02-1.10] | 5.3x10$^{-3}$ | 1.06[1.02-1.10] | 6.0x10$^{-3}$ |
| rs17584499 | *PTPRD* | T/C | 1.02[0.95-1.09] | 5.7x10$^{-1}$ | 1.02[0.96-1.09] | 5.0x10$^{-1}$ |
| rs10811661 | *CDKN2A/B* | T/C | 1.21[1.16-1.26] | 2.9x10$^{-20}$ | 1.23[1.18-1.28] | 6.8x10$^{-23}$ |
| rs13292136 | *TLE4/CHCHD9* | C/T | 1.05[0.98-1.13] | 2.0x10$^{-1}$ | 1.05[0.98-1.13] | 1.8x10$^{-1}$ |
| rs10906115 | *CDC123* | A/G | 1.08[1.04-1.13] | 1.4x10$^{-4}$ | 1.09[1.05-1.14] | 4.9x10$^{-5}$ |
| rs1802295 | *VPS26A* | T/G | 1.03[0.96-1.10] | 3.9x10$^{-1}$ | 1.03[0.96-1.10] | 4.0x10$^{-1}$ |
| rs1111875 | *HHEX/IDE* | C/T | 1.10[1.05-1.15] | 1.5x10$^{-5}$ | 1.11[1.07-1.16] | 2.0x10$^{-6}$ |
| rs7901695 | *TCF7L2* | C/T | 1.37[1.23-1.52] | 4.0x10$^{-9}$ | 1.39[1.25-1.54] | 1.2x10$^{-9}$ |
| rs10886471 | *GRK5* | C/T | 0.98[0.93-1.03] | 5.0x10$^{-1}$ | 0.99[0.93-1.04] | 5.7x10$^{-1}$ |
| rs4752781 | *DUSP8/INS* | T/A | 0.98[0.93-1.03] | 4.2x10$^{-1}$ | 0.98[0.93-1.03] | 3.9x10$^{-1}$ |
| rs2237892 | *KCNQ1* | C/T | 1.27[1.21-1.33] | 2.3x10$^{-25}$ | 1.28[1.23-1.34] | 2.0x10$^{-27}$ |
| rs5215 | *KCNJ11* | C/T | 1.08[1.03-1.12] | 4.7x10$^{-4}$ | 1.09[1.04-1.13] | 7.8x10$^{-5}$ |
| rs1552224 | *ARAP1* | A/C | 1.06[0.99-1.14] | 1.1x10$^{-1}$ | 1.07[0.99-1.16] | 6.8x10$^{-2}$ |
| rs10830963 | *MTNR1B* | G/C | 1.01[0.97-1.05] | 7.9x10$^{-1}$ | 1.01[0.97-1.05] | 7.8x10$^{-1}$ |
| rs1531343 | *HMGA2* | C/G | 1.05[0.98-1.12] | 1.3x10$^{-1}$ | 1.05[0.98-1.12] | 1.6x10$^{-1}$ |
| rs7961581 | *TSPAN8/LGR5* | C/T | 1.04[0.99-1.09] | 1.6x10$^{-1}$ | 1.04[0.99-1.09] | 1.3x10$^{-1}$ |
| rs1359790 | *SPRY2* | G/A | 1.07[1.03-1.12] | 2.1x10$^{-3}$ | 1.07[1.02-1.12] | 4.0x10$^{-3}$ |
| rs7403531 | *RASGRP1* | T/C | 1.04[0.99-1.08] | 1.1x10$^{-1}$ | 1.04[0.99-1.09] | 1.0x10$^{-1}$ |
| rs7172432 | *VPS13C* | A/G | 1.07[1.03-1.11] | 1.6x10$^{-3}$ | 1.07[1.03-1.12] | 9.5x10$^{-4}$ |
| rs7178572 | *HMG20A* | G/A | 1.07[1.02-1.11] | 3.2x10$^{-3}$ | 1.06[1.02-1.11] | 3.6x10$^{-3}$ |
| rs11634397 | *ZFAND6* | G/A | 1.01[0.94-1.08] | 8.9x10$^{-1}$ | 1.00[0.93-1.07] | 1.0E+00 |
| rs2028299 | *AP3S2* | C/A | 1.06[1.01-1.11] | 2.9x10$^{-2}$ | 1.06[1.01-1.12] | 1.9x10$^{-2}$ |
| rs8042680 | *PRC1* | A/C | 0.94[0.76-1.16] | 5.5x10$^{-1}$ | 0.93[0.75-1.15] | 5.1x10$^{-1}$ |
| rs9939609 | *FTO* | A/T | 1.17[1.10-1.24] | 2.9x10$^{-7}$ | 1.12[1.05-1.19] | 2.4x10$^{-4}$ |
| rs4523957 | *SRR* | T/G | 0.98[0.94-1.03] | 5.0x10$^{-1}$ | 0.99[0.95-1.04] | 6.4x10$^{-1}$ |
| rs4430796 | *HNF1B* | G/A | 1.08[1.03-1.13] | 5.3x10$^{-4}$ | 1.09[1.04-1.14] | 2.7x10$^{-4}$ |
| rs12970134 | *MC4R* | A/G | 1.06[1.01-1.12] | 2.3x10$^{-2}$ | 1.03[0.98-1.09] | 2.3x10$^{-1}$ |
| rs6017317 | *HNF4A* | G/T | 1.03[0.99-1.08] | 1.2x10$^{-1}$ | 1.04[1.00-1.08] | 7.4x10$^{-2}$ |
| rs5945326 | *DUSP9* | A/G | 1.11[1.06-1.17] | 4.1x10$^{-6}$ | 1.12[1.07-1.17] | 2.3x10$^{-6}$ |

Diabetes cases were either clinically-identified or incident diabetes (n=5,215 )
