# Supplementary material for: Evaluation of type 2 diabetes genetic risk variants in Chinese adults: findings from 93,000 individuals from the China Kadoorie Biobank
Source: Diabetologia. 2016 Apr 6;59:1446–57. doi: 10.1007/s00125-016-3920-9 (PMC4901105; doi:10.1007/s00125-016-3920-9)
Supplement: Supplementary file 8 — (PDF 9 kb) [file 125_2016_3920_MOESM8_ESM.pdf]

ESM Table 7 Concordance of T2D risk alleles

	CKB (56 variants overall)				East Asians: Meta-analysis of CKB and AGEN-T2D (55 variants overall)			
	[a] EA GWAS (15 loci)	[b] Others Ancestries (41 loci)	[c] Total (56)	Binomial test *P* (CKB into Total)	[a] EA GWAS (15 loci)	[b] Others Ancestries (40 loci)	[c] Total (55)	Binomial test *P* (CKB+AGEN-T2D into Other Ancestries)
Directionally consistent	12	36	48	48/56, $P = 2.34\times10^{-8}$	14	37	51	37/40, $P = 9.73\times10^{-9}$
Nominal Significant	9	26	35	-	12	25	37	-
Significant after correction for multiple-testing using the Holm-Bonferroni procedure	6	13	19	-	11	18	29	-
Genome-wide significant	1	4	5	-	9	10	19	-

Number of alleles are presented as [a] Reported in East Asians + [b] Others = [c] Total
