# Supplementary material for: Evaluation of type 2 diabetes genetic risk variants in Chinese adults: findings from 93,000 individuals from the China Kadoorie Biobank
Source: Diabetologia. 2016 Apr 6;59:1446–57. doi: 10.1007/s00125-016-3920-9 (PMC4901105; doi:10.1007/s00125-016-3920-9)
Supplement: Supplementary file 9 — (PDF 65 kb) [file 125_2016_3920_MOESM9_ESM.pdf]

ESM Table 8 Multiple testing adjusted *P* values using different methods

CHR	SNP	Nearby Genes	R/A	Unadjusted	Bonferroni	Benjamini-Hochberg (FDR)	Holm-Bonferroni	max (*T*) permutation (10000 times)	
								**Empirical *p***	**Corrected Empirical *p***
1	rs10923931	*NOTCH2*	T/G	$2.51x10^{-3}$	$1.41x10^{-1}$	$6.11x10^{-3}$ *	$8.71x10^{-2}$	$2.40x10^{-3}$	$1.76x10^{-1}$
1	rs340874	*PROX1*	C/T	$5.55x10^{-3}$	$3.11x10^{-1}$	$1.30x10^{-2}$ *	$1.83x10^{-1}$	$5.10x10^{-3}$	$3.31x10^{-1}$
2	rs780094	*GCKR*	C/T	$1.30x10^{-5}$	$7.25x10^{-4}$ *	$5.60x10^{-5}$ *	$5.70x10^{-4}$ *	$1.00x10^{-4}$	$1.60x10^{-3}$ *
2	rs7578597	*THADA*	T/C	$4.36x10^{-2}$	1	$7.10x10^{-2}$	1	$3.98x10^{-2}$	$9.45x10^{-1}$
2	rs243021	*BCL11A*	A/G	$3.36x10^{-4}$	$1.88x10^{-2}$ *	$1.05x10^{-3}$ *	$1.31x10^{-2}$ *	$6.00x10^{-4}$	$2.86x10^{-2}$ *
2	rs7593730	*RBMS1*	C/T	$3.45x10^{-1}$	1	$4.20x10^{-1}$	1	$3.43x10^{-1}$	1
2	rs3923113	*GRB14*	A/C	$9.69x10^{-1}$	1	$9.69x10^{-1}$	1	$9.67x10^{-1}$	1
2	rs2943641	*IRS1*	C/T	$2.48x10^{-1}$	1	$3.16x10^{-1}$	1	$2.51x10^{-1}$	1
3	rs1801282	*PPARG*	C/G	$1.03x10^{-1}$	1	$1.41x10^{-1}$	1	$1.06x10^{-1}$	$9.99x10^{-1}$
3	rs6780569	*UBE2E2*	G/A	$3.03x10^{-6}$	$1.70x10^{-4}$ *	$1.70x10^{-5}$ *	$1.42x10^{-4}$ *	$1.00x10^{-4}$	$6.00x10^{-4}$ *
3	rs831571	*PSMD6*	C/T	$1.48x10^{-3}$	$8.28x10^{-2}$	$4.13x10^{-3}$ *	$5.47x10^{-2}$	$1.20x10^{-3}$	$1.10x10^{-1}$
3	rs4607103	*ADAMTS9*	C/T	$8.58x10^{-1}$	1	$9.07x10^{-1}$	1	$8.57x10^{-1}$	1
**3**	rs11708067	*ADCY5*	A/G	$1.55x10^{-3}$	$8.66x10^{-2}$	$4.13x10^{-3}$ *	$5.57x10^{-2}$	$1.40x10^{-3}$	$1.16x10^{-1}$
3	rs1470579	*IGF2BP2*	C/A	$1.06x10^{-7}$	$5.94x10^{-6}$ *	$7.42x10^{-7}$ *	$5.20x10^{-6}$ *	$1.00x10^{-4}$	$1.00x10^{-4}$ *
3	rs16861329	*ST64GAL1*	C/G	$7.66x10^{-2}$	1	$1.13x10^{-1}$	1	$7.87x10^{-2}$	$9.94x10^{-1}$
4	rs6815464	*MAEA*	C/G	$3.39x10^{-5}$	$1.90x10^{-3}$ *	$1.36x10^{-4}$ *	$1.46x10^{-3}$ *	$1.00x10^{-4}$	$3.10x10^{-3}$ *
4	rs10010131	*WFS1*	G/A	$4.69x10^{-1}$	1	$5.25x10^{-1}$	1	$4.96x10^{-1}$	1
5	rs4457053	*ZBED3*	G/A	$1.64x10^{-2}$	$9.21x10^{-1}$	$3.17x10^{-2}$ *	$4.60x10^{-1}$	$1.89x10^{-2}$	$6.82x10^{-1}$
6	rs7754840	*CDKAL1*	C/G	$3.62x10^{-27}$	$2.03x10^{-25}$ *	$6.76x10^{-26}$ *	$1.95x10^{-25}$ *	$1.00x10^{-4}$	$1.00x10^{-4}$ *
6	rs9470794	*ZFAND3*	C/T	$4.06x10^{-1}$	1	$4.74x10^{-1}$	1	$4.29x10^{-1}$	1
7	rs2191349	*DGKB*	T/G	$3.46x10^{-2}$	1	$6.25x10^{-2}$	$8.98x10^{-1}$	$4.64x10^{-2}$	$9.04x10^{-1}$
7	rs864745	*JAZF1*	T/C	$3.82x10^{-2}$	1	$6.69x10^{-2}$	$9.56x10^{-1}$	$4.82x10^{-2}$	$9.24x10^{-1}$
7	rs4607517	*GCK*	A/G	$5.51x10^{-1}$	1	$6.05x10^{-1}$	1	$5.63x10^{-1}$	1
7	rs6467136	*GCC1-PAX4*	G/A	$6.46x10^{-2}$	1	$1.00x10^{-1}$	1	$7.53x10^{-2}$	$9.87x10^{-1}$
7	rs972283	*KLF14*	G/A	$4.16x10^{-2}$	1	$7.06x10^{-2}$	$9.98x10^{-1}$	$5.23x10^{-2}$	$9.37x10^{-1}$
8	rs896854	*TP53INP1*	T/C	$4.44x10^{-2}$	1	$7.10x10^{-2}$	1	$5.36x10^{-2}$	$9.48x10^{-1}$
8	rs13266634	*SLC30A8*	C/T	$3.41x10^{-8}$	$1.91x10^{-6}$ *	$3.82x10^{-7}$ *	$1.77x10^{-6}$ *	$1.00x10^{-4}$	$1.00x10^{-4}$ *
9	rs7041847	*GLIS3*	A/G	$2.89x10^{-4}$	$1.62x10^{-2}$ *	$9.52x10^{-4}$ *	$1.16x10^{-2}$ *	$1.20x10^{-3}$	$2.47x10^{-2}$ *
9	rs17584499	*PTPRD*	T/C	$9.33x10^{-1}$	1	$9.50x10^{-1}$	1	$9.37x10^{-1}$	1
9	rs10811661	*CDKN2A/B*	T/C	$7.09x10^{-28}$	$3.97x10^{-26}$ *	$1.99x10^{-26}$ *	$3.90x10^{-26}$ *	$1.00x10^{-4}$	$1.00x10^{-4}$ *
9	rs13292136	*TLE4/CHCHD9*	C/T	$2.18x10^{-2}$	1	$4.07x10^{-2}$ *	$5.89x10^{-1}$	$2.38x10^{-2}$	$7.76x10^{-1}$
10	rs10906115	*CDC123*	A/G	$1.08x10^{-5}$	$6.03x10^{-4}$ *	$5.04x10^{-5}$ *	$4.84x10^{-4}$ *	$1.00x10^{-4}$	$1.20x10^{-3}$ *
10	rs1802295	*VPS26A*	T/C	$3.82x10^{-1}$	1	$4.55x10^{-1}$	1	$3.94x10^{-1}$	1
10	rs1111875	*HHEX/IDE*	C/T	$6.17x10^{-8}$	$3.46x10^{-6}$ *	$4.94x10^{-7}$ *	$3.09x10^{-6}$ *	$1.00x10^{-4}$	$1.00x10^{-4}$ *
10	rs7901695	*TCF7L2*	C/T	$6.50x10^{-12}$	$3.64x10^{-10}$ *	$9.10x10^{-11}$ *	$3.45x10^{-10}$ *	$1.00x10^{-4}$	$1.00x10^{-4}$ *
10	rs10886471	*GRK5*	C/T	$9.02x10^{-1}$	1	$9.35x10^{-1}$	1	$9.02x10^{-1}$	1
11	rs4752781	*DUSP8/INS*	T/A	$7.34x10^{-1}$	1	$7.90x10^{-1}$	1	$7.39x10^{-1}$	1
11	rs2237892	*KCNQ1*	C/T	$4.61x10^{-30}$	$2.58x1^{-28}$ *	$2.58x10^{-28}$ *	$2.58x10^{-28}$ *	$1.00x10^{-4}$	$1.00x10^{-4}$ *
11	rs5215	*KCNJ11*	C/T	$7.85x10^{-5}$	$4.40x10^{-3}$ *	$2.93x10^{-4}$ *	$3.30x10^{-3}$ *	$3.00x10^{-4}$	$7.70x10^{-3}$ *
11	rs1552224	*ARAP1*	A/C	$1.07x10^{-2}$	$5.98x10^{-1}$	$2.14x10^{-2}$ *	$3.10x10^{-1}$	$1.54x10^{-2}$	$5.33x10^{-1}$
11	rs10830963	*MTNR1B*	G/C	$2.04x10^{-1}$	1	$2.66x10^{-1}$	1	$2.58x10^{-1}$	1
12	rs1531343	*HMGA2*	C/G	$9.92x10^{-2}$	1	$1.39x10^{-1}$	1	$1.11x10^{-1}$	$9.99x10^{-1}$
12	rs7961581	*TSPAN8/LGR5*	C/T	$7.09x10^{-2}$	1	$1.07x10^{-1}$	1	$8.15x10^{-2}$	$9.92x10^{-1}$
13	rs1359790	*SPRY2*	G/A	$2.49x10^{-3}$	$1.39x10^{-1}$	$6.11x10^{-3}$ *	$8.71x10^{-2}$	$3.60x10^{-3}$	$1.76x10^{-1}$
15	rs7403531	*RASGRP1*	T/C	$9.27x10^{-2}$	1	$1.33x10^{-1}$	1	$1.29x10^{-1}$	$9.98x10^{-1}$
15	rs7172432	*VPS13C*	A/G	$4.32x10^{-4}$	$2.42x10^{-2}$ *	$1.27x10^{-3}$ *	$1.64x10^{-2}$ *	$8.00x10^{-4}$	$3.64x10^{-2}$ *
15	rs7178572	*HMG20A*	G/A	$1.25x10^{-4}$	$7.00x10^{-3}$ *	$4.38x10^{-4}$ *	$5.13x10^{-3}$ *	$2.00x10^{-4}$	$1.15x10^{-2}$ *
15	rs11634397	*ZFAND6*	G/A	$4.46x10^{-1}$	1	$5.10x10^{-1}$	1	$4.44x10^{-1}$	1
15	rs2028299	*AP3S2*	C/A	$6.04x10^{-3}$	$3.38x10^{-1}$	$1.35x10^{-2}$ *	$1.93x10^{-1}$	$6.60x10^{-3}$	$3.52x10^{-1}$
15	rs8042680	*PRC1*	A/C	$1.45x10^{-1}$	1	$1.93x10^{-1}$	1	$1.43x10^{-1}$	1
16	rs9939609	*FTO*	A/T	$5.41x10^{-8}$	$3.03x10^{-6}$ *	$4.94x10^{-7}$ *	$2.76x10^{-6}$ *	$1.00x10^{-4}$	$1.00x10^{-4}$ *
17	rs4523957	*SRR*	T/G	$3.23x10^{-1}$	1	$4.02x10^{-1}$	1	$3.26x10^{-1}$	1
17	rs4430796	*HNF1B*	G/A	$3.81x10^{-6}$	$2.13x10^{-4}$ *	$1.94x10^{-5}$ *	$1.75x10^{-4}$ *	$1.00x10^{-4}$	$6.00x10^{-4}$ *
18	rs12970134	*MC4R*	A/G	$6.76x10^{-3}$	$3.79x10^{-1}$	$1.46x10^{-2}$ *	$2.10x10^{-1}$	$7.30x10^{-3}$	$3.81x10^{-1}$
20	rs6017317	*HNF4A*	G/T	$9.94x10^{-3}$	$5.56x10^{-1}$	$2.06x10^{-2}$ *	$2.98x10^{-1}$	$1.11x10^{-2}$	$5.08x10^{-1}$
23	rs5945326	*DUSP9*	A/G	$4.08x10^{-7}$	$2.28x10^{-5}$ *	$2.54x10^{-6}$ *	$1.96x10^{-5}$ *	$1.00x10^{-4}$	$1.00x10^{-4}$ *

*$p < 0.05$ after adjustment for multiple comparisons using the Bonferroni, Benjamini-Hochberg(false discovery rate), Holm-Bonferroni procedure or 10000 times-max (*T*) permutation
