# Supplementary material for: Evaluation of type 2 diabetes genetic risk variants in Chinese adults: findings from 93,000 individuals from the China Kadoorie Biobank
Source: Diabetologia. 2016 Apr 6;59:1446–57. doi: 10.1007/s00125-016-3920-9 (PMC4901105; doi:10.1007/s00125-016-3920-9)
Supplement: Supplementary file 10 — (PDF 131 kb) [file 125_2016_3920_MOESM10_ESM.pdf]

ESM Table 9 Heterogeneity in allelic effects between Chinese, East Asians and Europeans

CHR	SNP	Nearby Genes	R/A	OR[95%CI]					*P* for heterogeneity			Reported Population
				CKB [Up to 7,109 cases and 86,022 controls]	AGEN-T2D [Up to 25,079 cases and 29,611 controls]	Meta-analysis of CKB+AGEN-T2D [a] [up to32,188 cases and 115,633 controls]	[b] DIAGRAMv3 (EU) [up to 12,171 cases and 56,862 controls]	Meta-analysis of CKB+Trans-ethnic GWAS [c] [up to 33,597 cases and 201,655 controls]	CKB vs. DIAGRAMv3	Meta vs. DIAGRAMv3		
1	rs10923931	*NOTCH2*	T/G	1.16[1.05-1.27]	1.00[0.86-1.16]	1.11[1.02-1.20]	1.10[1.05-1.16]	1.06[1.03-1.10]	$3.93x10^{-1}$	$9.17x10^{-1}$		EU
1	rs340874	*PROX1*	C/T	1.05[1.01-1.09]	1.08[1.03-1.14]	1.06[1.03-1.09]	1.08[1.04-1.12]	1.06[1.04-1.08]	$3.24x10^{-1}$	$5.05x10^{-1}$		EU
2	rs780094	*GCKR*	C/T	1.08[1.04-1.12]	1.06[1.01-1.11]	1.07[1.04-1.10]	1.04[1.00-1.08]	1.07[1.04-1.09]	$1.41x10^{-1}$	$1.90x10^{-1}$		EU
2	rs7578597	*THADA*	T/C	1.27[1.01-1.60]	0.93[0.62-1.40]	1.18[0.96-1.44]	1.14[1.08-1.22]	1.08[1.03-1.13]	$4.14x10^{-1}$	$8.18x10^{-1}$		EU
2	rs243021	*BCL11A*	A/G	1.07[1.03-1.11]	1.05[1.00-1.10]	1.06[1.03-1.09]	1.09[1.05-1.13]	1.07[1.05-1.09]	$5.23x10^{-1}$	$2.98x10^{-1}$		EU
2	rs7593730	*RBMS1*	C/T	0.98[0.93-1.02]	1.00[0.94-1.07]	0.99[0.95-1.02]	1.11[1.06-1.15]	1.03[1.00-1.06]	$1.22x10^{-4}$	$7.05x10^{-5}$	*	EU
2	rs3923113	*GRB14*	A/C	1.00[0.95-1.05]	1.03[0.95-1.12]	1.01[0.97-1.05]	1.04[1.00-1.08]	1.05[1.02-1.08]	$2.51x10^{-1}$	$3.26x10^{-1}$		SA
2	rs2943641	*IRS1*	C/T	1.04[0.97-1.11]	1.12[1.03-1.22]	1.07[1.02-1.13]	1.08[1.04-1.12]	1.08[1.05-1.12]	$3.55x10^{-1}$	$8.21x10^{-1}$		EU
3	rs1801282	*PPARG*	C/G	1.07[0.99-1.16]	1.15[1.01-1.30]	1.09[1.02-1.16]	1.16[1.11-1.22]	1.12[1.08-1.17]	$6.74x10^{-2}$	$1.14x10^{-1}$		EU
3	rs6780569	*UBE2E2*	G/A	1.11[1.06-1.16]	1.17[1.12-1.22]	1.14[1.10-1.18]	1.09[1.02-1.15]	1.11[1.08-1.14]	$4.85x10^{-1}$	$1.40x10^{-1}$		EA
3	rs831571	*PSMD6*	C/T	1.06[1.02-1.10]	1.09[1.05-1.13]	1.08[1.05-1.11]	1.03[0.99-1.08]	1.05[1.03-1.08]	$3.78x10^{-1}$	$1.13x10^{-1}$		EA
3	rs4607103	*ADAMTS9*	C/T	1.00[0.97-1.04]	0.99[0.95-1.04]	1.00[0.97-1.03]	1.08[1.04-1.12]	1.03[1.00-1.05]	$5.77x10^{-3}$	$1.27x10^{-3}$		EU
3	rs11708067	*ADCY5*	A/G	1.92[1.28-2.88]	1.18[0.80-1.74]	1.49[1.13-1.97]	1.10[1.06-1.15]	1.11[1.07-1.14]	$7.53x10^{-3}$	$3.75x10^{-2}$		**EU**
3	rs1470579	*IGF2BP2*	C/A	1.11[1.07-1.16]	1.15[1.11-1.19]	1.13[1.10-1.16]	1.12[1.08-1.16]	1.12[1.10-1.15]	$7.97x10^{-1}$	$6.38x10^{-1}$		EU
3	rs16861329	*ST64GAL1*	C/G	1.04[1.00-1.09]	0.92[0.86-0.99]	1.01[0.97-1.04]	1.03[0.97-1.09]	1.07[1.04-1.10]	$7.37x10^{-1}$	$5.31x10^{-1}$		SA
4	rs6815464	*MAEA*	C/G	1.08[1.04-1.12]	1.13[1.10-1.16]	1.11[1.09-1.13]	1.12[1.01-1.24]	1.08[1.05-1.12]	$4.94x10^{-1}$	$8.85x10^{-1}$		EA
4	rs10010131	*WFS1*	G/A	1.04[0.94-1.15]	1.00[0.91-1.10]	1.02[0.95-1.09]	1.10[1.06-1.14]	1.09[1.06-1.12]	$3.06x10^{-1}$	$5.48x10^{-2}$		EU
5	rs4457053	*ZBED3*	G/A	1.10[1.02-1.18]	1.00[0.85-1.18]	1.08[1.01-1.16]	1.13[1.08-1.19]	1.10[1.06-1.14]	$4.79x10^{-1}$	$2.57x10^{-1}$		EU
6	rs7754840	*CDKAL1*	C/G	1.21[1.17-1.26]	1.18[1.14-1.22]	1.20[1.17-1.23]	1.15[1.11-1.19]	1.17[1.15-1.20]	$3.06x10^{-2}$	$6.39x10^{-2}$		EU
6	rs9470794	*ZFAND3*	C/T	1.02[0.98-1.05]	1.12[1.08-1.16]	1.07[1.04-1.10]	0.99[0.93-1.05]	1.04[1.01-1.07]	$4.49x10^{-1}$	$2.47x10^{-2}$		EA
7	rs2191349	*DGKB*	T/G	1.05[1.00-1.09]	1.12[1.08-1.17]	1.09[1.06-1.12]	1.07[1.03-1.11]	1.08[1.05-1.10]	$4.35x10^{-1}$	$5.15x10^{-1}$		EU
7	rs864745	*JAZF1*	T/C	1.04[1.00-1.09]	1.06[1.00-1.12]	1.05[1.01-1.09]	1.12[1.08-1.15]	1.08[1.06-1.11]	$1.51x10^{-2}$	$1.04x10^{-2}$		EU
7	rs4607517	*GCK*	A/G	1.01[0.97-1.06]	1.03[0.97-1.09]	1.02[0.98-1.05]	1.05[1.00-1.10]	1.03[1.00-1.05]	$2.86x10^{-1}$	$3.24x10^{-1}$		EU
7	rs6467136	*GCC1-PAX4*	G/A	1.04[1.00-1.09]	1.11[1.07-1.14]	1.08[1.05-1.11]	0.99[0.95-1.02]	1.02[1.00-1.04]	$5.13x10^{-2}$	$2.28x10^{-5}$	*	EA
7	rs972283	*KLF14*	G/A	1.04[1.00-1.08]	0.99[0.93-1.06]	1.03[0.99-1.06]	1.10[1.06-1.14]	1.05[1.02-1.07]	$4.42x10^{-2}$	$7.98x10^{-3}$		EU
8	rs896854	*TP53INP1*	T/C	1.04[1.00-1.08]	1.07[1.02-1.12]	1.05[1.02-1.08]	1.09[1.05-1.13]	1.07[1.04-1.09]	$7.94x10^{-2}$	$1.33x10^{-1}$		EU
8	rs13266634	*SLC30A8*	C/T	1.10[1.06-1.13]	1.10[1.07-1.14]	1.10[1.07-1.13]	1.16[1.11-1.22]	1.12[1.10-1.15]	$4.51x10^{-2}$	$3.98x10^{-2}$		EU
9	rs7041847	*GLIS3*	A/G	1.07[1.03-1.10]	1.10[1.07-1.13]	1.09[1.06-1.11]	1.05[1.01-1.09]	1.06[1.04-1.08]	$5.36x10^{-1}$	$1.14x10^{-1}$		EA
9	rs17584499	*PTPRD*	T/C	1.00[0.95-1.06]	1.09[1.00-1.19]	1.03[0.98-1.08]	1.00[0.95-1.06]	1.01[0.97-1.04]	$9.80x10^{-1}$	$5.03x10^{-1}$		EA
9	rs10811661	*CDKN2A/B*	T/C	1.22[1.18-1.26]	1.12[1.07-1.16]	1.17[1.14-1.21]	1.18[1.13-1.23]	1.21[1.18-1.24]	$2.36x10^{-1}$	$8.50x10^{-1}$		EU
9	rs13292136	*TLE4/CHCHD9*	C/T	1.08[1.01-1.14]	0.99[0.92-1.07]	1.04[0.99-1.09]	1.19[1.11-1.27]	1.10[1.07-1.14]	$3.47x10^{-2}$	$1.84x10^{-3}$		EU
10	rs10906115	*CDC123*	A/G	1.08[1.05-1.12]	1.08[1.05-1.13]	1.08[1.06-1.11]	1.06[1.02-1.10]	1.08[1.05-1.10]	$3.72x10^{-1}$	$3.07x10^{-1}$		EA
10	rs1802295	*VPS26A*	T/C	1.03[0.97-1.08]	1.01[0.94-1.09]	1.02[0.98-1.07]	1.02[0.98-1.06]	1.04[1.02-1.07]	$8.68x10^{-1}$	$9.68x10^{-1}$		SA
10	rs1111875	*HHEX/IDE*	C/T	1.11[1.07-1.15]	1.08[1.04-1.13]	1.10[1.07-1.13]	1.15[1.11-1.18]	1.12[1.09-1.14]	$2.41x10^{-1}$	$5.15x10^{-2}$		EU
10	rs7901695	*TCF7L2*	C/T	1.37[1.25-1.50]	1.18[1.03-1.35]	1.31[1.21-1.41]	1.37[1.32-1.42]	1.32[1.28-1.35]	$9.80x10^{-1}$	$2.90x10^{-1}$		EU
10	rs10886471	*GRK5*	C/T	1.00[0.96-1.05]	1.06[0.99-1.13]	1.02[0.98-1.06]	0.99[0.95-1.03]	1.01[0.98-1.03]	$6.95x10^{-1}$	$2.96x10^{-1}$		EA
11	rs4752781	*DUSP8/INS*	T/A	0.99[0.95-1.04]	1.04[0.98-1.10]	1.01[0.97-1.05]	1.04[1.01-1.08]	1.03[1.00-1.05]	$7.73x10^{-2}$	$1.81x10^{-1}$		EU
11	rs2237892	*KCNQ1*	C/T	1.25[1.20-1.30]	1.19[1.14-1.24]	1.22[1.19-1.26]	1.12[1.04-1.21]	1.23[1.20-1.27]	$1.17x10^{-2}$	$3.88x10^{-2}$		EA
11	rs5215	*KCNJ11*	C/T	1.07[1.04-1.11]	1.10[1.06-1.14]	1.09[1.06-1.11]	1.08[1.05-1.12]	1.08[1.06-1.10]	$7.05x10^{-1}$	$9.17x10^{-1}$		EU
11	rs1552224	*ARAP1*	A/C	1.09[1.02-1.16]	1.16[1.05-1.28]	1.11[1.05-1.17]	1.13[1.08-1.19]	1.10[1.06-1.14]	$3.18x10^{-1}$	$5.50x10^{-1}$		EU
11	rs10830963	*MTNR1B*	G/C	1.02[0.99-1.06]	1.00[0.93-1.08]	1.02[0.99-1.05]	1.11[1.07-1.16]	1.06[1.03-1.08]	$1.81x10^{-3}$	$6.95x10^{-4}$		EU
12	rs1531343	*HMGA2*	C/G	1.05[0.99-1.11]	1.06[0.99-1.14]	1.05[1.01-1.10]	1.15[1.09-1.22]	1.08[1.05-1.12]	$2.02x10^{-2}$	$1.40x10^{-2}$		EU
12	rs7961581	*TSPAN8/LGR5*	C/T	1.04[1.00-1.08]	1.01[0.95-1.06]	1.03[0.99-1.06]	1.08[1.04-1.12]	1.05[1.03-1.07]	$1.88x10^{-1}$	$4.65x10^{-2}$		EU
13	rs1359790	*SPRY2*	G/A	1.06[1.02-1.10]	1.05[1.01-1.10]	1.06[1.03-1.09]	1.10[1.06-1.14]	1.07[1.04-1.09]	$2.06x10^{-1}$	$9.87x10^{-2}$		EA
15	rs7403531	*RASGRP1*	T/C	1.03[0.99-1.07]	1.08[1.02-1.13]	1.05[1.02-1.08]	1.02[0.98-1.06]	1.02[1.00-1.05]	$6.23x10^{-1}$	$2.81x10^{-1}$		EA
15	rs7172432	*VPS13C*	A/G	1.07[1.03-1.11]	1.11[1.07-1.15]	1.09[1.06-1.11]	1.06[1.02-1.10]	1.07[1.05-1.09]	$7.82x10^{-1}$	$2.68x10^{-1}$		EA
15	rs7178572	*HMG20A*	G/A	1.07[1.04-1.11]	1.09[1.04-1.14]	1.08[1.05-1.11]	1.08[1.04-1.12]	1.08[1.06-1.11]	$8.48x10^{-1}$	$9.90x10^{-1}$		SA
15	rs11634397	*ZFAND6*	G/A	1.02[0.96-1.09]	1.00[0.90-1.11]	1.02[0.97-1.07]	1.09[1.05-1.13]	1.06[1.03-1.09]	$9.09x10^{-2}$	$3.89x10^{-2}$		EU
15	rs2028299	*AP3S2*	C/A	1.06[1.02-1.11]	1.08[1.02-1.14]	1.07[1.03-1.11]	1.04[1.00-1.09]	1.07[1.04-1.09]	$5.81x10^{-1}$	$4.15x10^{-1}$		SA
15	rs8042680	*PRC1*	A/C	0.88[0.74-1.05]	1.64[1.16-2.32]	1.00[0.85-1.16]	1.07[1.04-1.11]	1.06[1.04-1.09]	$2.59x10^{-2}$	$3.50x10^{-1}$		EU
16	rs9939609	*FTO*	A/T	1.15[1.09-1.21]	1.13[1.07-1.18]	1.14[1.10-1.18]	1.11[1.07-1.14]	1.11[1.08-1.14]	$1.68x10^{-1}$	$2.12x10^{-1}$		EU
17	rs4523957	*SRR*	T/G	0.98[0.94-1.02]	1.03[0.97-1.09]	0.99[0.96-1.03]	1.00[0.96-1.04]	1.00[0.98-1.03]	$4.74x10^{-1}$	$8.24x10^{-1}$		EA
17	rs4430796	*HNF1B*	G/A	1.09[1.05-1.14]	1.12[1.05-1.19]	1.10[1.07-1.14]	1.13[1.07-1.19]	1.10[1.07-1.13]	$3.54x10^{-1}$	$4.30x10^{-1}$		EU
18	rs12970134	*MC4R*	A/G	1.06[1.02-1.11]	1.07[1.02-1.12]	1.07[1.03-1.10]	1.08[1.04-1.12]	1.07[1.05-1.10]	$5.94x10^{-1}$	$6.49x10^{-1}$		EU
20	rs6017317	*HNF4A*	G/T	1.05[1.01-1.08]	1.09[1.07-1.12]	1.08[1.06-1.10]	1.06[1.01-1.12]	1.06[1.04-1.08]	$6.31x10^{-1}$	$5.75x10^{-1}$		EA
23	rs5945326	*DUSP9*	A/G	1.11[1.07-1.15]	NA	NA	NA	NA	NA	NA		EU

Meta: Meta-analysis of AGEN-T2D and CKB study; EU: Europeans; SA: South Asians; EA: East Asians;CKB: China Kadoorie Biobank

[a]AGEN-T2D: T2D-GWAS meta-analysis in East Asian ; [b]DIAGRAM: DIAbetes Genetics Replication And Meta-analysis

[c]Trans-ethnic GWAS: Trans-ethnic Meta analysis of four ethinic groups (East Asians[AGEN-T2D], Europeans [DIAGRAM], South Asians [SAT2D], and Mexicans [MAT2D])

Reference(PMID): [a]22158537; [b]22885922; [c]24509480; * SNPs showing nominal evidence of heterogeneity (p <0.05/110=0.00045)
