# Supplementary material for: Evaluation of type 2 diabetes genetic risk variants in Chinese adults: findings from 93,000 individuals from the China Kadoorie Biobank
Source: Diabetologia. 2016 Apr 6;59:1446–57. doi: 10.1007/s00125-016-3920-9 (PMC4901105; doi:10.1007/s00125-016-3920-9)
Supplement: Supplementary file 11 — (PDF 100 kb) [file 125_2016_3920_MOESM11_ESM.pdf]

ESM Table 10 Comparison of LD structure between populations

Region	SNP	Nearby Genes	*p* value
chr1:120214000..120416000	rs10923931	*NOTCH2*	$7.70x10^{-1}$
chr1:212127000..212261000	rs340874	*PROX1*	$1.00x10^{-4}$
chr10:114732000..114809000	rs7901695	*TCF7L2*	$1.24x10^{-2}$
chr2:160787000..161145000	[a] rs7593730	*RBMS1*	$2.00x10^{-4}$
chr2:165208000..165346000	rs3923113	*GRB14*	$1.88x10^{-2}$
chr2:226729000..226896000	rs2943641	*IRS1*	$1.00x10^{-4}$
chr2:27400000..27619999	rs780094	*GCKR*	$6.72x10^{-2}$
chr2:43520000..43685000	rs7578597	*THADA*	$1.00x10^{-4}$
chr2:60383000..60457000	rs243021	*BCL11A*	$1.00x10^{-4}$
chr20:42282000..42432000	rs6017317	*HNF4A*	$1.00x10^{-4}$
chr3:12249000..12443000	rs1801282	*PPARG*	$1.00x10^{-4}$
chr3:124432000..124613000	rs11708067	*ADCY5*	$1.10x10^{-3}$
chr3:186950000..187048000	rs1470579	*IGF2BP2*	$2.60x10^{-3}$
chr3:188085000..188166000	rs16861329	*ST64GAL1*	$3.35x10^{-1}$
chr3:23217000..23341000	rs6780569	*UBE2E2*	$6.49x10^{-2}$
chr3:63924000..64091000	rs831571	*PSMD6*	$1.00x10^{-4}$
chr3:64670000..64780000	rs4607103	*ADAMTS9*	$1.00x10^{-4}$
chr4:1192000..1407000	rs6815464	*MAEA*	$1.00x10^{-4}$
chr4:6303000..6383000	rs10010131	*WFS1*	$1.00x10^{-4}$
chr5:76417000..76547000	rs4457053	*ZBED3*	$1.30x10^{-3}$
chr6:20636000..20838000	rs7754840	*CDKAL1*	$1.00x10^{-4}$
chr6:38115000..38270000	rs9470794	*ZFAND3*	$1.00x10^{-4}$
chr7:126849000..127059000	[a] rs6467136	*GCC1-PAX4*	$1.00x10^{-4}$
chr7:130074000..130122000	rs972283	*KLF14*	$1.91x10^{-1}$
chr7:14981000..15036000	rs2191349	*DGKB*	$5.08x10^{-2}$
chr7:28099000..28229000	rs864745	*JAZF1*	$1.00x10^{-4}$
chr7:44188000..44233000	rs4607517	*GCK*	$3.40x10^{-3}$
chr8:118244000..118307000	rs13266634	*SLC30A8*	$1.00x10^{-4}$
chr8:95904000..96078000	rs896854	*TP53INP1*	$1.00x10^{-4}$
chr9:22117000..22130000	rs10811661	*CDKN2A/B*	$1.00x10^{-4}$
chr9:4230000..4286000	rs7041847	*GLIS3*	$3.07x10^{-2}$
chr9:81069000..81204000	rs13292136	*TLE4/CHCHD9*	$3.89x10^{-2}$
chr9:8802000..8885000	rs17584499	*PTPRD*	$3.40x10^{-3}$
chr10:121047000..121207000	rs10886471	*GRK5*	$2.65x10^{-1}$
chr10:12259000..12382000	rs10906115	*CDC123*	$5.68x10^{-2}$
chr10:70467000..70641000	rs1802295	*VPS26A*	$1.00x10^{-4}$
chr10:94436000..94491000	rs1111875	*HHEX/IDE*	$3.00x10^{-4}$
chr11:1642000..1673000	rs4752781	*DUSP8/INS*	$2.00x10^{-4}$
chr11:17259000..17384000	rs5215	*KCNJ11*	$2.89x10^{-2}$
chr11:2774000..2816000	rs2237892	*KCNQ1*	$1.00x10^{-4}$
chr11:72009000..72158000	rs1552224	*ARAP1*	$6.00x10^{-4}$
chr11:92306000..92368000	rs10830963	*MTNR1B*	$1.26x10^{-2}$
chr12:64448000..64598000	rs1531343	*HMGA2*	$6.90x10^{-3}$
chr12:69780000..69953000	rs7961581	*TSPAN8/LGR5*	$3.24x10^{-2}$
chr13:79493000..79675000	rs1359790	*SPRY2*	$2.10x10^{-3}$
chr15:36601000..36716000	rs7403531	*RASGRP1*	$8.60x10^{-3}$
chr15:60170000..60226000	rs7172432	*VPS13C*	$2.00x10^{-4}$
chr15:75472000..75589000	rs7178572	*HMG20A*	$2.00x10^{-4}$
chr15:78127000..78227000	rs11634397	*ZFAND6*	$1.00x10^{-4}$
chr15:88155000..88275000	rs2028299	*AP3S2*	$1.00x10^{-4}$
chr15:89248000..89378000	rs8042680	*PRC1*	$1.00x10^{-4}$
chr16:52354000..52405000	rs9939609	*FTO*	$3.00x10^{-4}$
chr17:2006000..2203000	rs4523957	*SRR*	$1.00x10^{-4}$
chr17:33168000..33208000	rs4430796	*HNF1B*	$5.62x10^{-1}$
chr18:55900000..56067000	rs12970134	*MC4R*	$2.29x10^{-1}$

Monte-Carlo *P* values were generated from varLD algorithm by comparing pairwise r2 of SNPs in each genomic region between East Asians (CHB+JPT panel) and Europeans (CEU panel) from HapMap release #27

[a] SNPs showing nominal evidence of heterogeneity of the effect size estimates in East Asian and Europeans
