# Supplementary material for: Evaluation of type 2 diabetes genetic risk variants in Chinese adults: findings from 93,000 individuals from the China Kadoorie Biobank
Source: Diabetologia. 2016 Apr 6;59:1446–57. doi: 10.1007/s00125-016-3920-9 (PMC4901105; doi:10.1007/s00125-016-3920-9)
Supplement: Supplementary file 12 — (PDF 31 kb) [file 125_2016_3920_MOESM12_ESM.pdf]

ESM Table 11 Comparison of effect size and discriminative ability of the GRSs for risk of diabetes

GRS	Weights	AUC	*p*
GRS-T(52SNPs)			
	Unweighted	0.574[0.567-0.580]	ref
	AGEN-T2D	0.582[0.575-0.589]	$6.8x10^{-5}$
	DIAGRAMv3	0.584[0.577-0.591]	$1.4x10^{-7}$
	MetaboChip	0.588[0.581-0.595]	$6.4x10^{-12}$
	TransEthnic	0.590[0.583-0.597]	$3.6x10^{-20}$
	TransEthnic+CKB	0.593[0.586-0.600]	$4.3x10^{-23}$
	TransEthnic+CKB (1000-fold cross-validation)	0.591[0.584-0.598]	$1.8x10^{-19}$
GRS-BC (25 SNPs)			
	Unweighted	0.569[0.562-0.576]	ref
	AGEN-T2D	0.576[0.570-0.583]	$7.6x10^{-3}$
	DIAGRAMv3	0.577[0.570-0.584]	$7.2x10^{-6}$
	MetaboChip	0.580[0.573-0.587]	$2.1x10^{-9}$
	TransEthnic	0.581[0.574-0.588]	$2.0x10^{-14}$
	TransEthnic+CKB	0.583[0.576-0.590]	$3.6x10^{-15}$
	TransEthnic+CKB (1000-fold cross-validation)	0.582[0.575-0.589]	$1.5x10^{-13}$
GRS-IR (7 SNPs)			
	Unweighted	0.521[0.514-0.527]	ref
	AGEN-T2D	0.521[0.514-0.528]	0.996
	DIAGRAMv3	0.518[0.511-0.525]	0.187
	MetaboChip	0.520[0.513-0.527]	0.749
	TransEthnic	0.520[0.514-0.527]	0.907
	TransEthnic+CKB	0.522[0.515-0.529]	0.176
	TransEthnic+CKB (1000-fold cross-validation)	0.520[0.513-0.527]	0.343

The range of all GRSs were rescaled in the range of unweigted GRS-T

P-value derived from DeLong test compared to unweighted GRS model
