# Supplementary material for: Evaluation of type 2 diabetes genetic risk variants in Chinese adults: findings from 93,000 individuals from the China Kadoorie Biobank
Source: Diabetologia. 2016 Apr 6;59:1446–57. doi: 10.1007/s00125-016-3920-9 (PMC4901105; doi:10.1007/s00125-016-3920-9)
Supplement: Supplementary file 13 — (PDF 32 kb) [file 125_2016_3920_MOESM13_ESM.pdf]

ESM Table 12 Association of genetic risk scores with diabetes risks in CKB

**GRS**	**Weighting**	**Continuous GRS**		**Quartiles of GRS**				
		**OR [95%CI]**	***p***	**Q1 (reference)**	**Q2**	**Q3**	**Q4**	***p* for trend**
GRS-T								
	Unweighted	1.07[1.06-1.08]	$8.94x10^{-109}$	1.00[0.94-1.06]	1.33[1.26-1.40]	1.61[1.53-1.69]	2.03[1.95-2.13]	$6.76x10^{-90}$
	AGEN-T2D	1.07[1.06-1.07]	$1.75x10^{-125}$	1.00[0.94-1.06]	1.36[1.29-1.43]	1.62[1.54-1.70]	2.22[2.13-2.32]	$2.75x10^{-111}$
	DIAGRAMv3	1.07[1.07-1.08]	$1.38x10^{-138}$	1.00[0.94-1.06]	1.30[1.24-1.37]	1.57[1.50-1.65]	2.23[2.14-2.33]	$2.36x10^{-115}$
	MetaboChip	1.07[1.07-1.08]	$4.26x10^{-146}$	1.00[0.94-1.06]	1.35[1.28-1.42]	1.65[1.57-1.73]	2.32[2.22-2.42]	$4.67x10^{-125}$
	TransEthnic	1.08[1.07-1.08]	$4.63x10^{-155}$	1.00[0.94-1.06]	1.33[1.27-1.41]	1.67[1.59-1.75]	2.34[2.25-2.45]	$5.25x10^{-130}$
GRS-BC								
	Unweighted	1.09[1.08-1.10]	$9.65x10^{-93}$	1.00[0.95-1.05]	1.27[1.21-1.34]	1.51[1.43-1.58]	1.97[1.88-2.07]	$2.04x10^{-85}$
	AGEN-T2D	1.09[1.08-1.10]	$4.83x10^{-102}$	1.00[0.94-1.06]	1.19[1.13-1.25]	1.50[1.43-1.57]	2.00[1.92-2.09]	$8.04x10^{-94}$
	DIAGRAMv3	1.10[1.09-1.10]	$5.55x10^{-116}$	1.00[0.94-1.06]	1.25[1.19-1.32]	1.46[1.39-1.53]	2.07[1.99-2.16]	$2.67x10^{-96}$
	MetaboChip	1.10[1.09-1.11]	$9.17x10^{-123}$	1.00[0.94-1.06]	1.27[1.20-1.33]	1.56[1.49-1.64]	2.12[2.03-2.21]	$9.30x10^{-104}$
	TransEthnic	1.10[1.09-1.11]	$2.28x10^{-126}$	1.00[0.94-1.06]	1.27[1.21-1.34]	1.61[1.53-1.69]	2.17[2.08-2.26]	$4.82x10^{-111}$
GRS-IR								
	Unweighted	1.05[1.03-1.07]	$5.97x10^{-9}$	1.00[0.95-1.05]	1.10[1.05-1.16]	1.14[1.08-1.19]	1.22[1.16-1.29]	$3.46x10^{-8}$
	AGEN-T2D	1.04[1.03-1.06]	$1.47x10^{-7}$	1.00[0.95-1.05]	1.05[1.00-1.11]	1.13[1.07-1.18]	1.15[1.10-1.21]	$5.46x10^{-6}$
	DIAGRAMv3	1.05[1.03-1.07]	$1.56x10^{-6}$	1.00[0.95-1.05]	1.13[1.07-1.18]	1.13[1.08-1.19]	1.19[1.13-1.25]	$5.31x10^{-6}$
	MetaboChip	1.06[1.04-1.08]	$2.75x10^{-7}$	1.00[0.95-1.05]	1.11[1.06-1.17]	1.17[1.11-1.23]	1.19[1.14-1.25]	$3.26x10^{-7}$
	TransEthnic	1.06[1.04-1.08]	$7.051x1^{-8}$	1.00[0.95-1.05]	1.08[1.03-1.13]	1.14[1.09-1.20]	1.19[1.14-1.25]	$1.68x10^{-7}$
