# Supplementary material for: Evaluation of type 2 diabetes genetic risk variants in Chinese adults: findings from 93,000 individuals from the China Kadoorie Biobank
Source: Diabetologia. 2016 Apr 6;59:1446–57. doi: 10.1007/s00125-016-3920-9 (PMC4901105; doi:10.1007/s00125-016-3920-9)
Supplement: Supplementary file 14 — (PDF 5 kb) [file 125_2016_3920_MOESM14_ESM.pdf]

ESM Table 13 Proportion of variance in phenotype explained by GRSs

GRS	HOMA-B	HOMA-IR
GRS-B	0.94%	0.13%
GRS-IR	0.03%	0.15%
