# Supplementary material for: Evaluation of type 2 diabetes genetic risk variants in Chinese adults: findings from 93,000 individuals from the China Kadoorie Biobank
Source: Diabetologia. 2016 Apr 6;59:1446–57. doi: 10.1007/s00125-016-3920-9 (PMC4901105; doi:10.1007/s00125-016-3920-9)
Supplement: Supplementary file 15 — (PDF 77 kb) [file 125_2016_3920_MOESM15_ESM.pdf]

## ESM Table 14 GRSs-obesity interaction effects on risk of diabetes

| GRS | Weighting | BMI | | | | | Waist Circumference | | | | | Waist-Hip Ratio | | | | | Percent Body Fat | | | | |
|---|---|---|---|---|---|---|---|---|---|---|---|---|---|---|---|---|---|---|---|---|---|
| | | **Normal weight** | **Overweight** | **Obese** | $p_{\text{interaction}}$ (Strata) | $p_{\text{interaction}}$ (Continuous) | **Low** | **Medium** | **High** | $p_{\text{interaction}}$ (Strata) | $p_{\text{interaction}}$ (Continuous) | **Low** | **Medium** | **High** | $p_{\text{nteraction}}$ (Strata) | $p_{\text{interaction}}$ (Continuous) | **Low** | **Medium** | **High** | $p_{\text{nteraction}}$ (Strata) | $p_{\text{interaction}}$ (Continuous) |
| **Cases/Controls (n)** | | 1,978 / 39,862 | 3,386 / 35,711 | 1,745 / 10,438 | | | 1,067 / 30,046 | 2,017 / 29,075 | 4,025 / 26,890 | | | 1,016 / 30,035 | 2,370 / 32,074 | 3,723 / 23,902 | | | 1,392 / 29,852 | 2,180 / 28,727 | 3,533 / 27,369 | | |
| GRS-T | | | | | | | | | | | | | | | | | | | | | |
| | Unweighted | 1.09 [1.08-1.10] | 1.07 [1.06-1.08] | 1.05 [1.04-1.07] | $9.21x10^{-4}$ * | $7.03x10^{-4}$ * | 1.10[1.08-1.12] | 1.08[1.06-1.09] | 1.06[1.05-1.07] | $1.47x10^{-4}$ * | $1.78x10^{-3}$ * | 1.10[1.08-1.12] | 1.07[1.06-1.08] | 1.06[1.05-1.07] | $2.39x10^{-4}$ * | $3.02x10^{-4}$ * | 1.09[1.08-1.11] | 1.07[1.06-1.08] | 1.06[1.05-1.07] | $8.28x10^{-4}$ * | $6.29x10^{-2}$ |
| | AGEN-T2D | 1.08 [1.07-1.09] | 1.07 [1.06-1.07] | 1.05 [1.04-1.06] | $2.18x10^{-4}$ * | $2.52x10^{-4}$ * | 1.09[1.08-1.11] | 1.07[1.06-1.08] | 1.06[1.05-1.07] | $5.31x10^{-5}$ * | $6.48x10^{-4}$ * | 1.09[1.08-1.11] | 1.07[1.06-1.08] | 1.05[1.05-1.06] | $1.09x10^{-6}$ * | $6.08x10^{-5}$ * | 1.09[1.08-1.11] | 1.06[1.05-1.07] | 1.06[1.05-1.07] | $3.06x10^{-5}$ * | $2.30x10^{-2}$ |
| | DIAGRAMv3 | 1.10 [1.09-1.11] | 1.08 [1.07-1.09] | 1.05 [1.04-1.07] | $3.19x10^{-6}$ * | $5.28x10^{-6}$ * | 1.11[1.09-1.12] | 1.08[1.07-1.09] | 1.06[1.06-1.07] | $4.29x10^{-6}$ * | $1.08x10^{-4}$ * | 1.11[1.09-1.13] | 1.08[1.07-1.09] | 1.06[1.05-1.07] | $8.23x10^{-7}$ * | $6.70x10^{-5}$ * | 1.10[1.09-1.11] | 1.08[1.07-1.09] | 1.06[1.06-1.07] | $2.36x10^{-5}$ * | $1.94x10^{-3}$ * |
| | MetaboChip | 1.10 [1.09-1.11] | 1.08 [1.07-1.09] | 1.06 [1.04-1.07] | $1.01x10^{-5}$ * | $1.00x10^{-5}$ * | 1.11[1.09-1.12] | 1.08[1.07-1.09] | 1.07[1.06-1.07] | $7.70x10^{-6}$ * | $1.47x10^{-4}$ * | 1.11[1.10-1.13] | 1.08[1.07-1.09] | 1.06[1.06-1.07] | $1.26x10^{-6}$ * | $1.88x10^{-4}$ * | 1.10[1.09-1.11] | 1.08[1.07-1.09] | 1.07[1.06-1.07] | $7.75x10^{-5}$ * | $1.88x10^{-3}$ * |
| | **TransEthnic** | 1.10 [1.09-1.11] | 1.08 [1.07-1.09] | 1.06 [1.05-1.07] | $1.45x10^{-5}$ * | $1.17x10^{-5}$ * | 1.11[1.10-1.13] | 1.08[1.07-1.09] | 1.07[1.06-1.08] | $9.08x10^{-6}$ * | $2.60x10^{-4}$ * | 1.11[1.10-1.13] | 1.08[1.07-1.09] | 1.07[1.06-1.07] | $1.38x10^{-6}$ * | $1.19x10^{-4}$ * | 1.10[1.09-1.12] | 1.08[1.07-1.09] | 1.07[1.06-1.08] | $2.86x10^{-5}$ * | $4.74x10^{-3}$ |
| GRS-BC | | | | | | | | | | | | | | | | | | | | | |
| | Unweighted | 1.12 [1.10-1.14] | 1.09 [1.08-1.10] | 1.08 [1.06-1.10] | $1.81x10^{-3}$ * | $1.17x10^{-3}$ * | 1.13[1.11-1.16] | 1.09[1.08-1.11] | 1.08[1.07-1.09] | $4.32x10^{-4}$ * | $1.19x10^{-2}$ | 1.13[1.11-1.15] | 1.10[1.09-1.12] | 1.08[1.06-1.09] | $1.32x10^{-4}$ * | $2.95x10^{-3}$ * | 1.12[1.10-1.14] | 1.10[1.08-1.11] | 1.08[1.07-1.09] | $1.45x10^{-3}$ * | $3.35x10^{-2}$ |
| | AGEN-T2D | 1.12 [1.11-1.14] | 1.09 [1.08-1.11] | 1.08 [1.06-1.10] | $1.90x10^{-3}$ * | $2.08x10^{-3}$ * | 1.14[1.12-1.16] | 1.10[1.08-1.11] | 1.08[1.07-1.10] | $3.07x10^{-4}$ * | $4.91x10^{-3}$ | 1.14[1.11-1.16] | 1.11[1.10-1.13] | 1.07[1.06-1.09] | $1.60x10^{-6}$ * | $2.47x10^{-4}$ * | 1.13[1.11-1.15] | 1.10[1.08-1.11] | 1.08[1.07-1.10] | $1.24x10^{-3}$ * | $4.81x10^{-2}$ |
| | DIAGRAMv3 | 1.13 [1.12-1.15] | 1.10 [1.08-1.11] | 1.07 [1.06-1.09] | $1.17x10^{-5}$ * | $4.71x10^{-6}$ * | 1.15[1.13-1.17] | 1.10[1.08-1.12] | 1.09[1.07-1.10] | $9.85x10^{-6}$ * | $4.02x10^{-4}$ * | 1.14[1.12-1.17] | 1.11[1.09-1.12] | 1.08[1.07-1.09] | $4.15x10^{-6}$ * | $9.00x10^{-4}$ * | 1.13[1.12-1.15] | 1.10[1.09-1.12] | 1.08[1.07-1.09] | $1.90x10^{-5}$ * | $5.63x10^{-4}$ * |
| | MetaboChip | 1.13 [1.12-1.15] | 1.10 [1.09-1.11] | 1.08 [1.06-1.10] | $6.66x10^{-5}$ * | $2.27x10^{-5}$ * | 1.15[1.13-1.17] | 1.10[1.09-1.12] | 1.09[1.08-1.10] | $3.73x10^{-5}$ * | $9.88x10^{-4}$ * | 1.15[1.13-1.17] | 1.11[1.09-1.12] | 1.08[1.07-1.10] | $5.53x10^{-6}$ * | $2.11x10^{-3}$ * | 1.14[1.12-1.16] | 1.11[1.09-1.13] | 1.09[1.07-1.10] | $6.58x10^{-5}$ * | $1.11x10^{-3}$ * |
| | **TransEthnic** | 1.13 [1.11-1.15] | 1.10 [1.09-1.11] | 1.08 [1.06-1.10] | $8.86x10^{-5}$ * | $3.51x10^{-5}$ * | 1.15[1.13-1.17] | 1.10[1.08-1.12] | 1.09[1.08-1.10] | $7.03x10^{-5}$ * | $1.67x10^{-3}$ * | 1.14[1.12-1.17] | 1.11[1.09-1.12] | 1.08[1.07-1.09] | $3.59x10^{-6}$ * | $1.18x10^{-3}$ * | 1.13[1.11-1.15] | 1.11[1.09-1.12] | 1.09[1.07-1.10] | $8.74x10^{-5}$ * | $3.06x10^{-3}$ * |
| GRS-IR | | | | | | | | | | | | | | | | | | | | | |
| | Unweighted | 1.05 [1.02-1.09] | 1.06 [1.03-1.08] | 1.05 [1.01-1.09] | 0.83 | 0.91 | 1.08[1.03-1.12] | 1.05[1.02-1.09] | 1.05[1.02-1.07] | 0.30 | 0.61 | 1.09[1.04-1.14] | 1.04[1.01-1.07] | 1.05[1.02-1.07] | 0.24 | 0.70 | 1.05[1.01-1.09] | 1.05[1.02-1.08] | 1.07[1.04-1.09] | 0.39 | 0.68 |
| | AGEN-T2D | 1.05 [1.01-1.08] | 1.05 [1.02-1.08] | 1.04 [1.00-1.07] | 0.69 | 0.70 | 1.07[1.03-1.12] | 1.05[1.02-1.09] | 1.04[1.01-1.06] | 0.13 | 0.37 | 1.10[1.05-1.15] | 1.03[1.00-1.06] | 1.04[1.01-1.06] | 0.11 | 0.19 | 1.06[1.02-1.10] | 1.03[1.00-1.06] | 1.06[1.03-1.09] | 0.68 | 0.89 |
| | DIAGRAMv3 | 1.06 [1.02-1.09] | 1.05 [1.02-1.08] | 1.03 [0.99-1.07] | 0.32 | 0.83 | 1.07[1.02-1.12] | 1.06[1.03-1.10] | 1.03[1.01-1.06] | 0.17 | 0.67 | 1.11[1.05-1.16] | 1.03[1.00-1.07] | 1.04[1.01-1.06] | 0.07 | 0.68 | 1.05[1.01-1.09] | 1.05[1.02-1.09] | 1.05[1.02-1.08] | 0.97 | 0.93 |
| | MetaboChip | 1.07 [1.03-1.11] | 1.06 [1.03-1.09] | 1.03 [0.99-1.07] | 0.19 | 0.54 | 1.08[1.03-1.14] | 1.07[1.04-1.11] | 1.04[1.01-1.06] | 0.06 | 0.39 | 1.11[1.06-1.17] | 1.03[1.00-1.07] | 1.05[1.02-1.07] | 0.11 | 0.56 | 1.06[1.01-1.11] | 1.05[1.01-1.09] | 1.06[1.03-1.09] | 0.91 | 0.69 |
| | **TransEthnic** | 1.06 [1.03-1.11] | 1.06 [1.03-1.09] | 1.04 [1.00-1.08] | 0.37 | 0.80 | 1.09[1.03-1.14] | 1.07[1.03-1.11] | 1.04[1.01-1.07] | 0.11 | 0.51 | 1.11[1.06-1.17] | 1.04[1.00-1.07] | 1.05[1.02-1.08] | 0.15 | 0.57 | 1.06[1.01-1.11] | 1.05[1.01-1.09] | 1.07[1.04-1.10] | 0.72 | 0.95 |

BMI strata were defined according to Asian criteria proposed by WHO: Normal weight, <23 kg/m$^2$; Overweight, 23-27.5 kg/m$^2$; Obese, ≥27.5 kg/m$^2$

Sex-specific tertiles were used to define:

waist circumference strata (Low, male <76.9 cm, female < 74.4 cm; Medium male ≥76.9-86.1 cm, female ≥74.4-82.8 cm; High, male≥86.1 cm, female ≥82.8 cm),

Waist-hip ratio strata (Low, male <0.88, female <0.84; Medium male ≥0.88-0.94, female ≥0.84-0.91; High, male≥0.94, female ≥0.91),

Percent body fat (Low, male <18.8%, female <28.8%; Medium male ≥18.8-24.5%, female ≥28.8-34.9%; High, male≥24.5%, female ≥34.9%)

*$p$ <0.05/12=0.0042
