# Supplementary material for: Evaluation of type 2 diabetes genetic risk variants in Chinese adults: findings from 93,000 individuals from the China Kadoorie Biobank
Source: Diabetologia. 2016 Apr 6;59:1446–57. doi: 10.1007/s00125-016-3920-9 (PMC4901105; doi:10.1007/s00125-016-3920-9)
Supplement: Supplementary file 16 — (PDF 36 kb) [file 125_2016_3920_MOESM16_ESM.pdf]

ESM Table 15 Associations between GRSs and adiposity measurements

GRS	Weighting	BMI		Waist Circumference		Waist-Hip Ratio		Percent Body Fat	
		**β (SE), kg/m^2**	***p***	**β (SE), cm**	***p***	**β (SE)**	***p***	**β (SE), %**	***p***
GRS-T									
	Unweighted	-0.005(0.003)	3.86×10^{-2}	-0.007(0.007)	3.48×10^{-1}	0.0001(0.0001)	7.52×10^{-2}	-0.010(0.005)	4.67×10^{-2}
	AGEN-T2D	-0.006(0.002)	7.62×10^{-3}	-0.006(0.006)	3.62×10^{-1}	0.0001(0.0000)	8.36×10^{-2}	-0.008(0.004)	6.36×10^{-2}
	DIAGRAMv3	-0.009(0.002)	9.12×10^{-5}	-0.013(0.007)	6.10×10^{-2}	0.0001(0.0000)	6.66×10^{-2}	-0.015(0.005)	1.63×10^{-3}
	MetaboChip	-0.008(0.002)	4.04×10^{-4}	-0.010(0.007)	1.54×10^{-1}	0.0001(0.0000)	2.14×10^{-2}	-0.014(0.005)	4.59×10^{-3}
	**TransEthnic**	-0.010(0.002)	2.91×10^{-5}	-0.013(0.007)	5.30×10^{-2}	0.0001(0.0000)	5.89×10^{-2}	-0.015(0.005)	1.29×10^{-3}
GRS-BC									
	Unweighted	-0.015(0.004)	1.46×10^{-5}	-0.018(0.010)	7.51×10^{-2}	0.0001(0.0001)	1.26×10^{-1}	-0.019(0.007)	7.33×10^{-3}
	AGEN-T2D	-0.019(0.003)	6.34×10^{-8}	-0.022(0.010)	2.62×10^{-2}	0.0001(0.0001)	1.75×10^{-1}	-0.023(0.007)	9.26×10^{-4}
	DIAGRAMv3	-0.022(0.003)	6.28×10^{-11}	-0.033(0.010)	7.54×10^{-4}	0.0001(0.0001)	4.52×10^{-1}	-0.029(0.007)	2.30×10^{-5}
	MetaboChip	-0.023(0.003)	2.15×10^{-11}	-0.034(0.010)	4.13×10^{-4}	0.0000(0.0001)	5.22×10^{-1}	-0.030(0.007)	1.68×10^{-5}
	**TransEthnic**	-0.022(0.003)	2.14×10^{-11}	-0.031(0.009)	9.73×10^{-4}	0.0001(0.0001)	3.36×10^{-1}	-0.029(0.007)	1.69×10^{-5}
GRS-IR									
	Unweighted	-0.011(0.008)	1.40×10^{-1}	-0.014(0.021)	5.26×10^{-1}	0.0000(0.0002)	9.42×10^{-1}	-0.054(0.015)	3.79×10^{-4}
	AGEN-T2D	-0.005(0.007)	4.57×10^{-1}	-0.001(0.021)	9.52×10^{-1}	0.0000(0.0001)	8.26×10^{-1}	-0.041(0.015)	4.88×10^{-3}
	DIAGRAMv3	-0.018(0.009)	3.61×10^{-2}	-0.041(0.023)	7.48×10^{-2}	-0.0002(0.0002)	1.40×10^{-1}	-0.067(0.016)	3.48×10^{-5}
	MetaboChip	-0.016(0.009)	8.80×10^{-2}	-0.031(0.024)	1.99×10^{-1}	-0.0002(0.0002)	3.05×10^{-1}	-0.069(0.017)	5.04×10^{-5}
	**TransEthnic**	-0.016(0.009)	7.82×10^{-2}	-0.028(0.024)	2.58×10^{-1}	-0.0001(0.0002)	5.32×10^{-1}	-0.070(0.017)	5.05×10^{-5}

Adjusting for age, age^2, sex and region
