# Supplementary figures and images for: Evaluation of type 2 diabetes genetic risk variants in Chinese adults: findings from 93,000 individuals from the China Kadoorie Biobank
Source: Diabetologia. 2016 Apr 6;59:1446–57. doi: 10.1007/s00125-016-3920-9 (PMC4901105; doi:10.1007/s00125-016-3920-9)

**ESM Fig. 1 Information of studies providing different weight for each variant.**

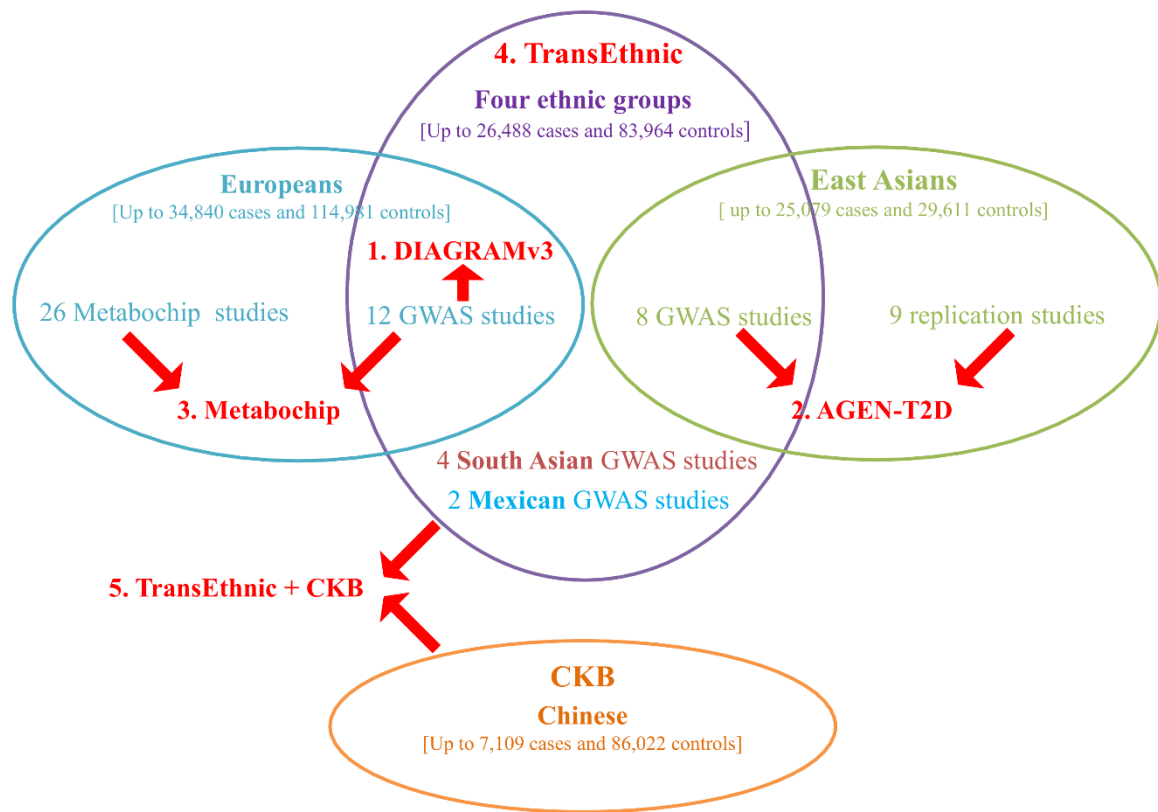

Supplement: Supplementary file 17 — (PDF 216 kb) [file 125_2016_3920_MOESM17_ESM.pdf]
