# Supplementary material for: Evaluation of type 2 diabetes genetic risk variants in Chinese adults: findings from 93,000 individuals from the China Kadoorie Biobank
Source: Diabetologia. 2016 Apr 6;59:1446–57. doi: 10.1007/s00125-016-3920-9 (PMC4901105; doi:10.1007/s00125-016-3920-9)

ESM Fig. 3 Comparison of risk allele frequencies between Chinese and Europeans. Risk allele frequencies in Europeans were obtained from 1000 Genomes Project Phase 3 and correlation was calculated as Pearson's correlation coefficients (r).

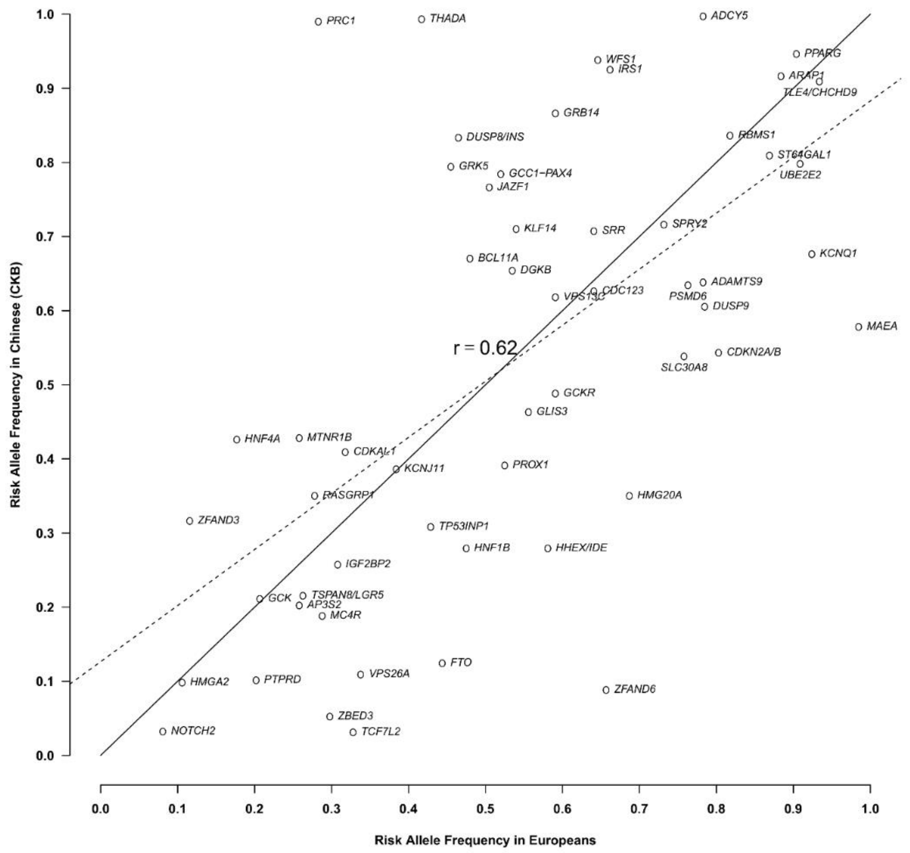

Supplement: Supplementary file 19 — (PDF 147 kb) [file 125_2016_3920_MOESM19_ESM.pdf]
