# Supplementary material for: Evaluation of type 2 diabetes genetic risk variants in Chinese adults: findings from 93,000 individuals from the China Kadoorie Biobank
Source: Diabetologia. 2016 Apr 6;59:1446–57. doi: 10.1007/s00125-016-3920-9 (PMC4901105; doi:10.1007/s00125-016-3920-9)

ESM Fig. 4. Distribution of genetic risk scores and associations with T2D in the CKB study. The histograms represent the distribution of the un-weighted GRS. Individuals were grouped based on quartiles of TransEthinic weighted genetic risk scores. The odds ratios associated with each additional point of the TransEthinic weighted GRSs, which corresponds to one additional risk allele, are plotted (back diamond).

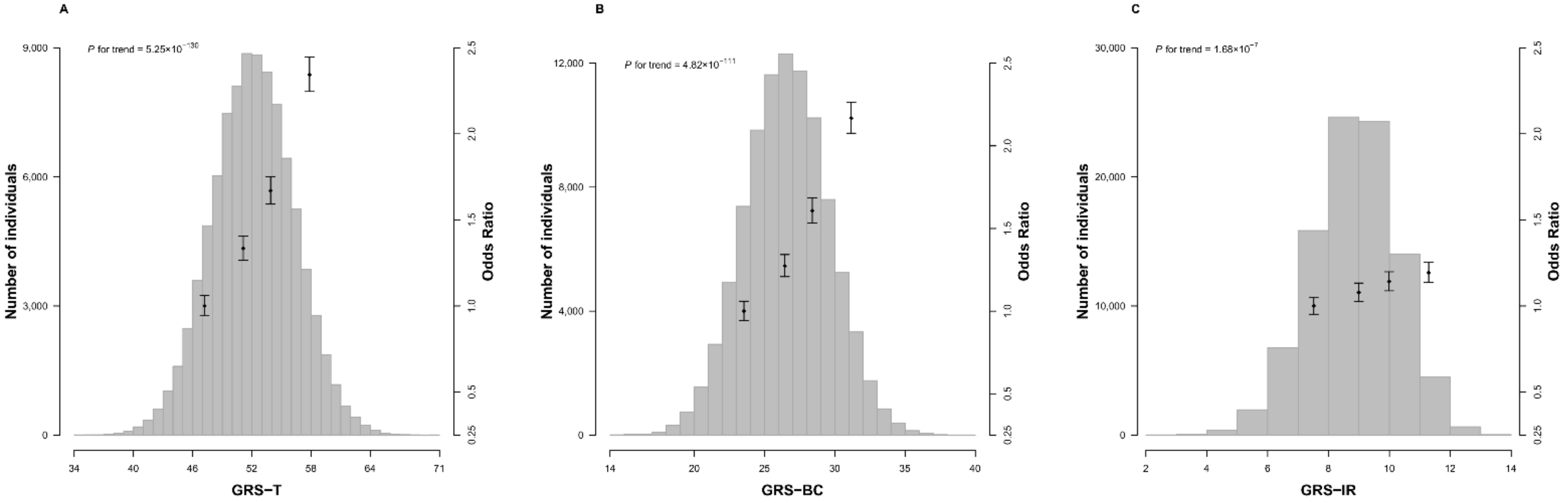

Supplement: Supplementary file 20 — (PDF 120 kb) [file 125_2016_3920_MOESM20_ESM.pdf]
