# Supplementary material for: Evaluation of type 2 diabetes genetic risk variants in Chinese adults: findings from 93,000 individuals from the China Kadoorie Biobank
Source: Diabetologia. 2016 Apr 6;59:1446–57. doi: 10.1007/s00125-016-3920-9 (PMC4901105; doi:10.1007/s00125-016-3920-9)

ESM Fig. 2 Comparison of allelic effect sizes between CKB study and AGEN-T2D GWAS meta-analysis study (A), meta-analysis study of CKB+AGEN-T2D and DIAGRAMv3 GWAS meta-analysis study (B). Loci showing evidence of heterogeneity are highlighted in grey (<0.05) or black (correction for multiple comparisons: < 0.05/(55 × 3) =3.0×10^{-4}). The solid lines represents the line of equality and the result of inverse-variance weighted least squares regression through the origin is shown as a broken line, with the shaded area representing the 95% confidence interval.

(a)

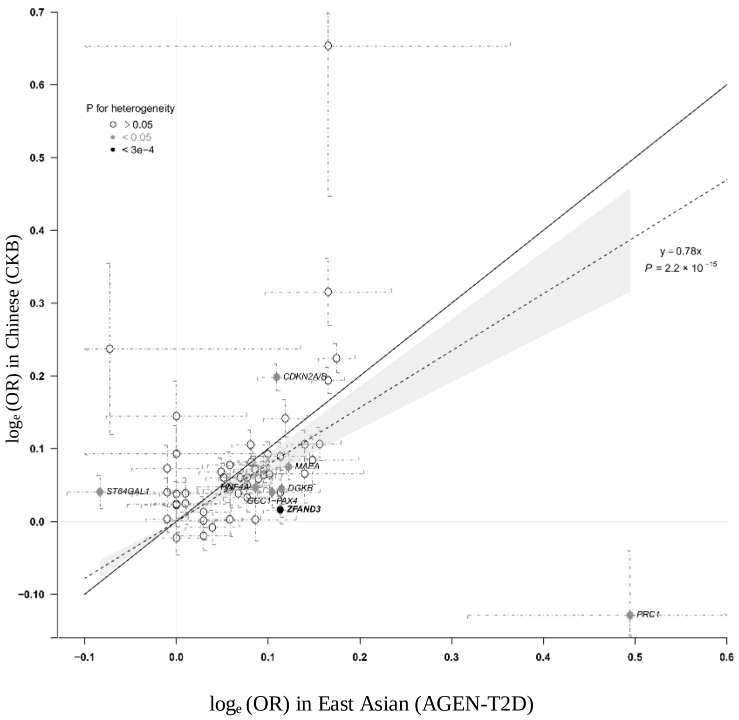

(b)

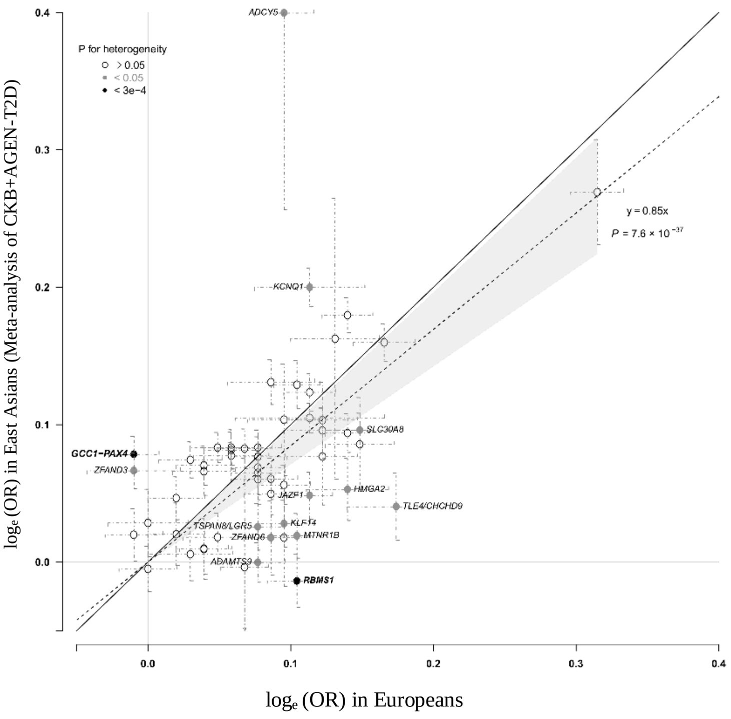

Supplement: Supplementary file 18 — (PDF 191 kb) [file 125_2016_3920_MOESM18_ESM.pdf]
